# Increasing awareness of HIV pre‐exposure prophylaxis (PrEP) and willingness to use HIV PrEP among men who have sex with men: a systematic review and meta‐analysis of global data

**DOI:** 10.1002/jia2.25883

**Published:** 2022-03-07

**Authors:** Zhishan Sun, Qianfei Gu, Yifan Dai, Huachun Zou, Bruce Agins, Qiaosen Chen, Peiyang Li, Junchun Shen, Yi Yang, Hongbo Jiang

**Affiliations:** ^1^ Department of Epidemiology and Biostatistics School of Public Health Guangdong Pharmaceutical University Guangzhou PR China; ^2^ School of Public Health (Shenzhen) Sun Yat‐sen University Shenzhen PR China; ^3^ Kirby Institute University of New South Wales Sydney New South Wales Australia; ^4^ School of Public Health Shanghai Jiao Tong University Shanghai PR China; ^5^ HEALTHQUAL Institute for Global Health Sciences University of California San Francisco California USA; ^6^ School of Public Health Sun Yat‐sen University Guangzhou PR China; ^7^ Guangdong Provincial Engineering Research Center of Public Health Detection and Assessment Guangdong Pharmaceutical University Guangzhou PR China

**Keywords:** men who have sex with men, pre‐exposure prophylaxis, awareness, willingness, trend, meta‐analysis

## Abstract

**Introduction:**

Integrated knowledge regarding pre‐exposure prophylaxis (PrEP) awareness and willingness to use PrEP can be useful for HIV prevention in high incidence groups. This review summarizes the awareness of PrEP and willingness to use PrEP among men who have sex with men (MSM).

**Methods:**

Online electronic databases were searched before 31 August 2021. A meta‐analysis was conducted to pool studies analysing PrEP awareness and willingness to use PrEP. LOESS regression and linear regression were applied to fit the trends over time for the proportion of MSM aware of PrEP and willing to use PrEP. Dose–response meta‐analysis (DRMA) was conducted by a restricted cubic spline model to explore the relationship between willingness to use PrEP and selected factors.

**Results and Discussion:**

A total of 156 articles involving 228,403 MSM were included. The pooled proportions of MSM aware of PrEP and willing to use PrEP were 50.0 (95% CI: 44.8–55.2) and 58.6% (95% CI: 54.8–62.4), respectively. PrEP awareness varied among countries with different economic status and different WHO regions, among different publication and research years, PrEP types and support policies. PrEP willingness differed among countries with different economic status and groups with different risks of HIV. The awareness of PrEP increased from 2007 to 2019 with a slope of 0.040260 (*p*<0.0001), while the proportion of MSM willing to use PrEP decreased from 2007 to 2014 (slope = –0.03647, *p* = 0.00390) but increased after 2014 (slope = 0.04187, *p* = 0.03895). The main facilitators of willingness to use PrEP were PrEP awareness, condomless sexual behaviours, high perceived risk of HIV infection and influence of social network. The main barriers were doubts about the efficacy and side effects of PrEP. DRMA results indicated that MSM with more sexual partners and lower level of education were more willing to use PrEP. No publication bias was observed.

**Conclusions:**

The proportions of PrEP awareness and willingness to use PrEP among MSM have increased since 2014, although the awareness was low and the willingness was moderate. Improving awareness of PrEP through increasing access to PrEP‐related health education and enhancing risk perceptions of HIV infection could have positive effects on the willingness to use PrEP among MSM.

## 1 INTRODUCTION

Condomless sexual behaviour between men is a well‐recognized route of HIV transmission. In 2019, marginalized populations, such as men who have sex with men (MSM), transgender women (TG) and their corresponding sexual partners, accounted for 62% of new HIV infections globally [1].

Several randomized clinical trials have shown that tenofovir disoproxil fumarate (TDF)/emtricitabine (FTC) and FTC/tenofovir alafenamide used as pre‐exposure prophylaxis (PrEP) significantly decreased the likelihood of HIV infection among MSM, TG and other high incidence groups [2, 3, 4, 5]. World Health Organization (WHO) has first recommended that, in addition to HIV testing, condom use, screening and treatment of sexually transmitted infections (STIs), policy makers should also routinely incorporate PrEP into prevention programs [6]. In 2015, WHO formally recommended providing PrEP to MSM [7]. Four years later, 44 countries and regions had approved the use of PrEP for HIV prevention [8]. To achieve its promise, PrEP must be both acceptable to users, available and implemented in practice.

Providing effective PrEP services for high incidence groups is a critical measure to reduce new HIV infections [7]. Many researchers, health providers, policy makers and community leaders/members are committed to enhancing awareness of PrEP and willingness to engage in PrEP among high incidence groups. A previous meta‐analysis has found low proportions of MSM were aware of PrEP and willing to use PrEP in low‐ and middle‐income countries (LMICs) [9]. Another meta‐analysis summarized the acceptability of PrEP among MSM on the global scale, including articles which were published before July 2016 [10]. However, to our knowledge, there are few large‐sample systematic reviews addressing both the awareness of PrEP and willingness to use PrEP among MSM on the global scale, and few studies have analysed global trends in the awareness of PrEP and willingness to use PrEP over time. More knowledge about the integration of awareness and willingness can be useful for future HIV prevention in high incidence groups.

We conducted this systematic review and meta‐analysis to summarize the global research on the awareness of PrEP, the willingness to use PrEP among MSM and their integration in the literature. In addition, we sought to analyse the temporal trends in awareness of PrEP and willingness to use PrEP and examine the factors associated with willingness to use PrEP among MSM.

## 2 METHODS

This meta‐analysis was conducted in accordance with the Preferred Reporting Items for Systematic Review and Meta‐Analyses (PRISMA) guidelines [11]. The PRISMA checklist is appended as File S1.

### 2.1 Data search strategy

Web of Science, Embase, PubMed and Cochrane Library were searched to identify literature on awareness of PrEP and willingness to use HIV PrEP among MSM. A combination of relevant keywords and medical subject heading (MeSH) terms were adopted to conduct the literature search using Boolean operators, truncations and synonym extensions, as appropriate. Detailed search strings used in each database are shown in File S2.

### 2.2 Eligibility criteria

#### 2.2.1 Inclusion criteria

The inclusion criteria were as follows: (1) cross‐sectional studies, cohort studies, case control study, qualitative studies and mixed method studies; (2) studies reporting data generated from HIV‐negative MSM (including gay, bisexual, male sex workers and TG); for studies reporting data separately for MSM and other populations, such as heterosexual male or female sex workers, only data related to MSM were considered and extracted; (3) studies reporting awareness or willingness to use PrEP; and (4) studies that were peer‐reviewed before 31 August 2021.

#### 2.2.2 Exclusion criteria

The exclusion criteria were as follows: (1) non‐original research, secondary reports, commentaries, editorials, reviews and duplicates; (2) studies focusing on other populations (e.g. women, heterosexual couples or primary care providers) rather than MSM; (3) studies reporting no results for a segregated subgroup of MSM or TG; (4) studies reporting no findings related to PrEP; (5) studies reporting results that cannot be used to calculate the proportion of MSM aware of and willing to use PrEP; and (6) studies focusing on other outcomes.

### 2.3 Study selection

**Figure 1:**
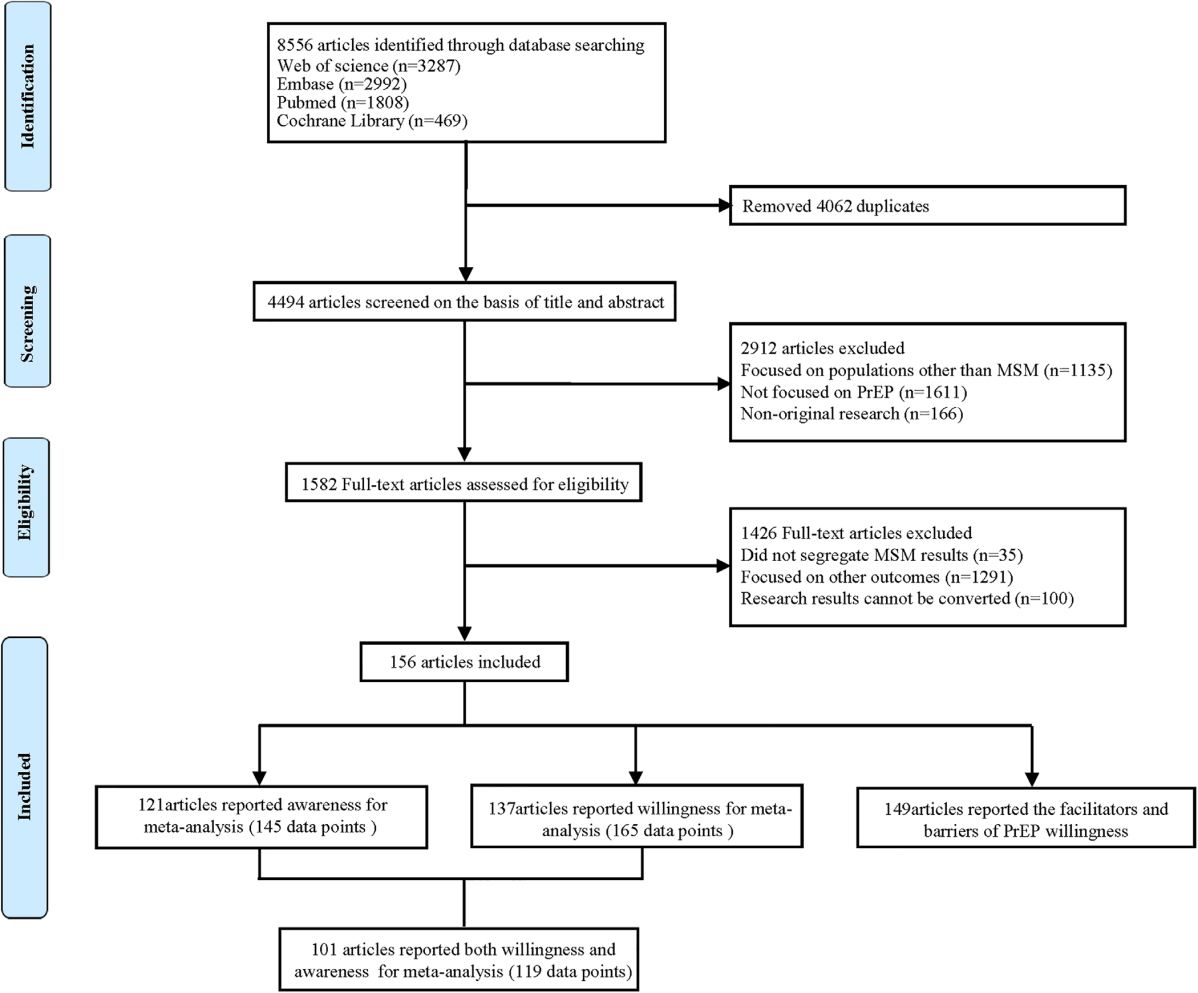
Flow diagram of search strategy and study selection.

Two review team members independently screened references in two stages. In the first stage, two reviewers screened the titles and abstracts to exclude unrelated studies. They further assessed full‐text papers for eligibility in the second stage. A flowchart illustrated the literature selection procedure (Figure 1). All references retrieved according to selection criteria were classified as eligible or ineligible at each stage. A third reviewer resolved discrepancies in the literature inclusion between the two reviewers. Three review team members discussed together to reach a consensus.

### 2.4 Data collection process

Data extracted independently by two reviewers from the aforementioned two stages were imported into Microsoft Excel Software. The following data were entered into a standardized form: authors, year of publication, research year, country or region of study, study design, sample size, study population characteristics (MSM/TG, primary sex role and high incidence groups), recruitment settings, types of PrEP, policy support, proportion of MSM aware of and MSM willing to use PrEP, and factors associated with willingness to use PrEP. The data were exported into Excel, and then the third reviewer compared the two independent data forms. Three researchers discussed any disagreements until a consensus was reached.

### 2.5 Quality assessment

The quality of eligible quantitative studies and mixed methods studies was evaluated by using the Effective Public Health Practice Project (EPHPP) Quality Assessment Tool (http://www.ephpp.ca/tools.html) [12, 13]. Study quality was considered strong if none of the six component ratings was weak. Study quality was considered moderate if one component rating was weak and was considered weak if two or more component ratings were weak. To assess the quality of qualitative studies, we used the Qualitative Research Checklist from the Critical Appraisal Skills Program (CASP) [14]. Based on the total score of the 10 items, we ranked studies as weak (<5), moderate (5–7) or strong (≥8).

### 2.6 Statistical analysis

Due to different study designs of included studies, we assumed a high potential for heterogeneity between included studies, and thus a random effect meta‐analysis was performed to pool the proportion of awareness of PrEP and willingness to use PrEP [15]. Cochran's *Q* and *I* ^2^ statistic were used to assess the heterogeneity among included studies. For the *Q* statistic, a *p*‐value of greater than 0.10 suggested no significant heterogeneity. *I* ^2^ statistics of 25%, 50% and 75% were classified as low, moderate and high, respectively [16]. To explore the potential source of heterogeneity, subgroup analyses were carried out according to the main relevant variables, such as age, sample size, country/regions, year of study, year of publication, study population, primary sex role, high incidence groups, recruitment settings, types of PrEP and policy support. Publication bias was assessed through funnel plots with Begg's test. Trend analyses were conducted to determine the variation in awareness of PrEP and willingness to use PrEP. Locally estimated scatterplot smoothing (LOESS) regression and linear regression were applied to fit the trend for the proportion of MSM aware of PrEP. The sample size of every data point was used as the weight of the two models. Regarding willingness, we applied grid search using the Akaike information criterion (AIC) to obtain the optimal breakout point for the segmented linear model. The odds ratio (OR) and the corresponding 95% confidence interval (CI) of factors associated with willingness to use PrEP were pooled to summarize the facilitators and barriers. Dose–response meta‐analysis (DRMA) was used to pool the association between factors that contained at least three levels of categories and willingness to use PrEP. The level of education was transformed into years of education, and annual income was converted into monthly income ($) for analysis. The two‐stage DRMA proposed by Berlin et al. [17] was conducted by modelling factors with restricted cubic splines (RCS) of three knots at the 10%, 50% and 90% percentiles of the distribution to estimate the potential trend between factors and ORs related to PrEP willingness. The command “Wald test” in R was used to test whether the slopes of the RCS model for each dose level had a significant difference. If the slopes of each dose level had a significant difference, the dose–response curve had non‐linearity [18]. All analyses were performed by R (version 4.0.3).

## 3 RESULTS AND DISCUSSION

### 3.1 Study characteristics

We initially retrieved 8556 articles. Based on the inclusion criteria, we included 156 articles involving 228,403 MSM, among which 145 data points were related to awareness of PrEP and 165 data points were related to willingness to use PrEP (Figure 1). Articles included in this study were published between 2009 and August 2021 with a median sample size of 644 (interquartile range: 222, 866) ranging from 20 to 39,670. One hundred and forty‐two study populations included MSM, 10 articles exclusively included TG and four articles included MSM and TG. A total of 130 cross‐sectional articles, 11 cohort articles, nine mixed method articles and six qualitative articles were included, among which 87 articles were conducted in high‐income countries (HIC), 67 articles were conducted in LMIC and one study was conducted across 145 countries and two studies were conducted in both LMIC and HIC. The participants' characteristics, and the facilitators and barriers associated with the willingness to use PrEP are presented in Table A1. Study characteristics are given in Appendix A.

### 3.2 Overall awareness of PrEP and willingness to use PrEP and heterogeneity testing

**Figure B1:**
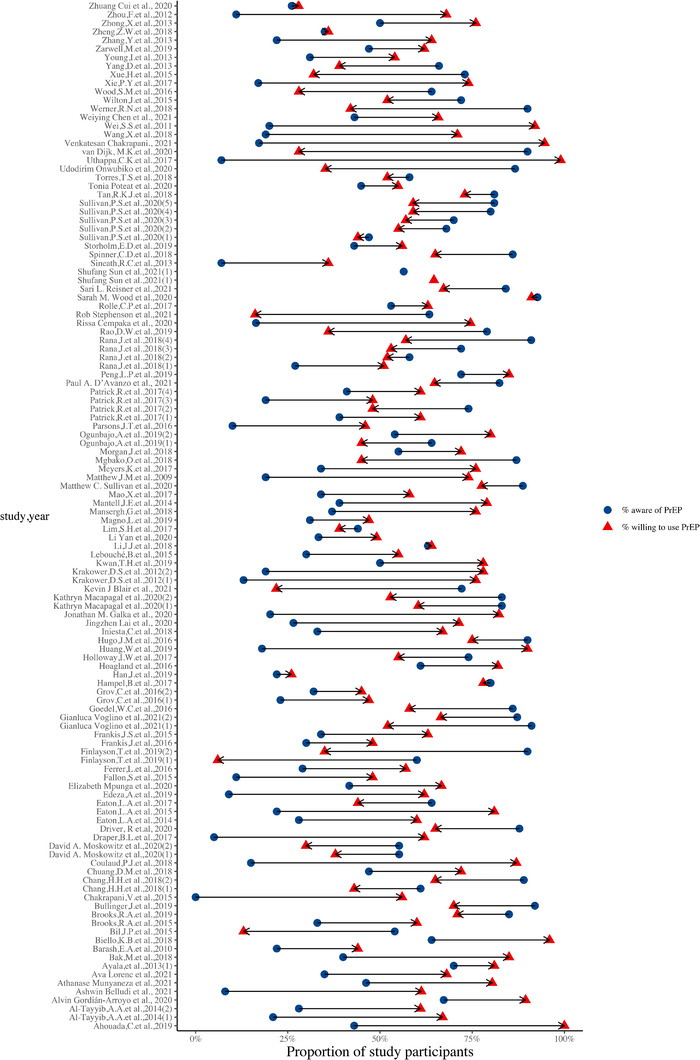
Studies reporting both awareness and willingness to use PrEP.

High heterogeneity (awareness: *p*<0.001, *I* ^2^ = 99.9%; willingness: *p*<0.001, *I* ^2^ = 99.7%) was presented among the included studies. Awareness of PrEP ranged from 0% to 96.7%, with a pooled estimate of 50.0 (95% CI: 44.8–55.2). Willingness to use PrEP ranged from 5.7% to 100%, and the pooled estimate was 58.6% (95% CI: 54.8–62.4). Among 119 data points simultaneously reporting willingness and awareness, the willingness was higher than awareness at 70 data points, whereas willingness was lower than awareness at 49 data points, which are presented in Figure B1. Studies reporting both awareness of PrEP and willingness to use PrEP are presented in Appendix B. Subgroup analysis for awareness (Table 1) indicated that awareness before 2015 was lower than that after 2014 (*p* = 0.0003). Awareness in studies published before 2018 was lower than that in studies published after 2017 (*p* = 0.0004). A significant difference was presented between HIC and LMIC (*p*<0.0001). Countries or regions that had approved TDF/FTC for HIV prevention showed higher PrEP awareness than those without drug approval (*p*<0.0001). Awareness varied among different WHO regions (*p*<0.0001), among which Europe had the highest awareness and South East Asia had the lowest awareness. In addition, awareness varied among different PrEP types (*p*<0.0001). MSM had the highest awareness of daily oral PrEP, followed by on‐demand PrEP and long‐acting injectable (LAI)‐PrEP. Regarding the subgroup analysis for willingness (Table 2), the results showed that MSM in LMIC were more willing to use PrEP than those in HIC (*p* = 0.0296). MSM in high incidence groups were more willing to use PrEP (*p* = 0.0027).

**Table 1:** Subgroup analysis for HIV pre‐exposure prophylaxis awareness among men who have sex with men

Subgroup	No. of study data points	No. of awareness/sample size	Pooled estimate on awareness (%) (95% CI)		Heterogeneity (*I* ^2^, %)	*p*‐Value between groups
Age				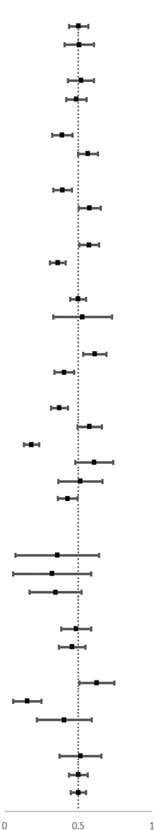		
<35	102	33,782/90,645	50.1 (43.5–56.6)		99.90%	0.9475
≥35	38	39,342/91,209	50.5 (40.6–60.4)		99.90%	
Sample size						
≤400	66	7167/13,937	51.8 (43.0–60.6)		99.60%	0.5732
>400	79	67,925/172,012	48.6 (41.8–55.4)		99.90%	
Publication year						
≤2017	53	12,839/32,111	38.9 (32.0–45.8)		99.60%	0.0004
>2017	92	62,253/153,838	56.4 (49.7–63.2)		99.90%	
Research year						
≤2014	59	24,644/76,281	39.2 (32.9–45.6)		99.80%	0.0003
>2014	81	49,006/106,167	57.5 (50.1–64.9)		99.90%	
Country						
HIC^a^	93	58,989/123,030	57.2 (50.6–63.8)		99.90%	<0.0001
LMIC^b^	50	13,945/59,891	36.0 (30.6–41.3)		99.70%	
Overall population						
MSM^c^	134	73,269/182,798	49.8 (44.4–55.2)		99.90%	0.7808
TG^d^	11	1823/3151	52.7 (32.9–72.6)		99.50%	
Policy support^e^						
Yes	67	49,739/98,028	61.1 (53.2–69.0)		99.90%	<0.0001
No	65	19,210/57,270	40.3 (33.8–46.9)		99.80%	
WHO region						
Western Pacific	32	14,857/58,680	37.0 (31.3–42.7)		99.70%	<0.0001
Americas	73	49,137/100,015	57.5 (49.2–65.7)		99.90%	
South East Asia	11	1031/10,799	18.1 (12.8–23.4)		97.80%	
Europe	17	6769/10,867	60.7 (47.9–73.6)		99.70%	
Africa	10	1208/2596	51.3 (36.4–66.3)		98.50%	
Eastern Mediterranean	1	93/218	42.7 (36.1–49.2)			
Primary sex role						
Receptive	3	162/476	35.7 (7.2–64.1)		98.20%	0.9824
Insertive	3	175/536	32.1 (5.5–58.7)		98.30%	
Versatile	3	155/486	34.5 (16.9–52.2)		94.70%	
High incidence groups^f^						
Yes	34	3911/23,895	48.5 (38.4–58.6)		99.40%	0.6879
No	30	2203/6486	45.7 (36.6–54.8)		98.50%	
PrEP type						
Daily oral PrEP	18	4673/7450	62.4 (50.5–74.2)		99.50%	<0.0001
LAI‐PrEP^g^	3	138/1168	15.3 (5.7–24.9)		96.60%	
On‐demand PrEP	2	383/968	40.3 (21.8–58.8)		97.30%	
Recruitment setting						
Clinic‐based	23	4561/9218	51.4 (37.2–65.6)		99.70%	0.8584
Non‐clinic‐based	93	64,687/165,265	50.0 (43.6–56.4)		99.90%	
Overall	145	75,092/185,949	50.0 (44.8–55.2)		99.90%	

^a^ High‐income countries.

^b^ Low‐ and middle‐income countries.

^c^ Men who have sex with men.

^d^ Transgender women.

^e^ During the research period, whether the national or regional government had approved TDF/FTC for HIV prevention.

^f^ High‐incidence groups were defined as: discontinuous condom use, condomless intercourse, two or more sexual partners, HIV‐positive sexual partners, group sex and multiple non‐regular sexual partners.

^g^ Long‐acting injectable.

**Table 2:** Subgroup analysis for willingness to use HIV pre‐exposure prophylaxis among men who have sex with men

Subgroup	No. of study data points	No. of willingness/sample size	Pooled estimate on willingness (%) (95% CI)		Heterogeneity (*I* ^2^, %)	*p*‐Value between groups
Age				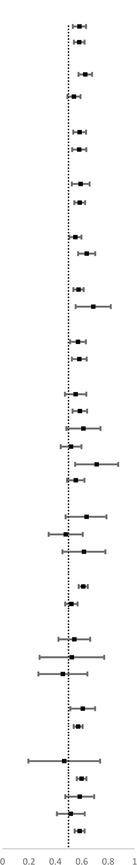		
<35	117	62,950/115,950	58.8 (53.7–63.8)		99.80%	0.8473
≥35	42	31,583/50,481	58.1 (54.1–62.2)		98.90%	
Sample size						
≤400	81	10,995/17,687	62.7 (57.6–67.8)		99.00%	0.0265
>400	84	85,372/152,081	54.7 (49.7–59.6)		99.80%	
Publication year						
≤2017	75	33,304/54,557	58.4 (53.6–63.3)		99.40%	0.9384
>2017	90	63,063/115,211	58.7 (53.3–64.2)		99.80%	
Research year						
≤2014	71	49,819/84,311	59.2 (52.5–66)		99.80%	0.9711
>2014	90	45,706/83,535	59.1 (54.9–63.3)		99.40%	
Country						
HIC^a^	100	66,559/116,959	55.1 (50.5–59.7)		99.70%	0.0296
LMIC^b^	61	26,944/49,015	64.0 (57.5–70.6)		99.70%	
Overall population						
MSM^c^	155	94,519/166,984	57.9 (54.0–61.9)		99.70%	0.1294
TG^d^	10	1848/2784	68.7 (55.4–82.0)		98.80%	
Policy support^e^						
Yes	72	47,264/84,843	57.2 (51.2–63.2)		99.80%	0.6895
No	81	30,405/55,295	58.9 (53.2–64.6)		99.60%	
WHO region						
Western Pacific	37	13,907/29,108	56.7 (48.3–65.1)		99.60%	0.3588
Americas	75	60,302/103,922	58.6 (53.1–64.1)		99.80%	
South East Asia	14	9743/16,369	61.3 (48.4–74.2)		100.00%	
Europe	24	7980/14,355	51.9 (44.1–59.8)		99.00%	
Africa	11	1491/2229	71.3 (55.0–87.6)		99.20%	
Eastern Mediterranean	1	121/218	55.5 (48.9–62.1)			
Primary sex role						
Receptive	12	1153/1634	63.7 (48.7–78.7)		99.20%	0.2271
Insertive	9	2599/6351	47.9 (35.0–60.8)		97.90%	
Versatile	8	3235/6768	61.7 (45.4–77.9)		99.10%	
High incidence groups^f^						
Yes	128	29,857/51,744	61.2 (57.7–64.6)		98.50%	0.0027
No	97	18,436/34,996	52.2 (47.5–56.9)		99.00%	
PrEP type						
Daily oral PrEP	20	3819/6925	54.4 (42.4–66.4)		99.30%	0.7459
LAI‐PrEP^g^	9	2087/3952	52.5 (28.1–77.0)		99.70%	
On‐demand PrEP	9	1362/2937	45.7 (27.2–64.3)		99.30%	
Recruitment setting						
Clinic‐based	34	8424/13,990	60.8 (51.4–70.1)		99.60%	0.4932
Non‐clinic‐based	107	84,567/152,195	57.3 (53.9–60.7)		99.50%	
Denominator of the willingness						
Aware of PrEP	3	892/1747	46.8 (19.6–73.9)		99.20%	0.3615
Awareness after introduction	104	62,106/106,063	60.5 (56.8–64.1)		99.40%	
Awareness unknown	36	22,619/40,316	58.5 (47.6–69.4)		99.90%	
Awareness not reported	22	10,750/21,642	51.7 (41.2–62.3)		99.70%	
Overall	165	96,367/169,768	58.6 (54.8–62.4)		99.70%	

^a^ High‐income countries.

^b^ Low‐ and middle‐income countries.

^c^ Men who have sex with men.

^d^ Transgender women.

^e^ During the research period, whether the national or regional government had approved TDF/FTC for HIV prevention.

^f^ High‐incidence groups were defined as: discontinuous condom use, condomless intercourse, two or more sexual partners, HIV‐positive sexual partners, group sex and multiple non‐regular sexual partners.

^g^ Long‐acting injectable.

### 3.3 Publication bias

**Figure B2:**
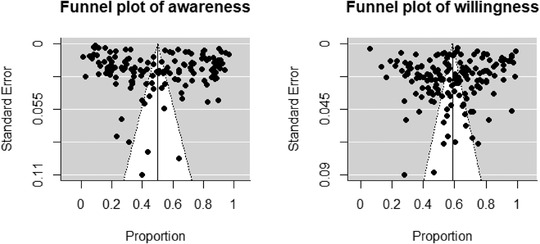
Funnel plots for publication bias of awareness and willingness to use PrEP.

The funnel plots (Figure B2 Funnel plots for publication bias of awareness of PrEP and willingness to use PrEP in Appendix B) were roughly symmetrical. No significant publication bias was observed for PrEP awareness (*p* = 0.1945) or willingness (*p* = 0.41301) according to Begg's test.

### 3.4 Trend analysis of PrEP awareness and willingness

**Figure 2:**
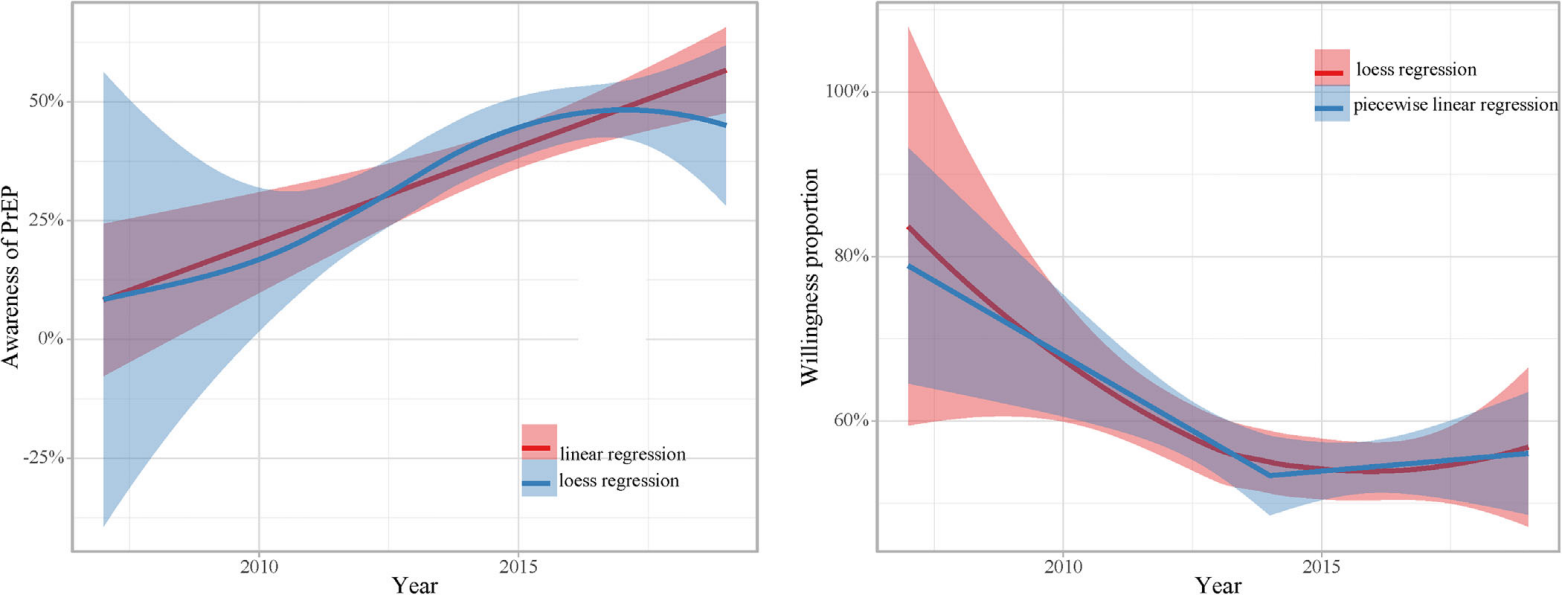
Trend analysis for awareness of and willingness to use PrEP. Simple linear regression and loess regression are used to fit the proportion of PrEP awareness. Piecewise linear regression and loess regression are used to fit the proportion of PrEP willingness. The weights of all regression models are the sample size of every data point. 95% confidence intervals are illustrated as red and blue shade areas. The median research year of every study was selected as the estimated year.

**Figure B3:**
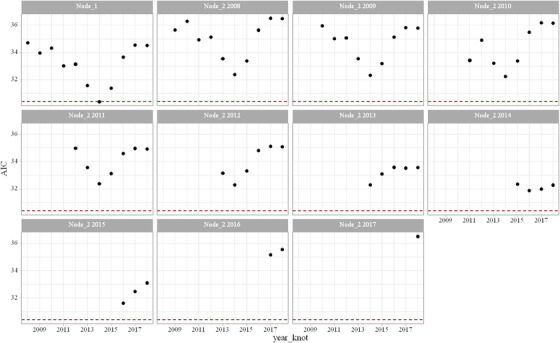
AIC result of PrEP willingness in two piecewise linear regression models. Node_1: The piecewise linear regression model has one turning year. Node_2: The piecewise linear regression model has two turning years. The red line shows the minimum value of AIC.

The trend in PrEP awareness increased from 2007 to 2019, with a slope of 0.040260 (*p*<0.0001). The minimum AIC was found when the piecewise linear model had one turning point in 2014 (Figure B3 AIC results in two piecewise linear regression models in Appendix B). The trend in willingness decreased from 2007 to 2014 (slope = –0.036476, *p* = 0.00390) but increased after 2014 (slope = 0.0418, *p* = 0.03895) (Figure 2).

### 3.5 Factors associated with willingness to use PrEP and dose–response meta‐analysis

**Figure 3:**
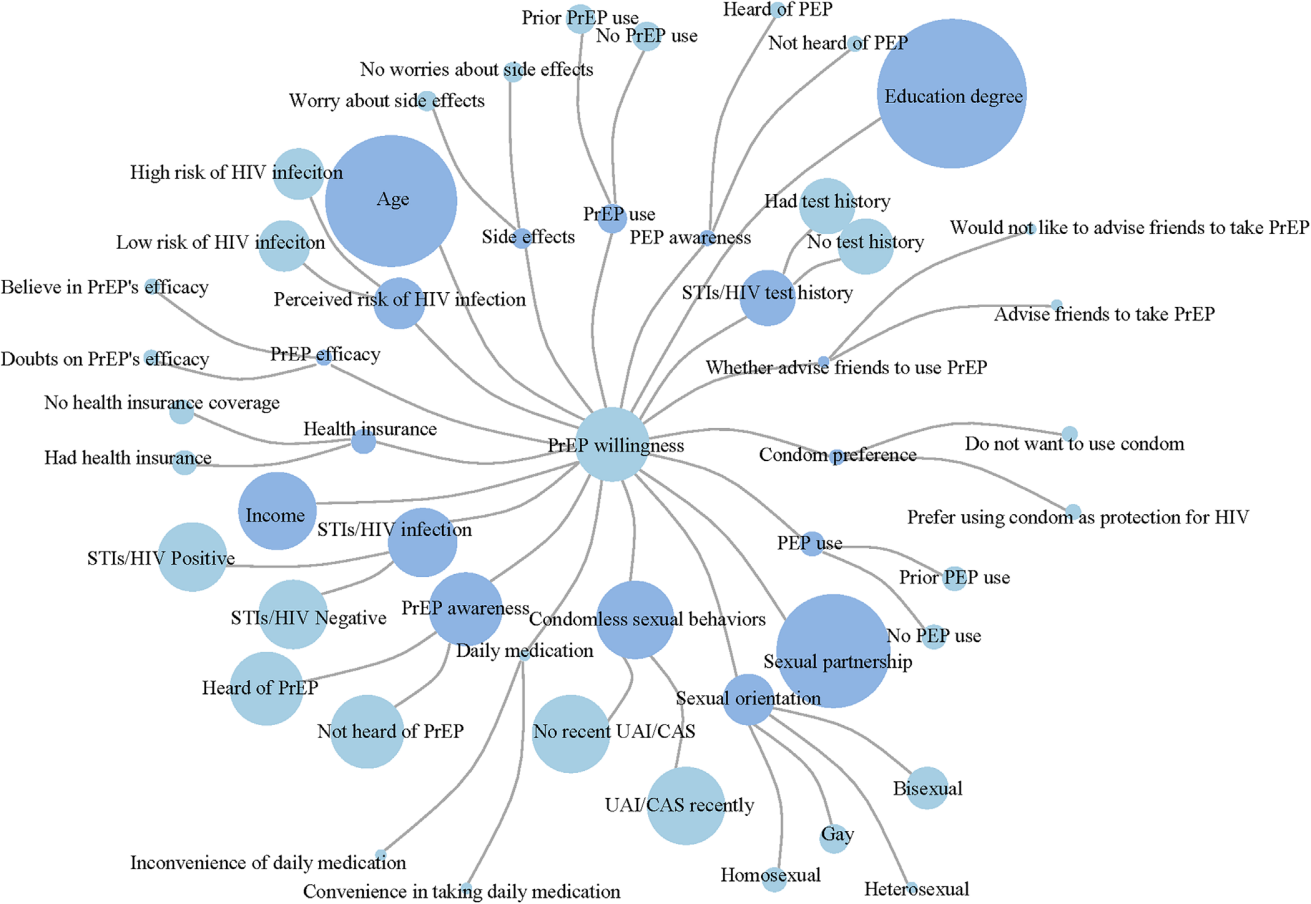
Factors associated with willingness to use PrEP. The larger size of the dot refers to being more frequently reported in the literature.

Among 156 studies, 73 studies provided a specific OR for factors associated with willingness to use PrEP. The main factors are illustrated in Figure 3, with the larger size of the dot referring to being more frequently reported in the literature. The main facilitators of willingness to use PrEP were PrEP awareness, recent condomless sexual behaviours, STI/HIV test history, STI/HIV positivity, perceived high risk of HIV infection, PrEP use and post exposure prophylaxis (PEP) use. MSM who would like to recommend friends to use PrEP were more willing to use PrEP. The main barriers were doubts about the efficacy of PrEP, worries about the side effects of PrEP and heterosexual orientation (Table A2 Factors associated with willingness to use PrEP among MSM in Appendix A).

**Figure 4:**
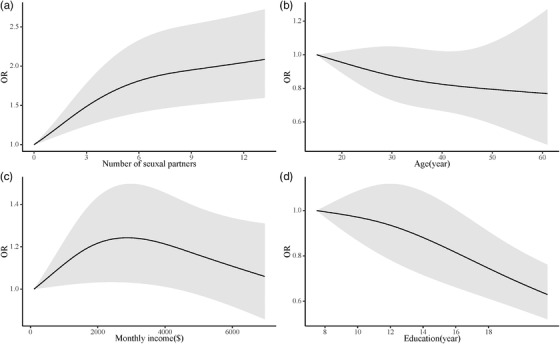
Dose–response meta‐analysis for the associations between selected factors and willingness to use PrEP. 95% confidence intervals are illustrated as the grey shade areas.

The DRMA results revealed that the fitting of the RCS model was statistically significant for willingness to use PrEP based on the number of sexual partners (*p*<0.0001) and years of education (*p*<0.0001) but not significant based on age (*p* = 0.2023) or monthly income (*p* = 0.0629). The Wald test results of the number of sexual partners (*p*<0.0001) and years of education (*p*<0.0001) demonstrated nonlinearity. The RCS results are illustrated in Figure 4, indicating that MSM were more willing to use PrEP when they had more sexual partners but less willing to use PrEP when they had more years of education.

### 3.6 Quality assessment

The quality assessment results of quantitative and qualitative studies are presented in Tables A3 and A4 in Appendix A, respectively. Of the 150 quantitative studies included, 142 studies were classified as weak and eight were classified as moderate according to the EPHPP tool. Most of the included studies were cross‐sectional, and the study design was not described as randomized or blinded, which affected the global rating results. Six qualitative studies were of moderate and robust quality according to the CASP tool.

### 3.7 Discussion

To the best of our knowledge, this meta‐analysis is the first to summarize trends in PrEP awareness, willingness to use PrEP, their correlation and the correlates of willingness to use PrEP among MSM based on global data. Our results showed that the proportion of MSM willing to use PrEP among global MSM was moderate, while the awareness of PrEP was low. Subgroup analyses showed that the awareness of PrEP varied among countries based on economic status and WHO regions, type of PrEP (formulation and route of administration), policies, publications and years in which research was conducted. PrEP willingness differed among countries with different economic status and study populations. The proportion of MSM aware of PrEP rose from 2007 to 2019, while the piecewise linear model showed that willingness to use PrEP decreased from 2007 to 2014 and then increased after 2014. No significant publication bias was found for PrEP awareness or willingness.

In 2012, WHO recommended PrEP for men and TG who have sex with men [6], and the U.S. Food and Drug Administration approved the use of PrEP in the same year [19]. Before 2015, PrEP was not available in many countries and unaccounted differences in study populations related to availability over time may contribute to the decreasing trend in willingness to use PrEP between 2007 and 2014. Later, in 2015 [7], WHO extended its recommendation to all “people at substantial risk of HIV infection” as a part of combination HIV prevention approaches, which may be the reason why the proportion of MSM willing to use PrEP increased after this time period. Accordingly, clinical practitioners could advise those key populations about PrEP, or prescribe PrEP according to these guidelines, thereby increasing the provision of information on PrEP to MSM and the rollout of PrEP, also in turn, likely increasing the trend in PrEP awareness and the willingness to use PrEP. Correspondingly, awareness was higher in countries that approved the use of PrEP for prevention than in countries that had not yet approved it. As we have shown, increased awareness of PrEP was associated with higher willingness to use PrEP, underscoring the benefits for policy makers to scale up the use of PrEP because of the increasing awareness of PrEP and willingness to use PrEP.

Individuals from HIC and countries that had approved PrEP reported higher PrEP awareness. Regulatory approval and implementation of PrEP in LMIC has been slow, which likely explains the low awareness in those settings [20, 21]. Healthcare services and comprehensive PrEP education were also more available in HIC [22], which could be associated with a higher awareness of PrEP than in LMIC. Although a previous meta‐analysis of 23 studies from LMIC conducted in 2017 found that PrEP awareness and willingness to use PrEP among MSM was 29.7% (95% CI: 16.9–44.3) and 64.4% (95% CI: 53.3–74.8), respectively [9], another meta‐analysis conducted in 2018 found that the overall acceptability of PrEP was 57.8%, and there was no difference between LMIC and HIC [10]. Consistent with the previous meta‐analysis conducted in 2018, our study showed that willingness to use PrEP was more common than awareness among MSM [9]. Although the willingness to use PrEP was comparable among different populations used to calculate the proportion of willingness in these studies, individuals showed higher willingness to use PrEP after they were informed about PrEP, which was supported by previous studies [9, 23]. However, willingness or intention are not necessarily the same with subsequent action [24], which could be affected by other various factors, such as behavioural skills [25]. Further real‐time studies are needed to summarize the proportion of actual PrEP use among MSM, correlating with awareness and intent.

Awareness of PrEP varied among different WHO regions, which might reflect different economic status and health policies. The lowest awareness was in South East Asia in the current study, where MSM had relatively higher burden of HIV highlighting the necessity and urgency to bring PrEP to scale as an essential component of comprehensive prevention strategies [26]. In addition, PrEP awareness campaigns should be expanded by the government and community partners to frame PrEP prevention messages appropriately based on accurate information and in easy‐to‐understand formats and ways [27, 28].

Individuals in both HIC and LMIC reported a higher willingness to use PrEP after adopting their PrEP guidelines. However, the pooled estimate on the willingness of individuals in LMIC was higher than that of HIC. In a meta‐analysis study by Peng et al. in 2018 [10], they reported a similar, albeit non‐significant, result. In HIC, people paid more attention to privacy because PrEP drugs were also used for HIV treatment, and high incidence populations preferred not to use PrEP due to stigma [29, 30].

Our study showed that willingness to use PrEP was higher among MSM in high incidence groups who were at risk of HIV than those not at risk of HIV, while the awareness of PrEP was comparable between the two groups. The higher willingness among groups at risk of HIV could be attributed to their perceived high risk of HIV infection [31]. Therefore, more efforts should be made to improve the awareness of PrEP, especially for MSM belonging to high HIV incidence groups who meet WHO criteria for PrEP use.

Our study identified several factors that could influence the willingness to use PrEP. Knowledge of PrEP has been found to be important in affecting willingness to use PrEP. MSM with prior knowledge of PrEP were more likely to accept PrEP and less worried about its efficacy. Individuals with a higher education level were not necessarily more likely to use PrEP, but were more likely to use condoms during intercourse [32] and expressed a higher degree of condom use self‐efficacy [33]. Individuals who preferred using condoms for protection against HIV and always used condoms were less willing to use PrEP [22, 31]. Another previous study also suggested that MSM with higher condom use self‐efficacy had decreased odds of entering PrEP contemplation [34]. In addition, individuals with a higher education level were more concerned about long‐term side effects or toxicity of PrEP according to one previous study [35], which may result in the lower willingness to use PrEP. Accordingly, robust awareness of PrEP, including its efficacy and side effects, helps to promote the willingness to take PrEP. These studies suggest that provision of comprehensive and accurate information about PrEP to MSM is essential for the future rollout of PrEP and to help to allay their concerns.

Our study also noted that MSM with condomless sexual behaviours or perceived high risk of HIV infection were more willing to use PrEP. Beyond that, MSM who had a history of an STI test and used PEP were more likely to accept it [36, 37, 38, 39, 40], likely reflecting their perception that they were at high risk of HIV infection and, therefore, more aware of HIV prevention approaches. Multiple sexual partners similarly determined willingness to use PrEP [41]. Furthermore, focusing on social networks of those who are likely to recommend PrEP to their friends is suggested as another effective strategy to promote PrEP uptake [42], as well as using peer educators to promote PrEP.

Some limitations in our study should be noted. First, substantial heterogeneity of study populations was observed in our systematic review. We conducted subgroup analyses to explore this finding. Age, sample size, publication year, research year, country, policy and study population were considered, but substantial heterogeneity remained in subgroups. Second, only a small number of studies were included in some subgroup analyses, such as awareness of different modalities of PrEP, which will warrant further investigation when more data become available since the type of PrEP may influence willingness to use it. Finally, most of the included studies were weak in quality assessment, which may weaken the strength of conclusions in the absence of higher quality research.

## 4 CONCLUSIONS

Our review demonstrated that the awareness of PrEP was a determinant of the willingness to use PrEP. The proportion of MSM willing to use PrEP was moderate, while the proportion of MSM aware of PrEP was low on the world scale. Both the awareness of PrEP and the willingness to use PrEP have increased in recent years, which indicated the positive effects of the introduction or scaling up of PrEP usage. With an increasing number of countries providing access to PrEP, improving awareness of PrEP through increasing access, expansion of health education for PrEP, enhancing risk perceptions of HIV infection and carrying out peer education could have positive effects on willingness to use PrEP among MSM.

## COMPETING INTERESTS

The authors declare that they have no competing interests.

## AUTHOR CONTRIBUTIONS

HJ designed the research study. ZS, QG, YD and JS contributed to acquisition of data. ZS and QC analysed and interpreted the data. ZS, QG and YD drafted the manuscript. HJ, HZ, BA, PL and YY revised the manuscript critically for important intellectual content. All the authors reviewed and approved the manuscript.

## Supporting information


**File S1**. PRISMA 2009 checklist

**File S2**. Detailed search strings

## Data Availability

All data extracted for this systematic review are contained in the manuscript and supporting information.

## APPENDIX A

### A.1

**Table A1:** Study characteristics

No.	Title	Author, publication year	Research year	Country or region	Study design	Sample size	Participants' characteristics	PrEP awareness	Willingness^a^ to use PrEP	Factors associated with willingness to use PrEP
1	Who would use PrEP? Factors associated with intention to use among MSM in London: a community survey	Adamma Aghaizu et al., 2012	2011	London, UK	Cross‐sectional study	768 MSM^1^	HIV‐negative participants had a mean age of 34.1 years, SD 9.2 years, range 18.5–71.5 and 18.0% (151/839) were of a non‐white ethnic background. Most respondents were employed (86%, 720/835) and had more than 2 years of education postage 16 (93%, 777/833)	Not reported	50.2% (386/768)	MSM were more likely to consider future PrEP use if they were <35 years (adjusted OR [AOR] 1.57, 95% CI 1.16–2.14), had unprotected anal intercourse with casual partners (AOR 1.70, 95% CI 1.13–2.56) and had previously used PEP (AOR 1.94, 95% CI 1.17–3.24)
2	Acceptability of oral preexposure prophylaxis among men who have sex with men in Philadelphia	Adams J. W. et al., 2016	2014	Philadelphia, USA	Cross‐sectional study	537 MSM	Self‐reported being HIV negative	Not reported	60% (324/537)	Unprotected anal sex
3	Awareness, willingness to use, and history of HIV PrEP use among gay, bisexual, and other men who have sex with men in Nigeria	Adedotun Ogunbajo et al., 2019	2019	Nigeria	Cross‐sectional study	413 MSM^2^	Age (mean = 29.2, SD = 5.8)	53.60%	80.10%	Having health insurance and history of PrEP use
4	Awareness and acceptability of pre‐exposure prophylaxis (PrEP) among gay, bisexual and other men who have sex with men (GBMSM) in Kenya	Adedotun Ogunbajo et al., 2019	2014	Kenya	Cross‐sectional study	459 MSM	Age range = 18–29 years (mean age = 22.5 years, SD = 3.2 years). 47.5% of the sample identified as gay/homosexual and 81.7% had completed secondary education or higher	64.30%	44.90%	For individual‐level variables in the PrEP acceptability model, increasing scores on self‐esteem (OR = 2.37; *p* = 0.002) and internalized homonegativity (OR = 1.26; *p* = 0.006) were associated with higher odds of PrEP acceptability.Increasing scores on condom use self‐efficacy (OR = 1.57; *p* = 0.005), depression/anxiety (OR = 1.63; *p* = 0.001) and PrEP self‐efficacy (OR = 6.12; *p* <0.001) were all associated with higher odds of PrEP acceptability
5	The role of sexual risk behaviors on PrEP awareness and interest among men who have sex with men in Latin America	Alberto Edeza et al., 2019	2012	Latin America (Mexico, Brazil and Colombia)	Cross‐sectional study	22,698 MSM^3^	The average age of respondents was 30.0 years (*n* = 22,698; SD = 8.8). Most participants identified themselves as middle class (*n* = 16,359; 74.8%), had completed a university or post‐graduate degree (*n* = 17,852; 79.0%), lived in urban areas (*n* = 21,707; 95.6%) and identified as homosexual/gay (*n* = 17,163; 75.8%)	10.4% (*n* = 2053)	62%	In adjusted models for the overall sample, MSM who received payment for sex had higher odds of expressing interest in participating in a PrEP trial (aOR = 1.45, 95% CI: 1.25–1.71) compared to MSM who did not receive payment for sex. Similarly, MSM who engaged in recent serodiscordant/serostatus unknown CAS had higher odds of expressing interest in participating in a PrEP trial (aOR = 1.74, 95% CI: 1.57–1.95)
6	Knowledge of pre‐exposure prophylaxis (PrEP) for HIV prevention among men who have sex with men in Denver, Colorado	Alia A. et al., 2014	2008–2011	Denver, USA	Cross‐sectional study	2008: 425 MSM 2011: 461 MSM	2008: Almost two‐thirds (66%) of participants were over the age of 30 years 2011: Almost two‐thirds (62%) of participants were over the age of 30 years	2008: 21% (*n* = 91) 2011: 28% (*n* = 131)	2008: 66%, 2011: 62%	Not reported
7	Awareness, willingness, and perceived efficacy of pre‐exposure prophylaxis among adolescent sexual minority males	Alvin Gordián‐Arroyo et al., 2020	2018	America	Cross‐sectional study	761 YMSM	Mean age = 16.22 years (SD = 1.36)	68.20%	90.8% (681/761)	Only race was found to be associated with willingness to use PrEP (*p* = 0.043)
8	Are Thai MSM willing to take PrEP for HIV prevention? An analysis of attitudes, preferences and acceptance	Ana Wheelock et al., 2013	2011	Bangkok and Chiang Mai, Thailand	Cross‐sectional study	260 MSM	Age = 16–18 years (4%, *n* = 11); 19–24 years (54%, *n* = 139); 25–30 years (27%, *n* = 71); 31–35 years (11%, *n* = 28); age≥36 years (4%, *n* = 11)	Not reported	Yes, definitely, 39.2%, yes, probably, 49.2%	Willingness to use PrEP remained high even after learning of potential mild side effects (24.6% ‘‘yes, definitely’’ and 56.5% ‘‘yes, probably’’), having to pay 500 Baht/month for it (58.8% ‘‘yes, definitely’’ and 35.8% ‘‘yes, probably’’). A minority of participants would feel embarrassed about taking PrEP (2.7% ‘‘very embarrassing’’ and 5.8% ‘‘fairly embarrassing’’)
9	Pre‐exposure prophylaxis (PrEP) dissemination: adapting diffusion theory to examine PrEP adoption	Ashley Schuyler et al., 2021	2016	Chicago, USA	Mixed method study	181 MSM	48% of the participants were aged 17–20 years and 52% were aged 21–24 years	Approximately 88% of participants (*n* = 160/181) reported having heard of PrEP	Not reported	Both social stigma and HIV health literacy were statistically significant, with those who were unaware of PrEP reporting higher levels of perceived social stigma (aOR: 2.05, *p*<0.03) and lower HIV health literacy (aOR: 0.74, *p*<0.05) than those who were PrEP aware PrEP adopters were more likely to report sexual risk (aOR: 2.92, *p*<0.03) and have higher levels of education (aOR: 2.40, *p*<0.05) and, unexpectedly, less likely to have health insurance (aOR: 0.24, *p*<0.03)
10	Awareness of and willingness to use pre‐exposure prophylaxis (PrEP) among people who inject drugs and men who have sex with men in India: results from a multi‐city cross‐sectional survey	Ashwin Belludi et al., 2021	2016–2017	India	Cross‐sectional study	8621 MSM	Median age = 28 years, site range 21–31	8.0% (690/8621) of MSM were aware of PrEP	67.6% (5278/8621)	Having a main male sexual partner and injection drug use in the prior 6 months, symptoms of a sexually transmitted infection in the prior 6 months, hazardous alcohol use, HIV testing in the prior 12 months and higher stigma scores were significantly associated with willingness to use PrEP
11	Awareness and willingness to use HIV pre‐exposure prophylaxis among men who have sex with men in Rwanda: a cross‐sectional descriptive survey	Athanase Munyaneza et al., 2021	September 2016 and February 2017	Rwandan	Cross‐sectional study	225 MSM	The mean age was 26.7 years (median age = 26 years; interquartile range was 23–29 years), of which 67 (30%) were 23 years old or younger	Of the 225 participants, 104 (48%) reported awareness of PrEP	Of the 225 participants, 181 (83%) reported that they were willing to take PrEP	The odds of awareness of PrEP were almost twice as high (OR 1.86, 95% CI [1.05–3.30]) for those having receptive anal sex with inconsistent condom use compared with those who did not have receptive anal sex and less likely for those who reported living with other (e.g. family or friends; OR 0.35, 95% CI [0.16–0.76]) compared with those living with male or female partners The likelihood of being willing to use PrEP was almost half as low among those who reported insertive anal sex acts and inconsistent condom use than those who did not have insertive anal sex (OR 0.45, 95% CI [0.21–0.97])
12	HIV pre‐exposure prophylaxis (PrEP) knowledge, attitudes and perceptions of sexual health risk in an age of STI antimicrobial resistance	Ava Lorenc et al., 2021	October 2018–November 2019	Bristol, UK	Mixed method study	617 MSM	198 survey respondents were aged <30 years (32%); 293 survey respondents were aged 30–49 years (48%); and 126 survey respondents were aged >50 years (20%)	202/578 (34.9%)	Most survey respondents (and interviewees) who had never used PrEP would take it if it was free of charge (256/376, 68%)	Among non‐users, 39% (146/376) were unaware how to access PrEP and 27% (103/376) could not access PrEP through the national “Impact” trial of whom 79% (81/103) were eligible. PrEP was described as “life‐changing,” but expense was the main barrier to use
13	Access to basic HIV‐related services and PrEP acceptability among men who have sex with men worldwide: barriers, facilitators, and implications for combination prevention	Ayala et al., 2013	2012	145 countries (including Africa, Asia, Europe and Latin America)	Cross‐sectional study	2774 MSM^4^	Between 12 and 90 years	69.80%	80.80%	Lower PrEP stigma, less knowledge about PrEP, country income level and demographic characteristics
14	Assessing the need for a pre‐exposure prophylaxis programme using the social media app Grindr	B. Hampel et al., 2017	2017	Switzerland	Cross‐sectional study	1893 MSM^5^	The median age of the participants was 35.9 years (first quartile [Q1] = 28; third quartile [Q3] = 43). The youngest participant was 18 years old, and the oldest was 74 years old	79.8% (*n* = 1510)	77.90%	Of those, participants in all age groups under 50 years old more often answered this question positively compared with those aged 50 years and older (<20 years: OR: 1.2; *p* = 0.02; 20–29 years: OR: 1.15; *p* = 0.001; 30–39 years: OR: 1.16; *p* < 0.001; 40–49 years: OR: 1.07; *p* = 0.1)
15	Predictors of interest in taking pre‐exposure prophylaxis among men who have sex with men who used a rapid HIV‐testing site in Montreal (Actuel sur Rue)	B. Lebouché et al., 2015	2012–2013	Montreal, Canada	Cross‐sectional study	1179 MSM	84% (*n* = 989) participants are homosexual, 11% (*n* = 132) are bisexual and 5% (*n* = 58) are heterosexual. 42% (*n* = 492) participants have college/university undergraduate education and 36% (m = 425) have graduate degree. 27% (*n* = 301) participants annual income less than $20,000 dollar	30%	55% (*n* = 653)	In the adjusted model, only being in a serodiscordant couple in the past 12 months, then number of sexual partners in the past 3 months and temporal events were significantly associated with interest in PrEP. MSM who reported being part of a serodiscordant couple were two and a half times more likely to be interested in PrEP than those who did not (adjusted odds ratio [aOR] 2.56; 95% CI 1.44–4.58; *p* = 0.001), while MSM with more than 10 partners, relative to those with fewer than five, were 73% more likely to be interested in PrEP (aOR 1.73; 95% CI 1.17–2.55; *p* = 0.006). Finally, odds of interest were 82% greater (*p* = 0.003) after there lease of Quebec's PrEP guidelines as compared with the period before 5 October 2012
16	Exploring patterns of awareness and use of HIV pre‐exposure prophylaxis among young men who have sex with men	Benjamin B. Strauss et al., 2017	2015	USA	Cross‐sectional study	759Y MSM	Mean age = 24.2 years	67.50%	Not reported	Barriers: being unaware of the availability of PrEP, conversely, believing the medication to be too expensive
17	Willingness to use HIV pre‐exposure prophylaxis among gay men, other men who have sex with men and transgender women in Myanmar	Bridget L. Draper et al., 2017	2014	Yangon and Mandalay, Myanmar	Cross‐sectional study	434 MSM	58% (*n* = 253) participants from Mandalay, 42% (*n* = 181) from Yangon. The median age is 23 (IQR: 20–28) years old	5% (*n* = 23)	62% (*n* = 270)	The association between never or only occasionally using condoms with casual partners and willingness to use PrEP was marginally significant (aOR = 2.02; 95% CI = 1.00–4.10). GMT who reported concern about side effects and long‐term use of PrEP were less likely (aOR = 0.35; 95% CI = 0.21–0.59) to be willing to use PrEP
18	Willingness to take, use of, and indications for pre‐exposure prophylaxis among men who have sex with men – 20 U.S. cities, 2014	Brooke E. Hoots et al., 2016	2014	U.S. cities	Cross‐sectional study	6483 MSM^6^	22.5% (*n* = 1458) participants were black, 30.0% (*n* = 1812) were Hispanic/Latino, 41.0% (*n* = 2660) were white. 75.0% (*n* = 4864) participants education was more than high school. 27.9% (*n* = 1809) participants annual income was less than $ 20,000	Not reported	61% (*n* = 3940)	Other race were more willing to take PrEP (aPR: 1.1, 95% CI: 1.0–1.2). Younger MSM were more willing to take PrEP. Those who reported a bacterial STD in the past 12 months were more likely to be willing to take PrEP than those who did not (aPR: 1.2, 95% CI: 1.2–1.3). MSM who reported casual male sex partners in the past 12 months were more likely to be willing to take PrEP compared to those who reported only main partners (aPR: 1.3, 95 CI%: 1.2–1.4)
19	Acceptability of pre‐exposure prophylaxis for HIV prevention: facilitators, barriers and impact on sexual risk behaviors among men who have sex with men in Benin	Carin Ahouada et al., 2020	2018	Benin	Cross‐sectional study	400 MSM	Mean age = 26.2 ± 5.0 years	50.7% (*n* = 203)	35.8% (143/400)	The facilitators associated with PrEP acceptability were: not having to pay for PrEP (odds ratio [OR] = 2.39, 95% CI: 1.50–4.46) and its accessibility within MSM networks (OR = 9.82, 95% CI: 3.5–27.52). Only one barrier was significant: the concern that taking PrEP be perceived as a marker of adopting HIV risky behaviours (OR = 0.11, 95% CI: 0.04–0.30)
20	Acceptability of pre‐exposure prophylaxis (PrEP) among men who have sex with men (MSM) in Benin: a qualitative study	Carin Ahouada et al., 2019	2014	Benin	Qualitative study	30 MSM	Mean age = 27.1 years (SD: 5 years), age range = 19–37 years; most of the participants attained higher education (63.0% [*n* = 19] attended University)	43.30%	100%	Facilitators of PrEP use were: availability of medication, safety, absence of constraints as well as freedom to have multiple sex partners and sex with HIV‐positive friends. Barriers were: complex procedures for obtaining medication, size and taste of medication, cost of medication, poor PrEP awareness, disagreement of the partner, non‐receptive attitude of the MSM community towards PrEP and discrimination in health centres
21	Awareness, knowledge, use, willingness to use and need of pre‐exposure prophylaxis (PrEP) during World Gay Pride 2017	Carlos Iniesta et al., 2018	2011–2014	Spain	Cross‐sectional study	472 MSM	Mean age = 38 years, 77% (*n* = 107) had a university education and 85% (*n* = 403) were living in Spain, mostly in big cities	33%	67%	Willingness to use PrEP was high among HIV‐negative participants: 63% of them (*n* = 212) would use PrEP, mainly to prevent HIV (53%), to have unprotected sexual intercourse (14%) or to feel safer during sex (8%). The main reasons to reject PrEP (*n* = 112) were condom preference (22%), lack of prevention of other STIs (19%) or having a steady partner (13%). Only two participants reported not wanting to use PrEP for economic reasons. In the multivariable analyses, meeting PrEP criteria (aOR = 2.67) and not having a university education (aOR = 0.52) were independently associated with using or willingness to use PrEP
22	Engagement with peer health educators is associated with willingness to use pre‐exposure prophylaxis among male sex workers in Ho Chi Minh City, Vietnam	Catherine E. Oldenburg et al., 2014	2010	Ho Chi Minh City, Vietnam	Cross‐sectional study	300 MSM	27% were aged 15–19 years	Not reported	95.40%	Side effects
23	Challenges in translating PrEP interest into uptake in an observational study of young black MSM	Charlotte‐Paige Rolle et al., 2017	2015	Atlanta, America	Cohort study	184 YBMSM^7^	Median age = 24 years (IQR: 22–26 years); 70% (*n* = 129) reported at least some college education; 59% (*n* = 108) had health insurance; and 78% (*n* = 143) self‐identified as gay or homosexual	53% (*n* = 97)	63%	The only factor associated with PrEP initiation was reported STI in the prior year (PR 1.50, 95% CI 1.002–2.25)
24	Willingness to use pre‐exposure prophylaxis among Black and White men who have sex with men in Atlanta, Georgia	Charlotte‐Paige Rolle et al., 2018	2012–2013	Atlanta, USA	Cross‐sectional study	482 MSM^8^	The median age of the sample was 26 years (IQR 22, 31), including 219 (45.4%) black and 263 (54.6%) white MSM. Most had at least some college education (84%, 406/482) and health insurance coverage (67%, 321/482)	Not reported	45%	In multivariable analysis, reported UAI in the last 12 months was the only variable significantly associated with PrEP willingness (OR 1.73, 95% CI 1.13–2.65) Willing men identified “extra protection” against HIV as the most common reason for interest in using PrEP, whereas unwilling men most commonly cited not wanting to take medication daily. Most men indicated willingness to use PrEP if cost was <50 dollars/month; however, more black MSM indicated willingness to use PrEP only if cost were free (17.9% of white MSM vs. 25.9% of black MSM, *p* = 0.03)
25	Willingness to take PrEP and potential for risk compensation among highly sexually active gay and bisexual men	Christian Grov et al., 2015	2011–2013	New York, USA	Cohort study	206 MSM^9^	Average age was 34 (SD = 11.8). 13.6% (*n* = 28) sample was black, 11.2% (*n* = 23) was Latino and 60.2% (*n* = 124) was white. 44.2% (*n* = 91) participants income was less than $30,000	Not reported	46.10%	Chi‐square test, 4‐year college education (χ^2^ = 5.80, *p* = 0.02), recency of last HIV test (χ^2^ = 7.09, *p* = 0.03), hypersexual disorder screening inventory (HDSI) diagnosis (χ^2^ = 6.16, *p* = 0.01) were related to willingness to take PrEP
26	Changes in familiarity with and willingness to take preexposure prophylaxis in a longitudinal study of highly sexually active gay and bisexual men	Christian Grov et al., 2016	2011–2014	New York, America	Cohort study	158 MSM^10^	15.2% (*n* = 24) were black, 12.0% (*n* = 19) were Latino, 58.9% (*n* = 93) were white; 83.5% (*n* = 132) were gay, 16.5% (*n* = 26) were bisexual; 39.2% (*n* = 62) had a bachelor's degree; and 31.6% (*n* = 50) had a graduate degree	At baseline, 22.8% (*n* = 36) were familiar with PrEP. After 12 months of being enrolled in the study, 32.3% (*n* = 51) were familiar with PrEP	At baseline, 46.8% (*n* = 74) expressed willingness to take PrEP. After 12 months of being enrolled in the study, 44.9% (*n* = 71) expressed willingness to take PrEP	Homosexuals are more likely to use PrEP than bisexuals (AOR: 2.53; 95% CI: 1.11–5.74; *p*≤0.05). Men reported at least a college degree are less likely to use PrEP than those with less education (AOR: 0.40; 95% CI: 0.21–0.77; *p*≤0.01). Reporting SC symptomology (AOR: 1.86;95% CI: 1.04–3.32;*p*≤0.05) and any condomless anal sex (AOR: 2.21; 95% CI: 1.25–3.91; *p*≤0.01) was associated with higher odds of willingness to take PrEP
27	2016 PREP attitudes in Germany: high awareness and acceptance in MSM at risk of HIV	Christoph D. Spinner et al., 2018	2016	Germany	Cross‐sectional study	866 MSM^11^	Mean age = 37.0 ± 10.4 years; 593 participants (68.5%) were tested for HIV within the past 12 months	86.4% (*n* = 748)	564 (65.1%)	Risk behaviour was significantly associated with higher PrEP acceptance (OR 2.90, 95% CI 2.14–3.90), as was a history of STDs (OR 1.85, 95% CI 1.17–2.91). A history of HIV testing was found to be associated with PrEP acceptance in univariate analysis only (*p* = 0.038) and was also included in the model
28	Pre‐exposure prophylaxis: awareness, acceptability and risk compensation behaviour among men who have sex with men and the transgender population	CK Uthappa et al., 2017	2015	India	Cross‐sectional study	400 MSM	The mean age of participants was 27 years with a standard deviation (SD) of 6.7 years, with almost 88% of respondents being ≤ 35 years. The majority of them were single (60%), had received some schooling (86%) and were employed (77%)	7% (*n* = 28)	99% (*n* = 396)	Facilitators: education, being married or in a live‐in relationship, having a high calculated risk and having a high self‐assessed risk. The barriers: increasing age and higher income.
29	Characterizing biomedical HIV prevention awareness and use among black transgender women in the United States	Cristian J. Chandler et al., 2021	2014–2017	America	Cross‐sectional study	490 BTW	Mean age = 31 years	55.5% (273/490)	Not reported	Not reported
30	Acceptability of pre‐exposure prophylaxis among men who have sex with men and transgender women in Northern Thailand	Daniel Yang et al., 2013	2012	Chiang Mai, Thailand	Cross‐sectional study	131 MSM, 107 TG	MSM ages ranged from 18 to 49 years with a mean age of 23.7 years. TG ages ranged from 18 to 33 years with a mean age of 21.8 years. 38% (*n* = 50) of MSM and 62% (*n* = 66) of TG were enrolled in or had completed a bachelor's degree or higher	66% (TG) 66% (MSM)	41% (MSM) 37% (TG)	In bivariate analysis of MSM participants, PrEP acceptability was associated with having zero regular partners in the preceding 6 months (OR 2.25, *p* = 0.04) versus one or more partners, regularly planned sex (OR 2.83, *p* = 0.01) versus unplanned sex, infrequent sex (once per month or less, OR 2.36, *p* = 0.02) versus two or more sexual encounters per month, a lifetime history of STIs (OR 3.78, *p*<0.01) versus no history of STIs, a lifetime history of HIV testing (OR 1.95, *p* = 0.07) versus no history of HIV testing, age 25 years or older (OR 2.30, *p* = 0.02) versus age less than 25 years and being “very confident” in the ability to take daily, oral medicines for 1 year (OR 2.63, *p* = 0.01) versus not being “very confident.” In contrast, receptive anal sex positioning (OR 0.47, *p* = 0.08) was negatively associated with PrEP acceptability versus insertive or versatile positioning
31	Monitoring HIV preexposure prophylaxis use among men who have sex with men in Washington State: findings from an internet‐based survey	Darcy White Rao et al., 2019	2017	Washington State, USA	Cross‐sectional study	1080 MSM^12^	The median age in the sample was 30 years	78.90%	36%	Current PrEP use was associated with older age, higher education and meeting indications for PrEP use
32	What if my dad finds out!?: assessing adolescent men who have sex with men's perceptions about parents as barriers to PrEP uptake	David A. Moskowitz et al., 2020	July 2018–February 2019	USA	Mixed method study	491 AMSM^13^	Participants ranged in age from 13 to 18 years old with the majority (50.5%) reporting 16 or 17 years old	55.2% (*n* = 271)	37.9% (185/488) of MSMs would definitely take PrEP if it were free; 29.9% (146/488) of MSMs would probably take PrEP if it were free; 23.2% (113/488) of MSMs reported that they might take PrEP if it were free	The most endorsed reason participants did not currently use PrEP was concern that their parents might find out (32.2%). While the other reasons were as followed: I don't know enough about it (20.6%); I think I am at no or low risk for HIV (17.7%); I do not want to go to the doctor and get blood work every 3 months (17.5%); Not sexually active (15.3%); I cannot afford it and/or I do not have insurance (9.6%); I do not want to take a pill everyday (7.7%); I think condoms are a better choice than PrEP (6.5%); People who use PrEP are perceived negative by others (4.3%); I am in a serious relationship (4.3%)
33	Pre‐exposure prophylaxis (PREP) awareness and acceptability among men who have sex with men in Taiwan	Deng‐Min Chuang et al., 2018	2014	Taiwan, China	Cross‐sectional study	176 MSM^14^	Participants’ (*n* = 176) mean age was 27.4 years with a range of 18–50 years. The majority of participants self‐identified as gay (87.5%), had college or postgraduate degrees (65.9%), were employed full‐time (56.3%), had a monthly income below 30,000 TWD (60.2%) and were single (58.0%)	47.2% (*n* = 83)	72.2% (*n* = 127)	In multivariable analysis, PrEP acceptability was modelled as a function of sexual orientation, condomless anal sex, vicarious stigma and anticipated disclosure of PrEP use to sexual partners (*p* < 0.25). Participants who had higher vicarious stigma (AOR = 2.29, 95% CI [1.25, 4.19], *p* < 0.01) and who anticipated PrEP disclosure to sexual partners (AOR = 6.00, 95% CI [2.61, 13.82], *p* < 0.001) had higher odds of PrEP acceptability
34	Adherence to pre‐exposure prophylaxis among men who have sex with men: a prospective cohort study	Dou Qu et al., 2018	Not reported	Chongqing, Guangxi, Xinjiang and Sichuan, China	Cohort study	331 MSM^15^	Median age = 28 years, age range = 18–58 years; most participants lived in an urban household 74.02% (*n* = 245), were of Han nationality 90.61% (*n* = 300), had a college degree or above 63.33% (*n* = 209), were employed 77.13% (*n* = 253) and unmarried 79.46% (*n* = 263), and reported an income below 3000 RMB per month 50.30% (*n* = 164)	Not reported	32.33%	The main objective reasons for non‐adherence were “forgetting to take medicine” (70.21%), “too busy” (29.08%), “worrying about side effects” (28.01%) and “too much trouble” (18.44%)
35	Limited awareness and low immediate uptake of pre‐exposure prophylaxis among men who have sex with men using an internet social networking site	Douglas S. Krakower et al., 2012	2010–2011	USA	Cross‐sectional study	Pre‐iPrEx, 398 MSM;post‐iPrEx, 4558 MSM	Pre‐iPrEx participants mean age is 40.2 (SD = 12.1) Post‐iPrEx participants mean age is 39.0 (SD = 12.8)	(12.5% (36/289) pre‐iPrEx versus 19.0% (642/3387) post‐iPrEx)	(76.1% (220/289) pre‐iPrEx versus 78.5% (2654/3382) post‐iPrEx)	Being older, having greater self‐perceived risk of HIV acquisition and UAI with at least one male partner in the prior 3 months, awareness of PEP
36	Sociocultural influences on attitudes towards pre‐exposure prophylaxis (PrEP), history of PrEP use, and future PrEP use in HIV‐vulnerable cisgender men who have sex with men across the U.S.	Drew A. Westmoreland et al., 2021	2017–2018	USA	Cohort study	5817 MSM	Half (50.5%) of our participants were between 25 and 35 years old, with nearly one‐quarter (23.5%) being 16–24 years old. Most 85.4% of all participants identified as gay, queer or homosexual	Not reported	53.3% (*n* = 3100) reported that they had intentions to start using PrEP	Participants who knew between one and four people on PrEP (aOR: 1.21, 95% CI: 1.05–1.39 vs. knowing no one on PrEP) and participants who knew five or more people on PrEP (aOR = 1.27, 95% CI: 1.09–1.49) had significantly higher odds of reporting intentions to start PrEP. Additionally, participants who had networks that were opposed to PrEP (aOR = 0.45, 95% CI: 0.23–0.88 vs. in favour of PrEP), had split opinions about PrEP (equally in favour and opposed; aOR = 0.38, 95% CI: 0.32–0.45),
										did not have strong opinions (aOR = 0.37, 95% CI: 0.31–0.44) or did not know what PrEP was (aOR = 0.49, 95% CI: 0.41–0.58) had lower odds of reporting intentions to start PrEP. Demographic factors associated with increased odds of intending to start PrEP included younger age, being a racial or ethnic group other than white, reporting an annual income between $20,000 and $49,999, having reported housing instability in the past 5 years and reporting previous experience with PrEP. Demographic factors associated with lower odds of intending to start PrEP were older age and higher education status
37	Masculine ideology and Black men who have sex with men's interest in HIV pre‐exposure prophylaxis (PrEP)	Driver R. et al., 2020	2017	America	Cross‐sectional study	123 black men who have sex with men (BMSM)^16^	Mean age = 30.9 years (SD = 9.88)	88% (108/123)	65% (80/123)	Interest in PrEP demonstrated marginally significant relations to heterosexual self‐presentation, PrEP stigma and frequency of HIV testing. Avoidance of femininity was not correlated with PrEP interest
38	Attitudes and acceptance of oral and parenteral HIV preexposure prophylaxis among potential user groups: a multinational study	Eisingerich et al., 2012	2010.10–2011.5	Peru, India and South Africa	Mixed method study	383 MSM^17^	Mean age of MSM not reported; 39% were aged 16–24 and 6% were aged≥41 years	Not reported	61% ‘‘yes, definitely’’ and 30% ‘‘yes, probably’’	38% reported that PrEP would be “very embarrassing” to take
39	Qualitative assessment of readiness for use of HIV pre‐exposure prophylaxis among men who have sex with men (MSM) in Malawi: qualitative study using key informant interviews and focus group discussions	Elizabeth Mpunga et al., 2020	2018	Malawi	Qualitative study	109 MSM^18^	Most of the study participants were aged 35–40 years (27.5%) and the least were aged 30–34 years (12.8%)	40 of the 96 (41.7%) FGD participants indicated that they heard about prep before the study; 9 (69.2%) of the IDI participants had heard about PrEP prior the study	64 out of the 96 (66.7%) FGD study participants would use PrEP right away; five (38.5%) indicated that they would take PrEP immediately when it is rolled out	Participants highlighted barriers that would hinder them from taking PrEP, such as side effects, which were cited in IDIs and FGDs. Key factors from FGDs include cost, fear of being outed, drug stockouts, fear of being known as MSMs by wives and lack of relevant information. FGDs cited stigma from healthcare workers, forgetfulness and community‐associated factors
40	Awareness and use of HIV pre‐exposure prophylaxis among attendees of a Seattle Gay Pride Event and sexually transmitted disease clinic	Elizabeth Anne Barash et al., 2010	2009	King County, Washington	Cross‐sectional study	215 MSM^19^	61% were white, median age was 36.0 (standard deviation [SD] 12.3), 53% had a college degree, 60% made $30,000 or more per year and 67% had health insurance	22% (*n* = 48)	94 (44%)	Annual income less than $15,000 (OR: 2.14; 95%CI: 1.06–4.35), being <40 years old (OR: 1.97; 95%CI: 1.06–3.66) and recruited from the STD clinic (vs. from the Gay Pride event) (OR: 1.49;95% CI: 0.82–2.71) were significantly associated with participants' great interest in taking PrEP
41	Perceptions among Dutch men who have sex with men and their willingness to use rectal microbicides and oral pre‐exposure prophylaxis to reduce HIV risk – a preliminary study	Elske Marra et al., 2015	Not reported	Dutch	Mixed method study	108 MSM	Mean age: 35 years	Not reported	28.90%	Potential adverse events, the feeling of being a patient
42	Gearing up for PrEP in the Middle East and North Africa: an initial look at willingness to take PrEP among young men who have sex with men in Beirut, Lebanon	Erik D. Storholm et al., 2019	2016–2017	Beirut, Lebanon	Cohort study	218 MSM^20^	Most (61.5%) were under age 25	42.7% (*n* = 93)	55.5% (*n* = 121)	Knowledge of HIV risk, awareness of PrEP, having had recent condomless anal sex
43	Disparities in the PrEP continuum for trans women compared to MSM in San Francisco, California: results from population‐based cross‐sectional behavioural surveillance studies	Erin C. Wilson et al., 2020	Trans*National Study: 2016–2018 National HIV Behavioral Surveillance: 2017	San Francisco, USA	Cross‐sectional study	399 MSM 369 TW	MSM: Median age = 36 years (IQR: 29–49) TW: Median age = 37 years (IQR: 27–51)	Of overall sample MSM, 96.74% (386/399) were aware of PrEP; of overall sample TW, 79.13% (292/369) were aware of PrEP	Not reported	Not reported
44	Improved PrEP awareness and use among trans women in San Francisco, California	Erin C. Wilson et al., 2021	July 2019–February 2020	San Francisco, USA	Cross‐sectional study	116 TW	The mean age was 47.7 years (median 43, interquartile range [IQR] 32–52)	94.0% (109/116) participants had heard of PrEP	Not reported	Increased awareness of PrEP was associated with seeing PrEP advertisements (OR 4.64, 95% CI 1.26–17.16, *p* = 0.021) and hormone use (OR 7.98, 95% CI 1.46–43.59, *p* = 0.017). Lower odds were found for those sero‐positive for HCV antibodies (OR 0.19, 95% CI 0.039–0.97, *p* = 0.046)
45	PrEP awareness in the context of HIV/AIDS conspiracy beliefs among Black/African American and Hispanic/Latino MSM in three urban US cities	Evelyn Olansky et al., 2019	2014	USA	Cross‐sectional study	836 MSM	358 (43%) participants aged between 18 and 29 years, 203 (24%) participants aged between 30 and 39 years, 275 (33%) participants aged above 40 years; 34% (*n* = 286) were HIV positive and 66% (*n* = 550) were HIV negative	47% (*n* = 395)	Not reported	Men reporting HIV/AIDS conspiracy beliefs were less likely to be aware of PrEP (aOR = 0.52, 95% CI = 0.38–0.71). HIV‐positive men were more likely to report PrEP awareness than HIV‐negative men (aOR = 3.83, 95% CI = 2.74–5.38) and having a college degree or more (vs. some college or less) was associated with PrEP awareness (aOR = 1.62, 95% CI = 1.10–2.38)
46	Factors associated with PrEP refusal among transgender women in Northeastern Brazil	Fabiane Soares et al., 2019	2014–2016	Northeastern Brazil	Cross‐sectional study	127 TGW	Most of the participants were less than 25 years old (57%)	Not reported	91.30%	URAI with casual partners were more likely to refuse PrEP
47	Willingness to accept HIV pre‐exposure prophylaxis among Chinese men who have sex with men	Feng Zhou et al., 2012	2009–2010	Beijing, China	Cohort study	152 MSM^21^	Age of the participants ranged from 18 to 61 years, with a mean age of 29.768.6 years	11.20%	67.80%	In the multivariate logistic regression model, those who did not have consistent condom use in homosexual behaviour in the past 6 months (OR: 0.31; 95% CI: 0.13–0.70) and had never heard of the side effects of ARV drugs (OR: 0.30; 95% CI: 0.14–0.67) were not willing to accept PrEP
48	Knowledge, attitudes and practices regarding pre‐exposure prophylaxis (PrEP) in a sample of Italian men who have SEX with MEN (MSM)	Gianluca Voglino et al., 2021	Not reported	Italy	Cross‐sectional study	196 MSM	The median age was 31 years old. The vast majority (97.4%) was Italian and most of the participants (68.9%) had a university degree and worked as employees (35.2%)	Most of the participants (91.1%, 174/191) had heard of PrEP before, 87.2% (150/172) of them knew what PrEP was	More than half of the participants stated that they would be more willing to use PrEP if they had more information about it (52.1%, 99/190), if it were free (66.5%, 127/191) or if it were purchasable without medical prescription (57.4%, 109/190)	The results from the regression showed that being regularly tested for HIV is the strongest factor associated with PrEP knowledge (OR = 3.09; CI = 1.15–8.34), even when adjusting for the other variables included in the analysis (adjusted odds ratio (aOR = 3.16; CI = 1.06–9.29). Other variables associated with PrEP knowledge were being single (OR = 2.96; CI = 1.14–6.01) and having had sex with more than one man in the last 12 months (OR = 3.94; CI = 1.48–6.89), but these results were not statistically significant when the model was adjusted for the other included variables
49	Association of HIV pre‐exposure prophylaxis awareness, preferred Spanish (vs. English) language use, and sociodemographic variables among Hispanic/Latino men who have sex with men	Gordon Mansergh et al., 2018	2010–2014	Chicago, Fort Lauderdale and Kansas City, America	Cross‐sectional study	927 MSM^22^	49% (*n* = 457) were black, 51% (*n* = 470) were Hispanic/Latino; 42% (*n* = 392) aged 18–29 years; 52% (*n* = 480) reported condomless anal sex in the past 3 months	247/484 (51%)	Not reported	In bivariate analysis, black men were more likely than Hispanic/Latino men to intend to use PrEP (81% vs. 70%, *p* 0.05)
50	“Belt and braces approach; added benefit and … extra reassurance”: a multi‐stakeholder examination of the challenges to effective provision of pre‐exposure prophylaxis (PrEP) for HIV prevention among men who have sex with men (MSM) in Northern and Central England	Hillis A. et al., 2021	2018–2019	UK	Qualitative study	20 MSM	Range 24–59 years old	40% (8/20)	Not reported	Not reported
51	Awareness and willingness to use pre‐exposure prophylaxis (PrEP) among men who have sex with men and transgender women in Brazil	Hoagland et al., 2016	2014.4–2015.7	Brazil	Cross‐sectional study	1131 MSM	Median age = 29 years. 46.8% were HIV positive	61.30%	82.10%	Aware of PrEP, more years of schooling, recent STD diagnosis
52	Awareness and acceptance of HIV pre‐exposure prophylaxis among medical personnel and men who have sex with men in Korea	Hyun‐Ha Chang et al., 2018	2016–2017	Korea	Cross‐sectional study	266 MSM (2016) 123 MSM (2017)	Among the MSM surveyed in 2016, 55.3% (*n* = 147) were HIV infected and 44.7% (*n* = 119) were non‐HIV infected; the median age of HIV‐infected MSM was 38.0 years (IQR: 28.0–43.5 years) and the median age of non‐HIV‐infected MSM was 32.0 years (IQR: 27.0–38.0 years); 77.4% (*n* = 206) had a college degree or higher. Among the MSM surveyed in 2017, 50.4% (*n* = 62) were HIV infected and 49.6% (*n* = 61) were non‐HIV infected; the median age of HIV‐infected MSM was 36.5 years (IQR: 29.0–41.0 years) and the median age of non‐HIV‐infected MSM was 33.0 years (IQR: 27.0–40.0 years); 68.3% (*n* = 84) had a college degree or higher	61.3% (2016), 88.6% (2017)	43.2% (2016) 65.0% (2017)	Barriers to PrEP use mainly included: lack of insurance coverage in MSM (80.8%, 2016; 94.3%, 2017), lack of knowledge about efficacy (45.5%, 2016; 39.0%, 2017), worry of adverse effects (53.0%, 2016; 61.8%, 2017), worry of acquisition of resistance (51.5%, 2016; 37.4%, 2017), exposure to other people when candidates get prescription (42.5%, 2016; 53.7%, 2017)
53	Facilitators and barriers to pre‐exposure prophylaxis willingness among young men who have sex with men who use geosocial networking applications in California	Ian W. Holloway et al., 2017	2015	California, USA	Cross‐sectional study	687 YMSM^23^	About 43% of participants were from the greater Los Angeles area, 25% were from the Bay Area and the remaining 32% were from other regions in California. Mean age was 23 years. In terms of race/ethnicity, 33% were Latino, 25% were black, 21% were white and 21% were other/mixed. Most identified as male (97%), had sex with men exclusively (82%) and identified as gay (80%). Smaller percentages identified as bisexual (17%) or another sexual orientation (3%). Forty percent worked full time, while 23% worked part time and 25% were students. Most completed high school (93%) and over half indicated an annual income <$30,000 (63%). Three‐quarters indicated being insured (74%) and nearly all were U.S. citizens (90%)	74% (*n* = 508)	55% (*n* = 380)	Hispanic/Latino YMSM were more likely than white YMSM to be willing to take PrEP (odds ratio [OR]: 1.73; confidence interval [CI]: 1.01–2.98; *p* = 0.046). Compared to YMSM reporting low concern for getting HIV, those with medium (OR: 1.87; CI: 1.14–3.07; *p* = 0.014) and high concern (OR: 1.84; CI: 1.13–3.01; *p* = 0.015)were nearly twice as likely to be willing to take PrEP. Greater concerns about PrEP drug effects were associated with decreased odds of being willing to take it (OR: 0.46; CI: 0.33–0.65; *p*<0.001). Increased medical mistrust was associated with decreased willingness to take PrEP (OR: 0.71; CI: 0.53–0.96; *p* = 0.026). Finally, greater concerns regarding ability to adhere to PrEP were associated with decreased willingness (OR: 0.65; CI: 0.49–0.88; *p*<0.005). Higher scores in perceived benefits of PrEP were associated with higher odds of willingness (OR: 2.59; CI: 1.78–3.78)
54	Awareness and willingness to use HIV pre‐exposure prophylaxis amongst gay and bisexual men in Scotland: implications for biomedical HIV prevention	Ingrid Young et al., 2013	2011	Scotland	Cross‐sectional study	1393 MSM^24^	Mean age = 32.8 years (age range = 18–83 years, SD = 10.88 years)	31.2% (*n* = 434)	54.3% (*n* = 756)	The adjusted odds of being likely to use PrEP on a daily basis were higher for men who had secondary (OR: 1.63; 95%CI: 1.17–2.26; *p* = 0.004) or further/vocational‐level education (OR: 1.75; 95% CI: 1.38–2.22; *p*<0.001) compared to degree or postgraduate‐level education, visited the gay scene at least 2–3 times a month (OR: 1.40; 95% CI: 1.05–1.86; *p* = 0.022) or more (OR: 1.55; 95% CI: 1.21–2.00; *p* = 0.001) compared to once a month or less, reported any higher risk UAI (OR: 2.24; 95% CI: 1.77–2.83; *p*<0.001), had an HIV or STI test in the previous 12 months (OR: 1.53; 95% CI: 1.23–1.90; *p*<0.001) and had heard of PrEP (OR: 1.30; 95% CI: 1.04–1.64; *p* = 0.024); the odds remained lower for men who were aged 26+ compared to men aged 18–25 and men who were uncertain or not optimistic about HIV treatment (OR: 0.55; 95%CI: 0.42–0.73; *p*<0.001)
55	Anti‐retroviral therapy based HIV prevention among a sample of men who have sex with men in Cape Town, South Africa: use of post‐exposure prophylaxis and knowledge on pre‐exposure prophylaxis	J.M. Hugo et al., 2016	2014	Cape Town, South Africa	Cross‐sectional study	40 MSM^25^	Mean age = 35.87 years (SD: 11.14 years), 80% were under the age of 40, with the youngest 20 and the eldest 65 years old; 5 participants did not identify as gay, 80% (*n* = 32) of participants were employed and 66.7% (*n* = 26) had some form of university education or more; 70% (*n* = 28) reported their monthly income as greater than $1000 (USD)	90% (*n* = 36)	75% (*n* = 30)	Barriers to considering PrEP use were being concerned about side effect (5.0%), cost (5.0%), potential resistance (5.0%) and inconvenience of daily pill (10.0%). Of the 30 participants who indicated that they would use PrEP, 27 (90%) would use it even if it caused mild temporary side effects and 25 (83.3%) would use it even if they still had to use condoms (100%). All of them would use PrEP if they had to take it daily and 23 participants (76.7%) indicated that they would use PrEP even if they had to pay for it. 28 participants (93.3%) indicated that they would use PrEP even if they had to have regular HIV tests
56	Pre‐exposure prophylaxis knowledge and use among men who have sex with men in a small metropolitan region of the Southeastern United States	James A. Griffin et al., 2020	2016	America	Cross‐sectional study	164 MSM	Mean age = 36.14 years (SD = 13.92)	80.5% (132/164)	Not reported	Chi‐square analyses revealed that non‐white MSM were more than three times as likely to have used PrEP than white MSM (17.4 vs. 4.7%; x^2^ = 5.77; *p*<0.05). Those who were tested for HIV in the prior year were more likely to have used PrEP (14.3% vs. 0%; x^2^ = 7.39; *p*<0.01). Those who were tested for other STIs in the prior year were more likely to have used PrEP (17.7% vs. 1.4%; x^2^ = 10.42; *p*<0.01)
57	Use of an HIV‐risk screening tool to identify optimal candidates for PrEP scale‐up among men who have sex with men in Toronto, Canada: disconnect between objective and subjective HIV risk	James Wilton et al., 2015	2014–2015	Toronto, Canada	Cross‐sectional study	420 MSM	Median age was 31 years	72%	52.50%	Moderate‐to‐high perceived HIV risk
58	Who will use pre‐exposure prophylaxis (PrEP) and why?: understanding PrEP awareness and acceptability amongst men who have sex with men in the UK – a mixed methods study	Jamie Frankis et al., 2016	2012–2013	UK	Mixed method study	690 MSM^26^	Mean age = 37 years (range 18–84 years, SD = 12.9 years), 97.7% were white and 82.3% were gay identified, 16.8% were bisexual (16.8%) and 0.9% identified as straight	29.7% (*n* = 205)	47.8% (*n* = 330)	When controlling for the factors significant at the bivariate level in the multivariate logistic regression, the adjusted odds of the likelihood of PrEP use remained significantly higher for men aged 18–25 compared to men in the three older age groups (25–35 AOR = 0.61, 95%CI: 0.4–0.95; 36–45 AOR = 0.47, 95% CI: 0.30–0.75; 46+ AOR = 0.54, 95% CI: 0.35–0.82) and for men who reported any higher risk UAI in the previous 12 months (AOR = 2.27, 95% CI: 1.37–3.78)
59	Towards preparedness for PrEP: PrEP awareness and acceptability among MSM at high risk of HIV transmission who use sociosexual media in four Celtic nations: Scotland, Wales, Northern Ireland and The Republic of Ireland: an online survey	Jamie S. Frankis et al., 2015	2012–2013	Scotland, Wales, Northern Ireland and The Republic of Ireland	Cross‐sectional study	386 MSM^27^	Mean age = 37 years (SD = 12.9 years, age range = 18–82 years); 98.2% (*n* = 379) were white	34.50%	63.5% (226/356)	In the multivariable model, PrEP acceptability was only associated with reporting ≥5 CAI partners (OR 2.04, 95% CI 1.2–3.46) in the last year
60	What do Dutch MSM think of preexposure prophylaxis to prevent HIV‐infection? A cross‐sectional study	Janneke P. Bil et al., 2015	2012–2013	Amsterdam, Netherlands	Cross‐sectional study	448 MSM	Median age was 40 years (interquartile range [IQR] 35–45), most were Dutch (92%; 404/440), college graduates (90%; 404/447) and had a medium income (41%; 163/397)	54% (*n* = 242)	13% (*n* = 58)	High‐risk MSM were more likely to have a medium (adjusted odds ratio [aOR]: 1.78 [95% confidence interval [CI] 1.07–2.97]) or high (aOR: 3.92 [95% CI 1.68–9.15]) intention to use PrEP than low‐risk MSM, as were MSM with higher perceptions of self‐efficacy to use PrEP (high intention: aOR: 6.15 [95% CI 2.50–15.09]) and higher perceptions of relief due to PrEP (medium intention: aOR: 2.67 [95% CI 1.32–5.40]; high intention: aOR: 14.87 [95% CI 5.98–37.01]) than MSM with lower perceptions. MSM with higher perceptions of shame about using PrEP (medium intention: aOR: 0.35 [95% CI 0.19–0.62]; high intention: aOR: 0.22 [95% CI 0.07–0.71]) or with more worries about side effects were less likely to have a high (aOR: 0.18 [95% CI 0.06–0.54]) or medium (aOR: 0.29 [95% CI 0.12–0.72]) intention to use PrEP
61	Trends in the awareness, acceptability, and usage of HIV pre‐exposure prophylaxis among at‐risk men who have sex with men in Toronto	Jayoti Rana et al., 2018	2013–2016	Toronto, Canada	Cross‐sectional study	Survey wave 1^28^ April–June 2013 (436 MSM) survey wave 2 May–August 2014 (400 MSM) survey wave 3 November 2014–April 2015 (420 MSM) survey wave 4 May–August 2016 (400 MSM)	Median (interquartile range, IQR) age was 31.0 (26, 38) years; (59.5%, 54.8%, 55.2% and 54.0%, respectively, in four time periods) were white; (67.8%, 72.0%, 69.7% and 71.0%, respectively, in four time periods) reported being employed full time	Awareness of PrEP increased significantly over the four time periods from 26.7% to 58.3% to 71.8% to 91.3%	Willingness to use PrEP increased slightly over the four time periods from 51.0% to 51.6% to 52.5% to 56.5%	The only variables associated with willingness to use PrEP in adjusted analyses were moderate‐to‐high perceived HIV risk (aOR = 2.44, 95% CI = 1.82–3.27) and HIRI‐MSM score (aOR = 1.61 per 10‐point increase, 95% CI = 1.40–1.86); prior use of PEP had an aOR = 1.50 (95% CI = 0.998–2.250)
62	Awareness of, interest in, and willingness to pay for HIV pre‐exposure prophylaxis among Canadian gay, bisexual, and other men who have sex with men	Jeffrey Morgan et al., 2018	2017	Canada	Cross‐sectional study	7176 MSM^29^	Median age = 42 years (IQR: 29–53 years)	54.7% (*n* = 3923)	72.10%	In both univariable and multivariable models, interest in using PrEP was positively associated with respondents who self‐reported non‐white (AOR: 1.31; 95% CI: 1.14–1.51) and non‐Aboriginal (AOR: 1.09; 95% CI: 0.87–1.37) ethnicity (compared to white). Conversely, interest was negatively associated with self‐identifying as bisexual (AOR: 0.80; 95% CI: 0.72–0.90) and “other” (AOR: 0.56; 95% CI: 0.47–0.67) (compared to self‐identifying as gay), being ≥50 years old (AOR: 0.80; 95% CI: 0.70–0.92) (compared to ≤30) and holding a graduate degree (AOR: 0.70; 95% CI: 0.52–0.95) (compared to some high school)
63	Familiarity with and preferences for oral and long‐acting injectable HIV pre‐exposure prophylaxis (PrEP) in a national sample of gay and bisexual men in the U.S.	Jeffrey T. Parsons et al., 2016	2014	USA	Cross‐sectional study	948 MSM^30^	The average age in the sample was 40 and ranged from 18 to 79	10.1% (*n* = 96)	46.00%	Education and age
64	Uptake of HIV pre‐exposure prophylaxis (PrEP) in a national cohort of gay and bisexual men in the United States	Jeffrey T. Parsons et al., 2017	2015	USA	Cohort study	995 GBM^31^	The majority was gay identified (95%) and had a 4‐year college degree (58.7%). Nearly half (47.1%) made $50,000 per year or more. The average age was 41.9 years (median = 40.0, SD = 13.9)	Not reported	Of those who met the criteria for “objective identification” as PrEP candidate (*n* = 636), 65.9% (419/636) indicated willingness to take PrEP	Not reported
65	Factors associated with awareness and use of pre‐exposure prophylaxis (PrEP) among Black men who have sex with men with a recent STI diagnosis	Jessica L. Maksut et al., 2020	December 2012–October 2015	USA	Cross‐sectional study	209 MSM^32^	Men who identified as gay/same gender loving comprised 62.2% (*n* = 130) of the sample, while the remaining participants identified as bisexual (31.4%, *n* = 65) or heterosexual (5.8%, *n* = 12). The average age of participants was 31.6 (SD = 11.1, range = 19–73)	The majority of participants (*n* = 152, 73.4%) reported that they were aware of PrEP for HIV prevention	Not reported	In the multivariable model with PrEP awareness as dependent variable, age, education and HIV risk perceptions remained significant. PrEP aware persons were significantly younger in age (OR = 0.97, 95% CI: 0.94–0.99, *p* = 0.030) and had significantly higher educational attainment (OR = 1.89, 95% CI: 1.22–2.94, *p* = 0.027). In addition, participants who were PrEP aware had significantly higher levels of HIV risk perceptions (OR = 1.30, 95% CI: 1.08–1.58, *p* = 0.019) than PrEP unaware participants
66	A tale of two cities: exploring the role of race/ethnicity and geographic setting on PrEP use among adolescent cisgender MSM	Jessica Londeree Saleska et al., 2020	May 2017–September 2019	New Orleans and Los Angeles, USA	Cross‐sectional study	729 MSM	Of 729 adolescents who identified as cisMSM, approximately 23% (*n* = 166) were white, 46% (*n* = 334) were AA and 31% (*n* = 229) were Latinx. Over half (*n* = 406, 56%) of the participants lived in Los Angeles, while 44% (*n* = 323) lived in New Orleans	Most (*n* = 600, 82%) reported that they had heard of PrEP in their lifetime	Not reported	In New Orleans, approximately 79% (*n* = 255) of adolescents had heard of PrEP and awareness was highest among white adolescents (87%), followed by AA (77%) and Latinx (75%) adolescents. PrEP awareness was somewhat higher in Los Angeles, where approximately 85% (*n* = 345) of adolescents had heard of PrEP. As in New Orleans, PrEP awareness in Los Angeles was highest among white adolescents (90%), followed by Latinx (87%) and AA (77%) adolescents
67	Acceptability of oral versus rectal HIV preexposure prophylaxis among men who have sex with men and transgender women in Peru	Jesus Peinado et al., 2013	2008	Lima, Iquitos and Pucallpa, Peru	Cross‐sectional study	532 MSM	Median age = 28 years (IQR: 23–35 years), age range = 18–68 years	Not reported	96.20%	After adjustment for age, city and education, only being receptive most of the time (aOR: 9.1, 95% CI: 1.8–46.5, *p* = 0.01) and exclusively receptive (aOR: 7.5, 95% CI: 1.6–53.2, *p* = 0.01) during anal intercourse, compared to being versatile, were independently associated with acceptability for using oral PrEP products. A similar association was found with the acceptability of a rectal formulation (i.e. lube; aOR: 2.3, 95% CI: 0.9–6.1, *p* = 0.07; and aOR: 2.5, 95% CI: 1.1–5.4, *p* = 0.02)
68	Propaganda methods of pre‐exposure prophylactic medication in men who have sex with men: a multiple correspondence analysis	Jiatong He et al., 2014	Not reported	China	Cross‐sectional study	1323 MSM^33^	Mean age = 26.97 ± 7.56 years	31.40%	Not reported	Not reported
69	PrEP uptake preferences among men who have sex with men in China: results from a National Internet Survey	Jing Han et al., 2019	2017	China	Cross‐sectional study	4581 MSM	54.6% (*n* = 2501) of the participants were 18–25 years old; 78.6% (*n* = 3603) were single (78.6%); 68.6% (*n* = 3141) had attended some form of college or university; 69.2% (*n* = 3170) identified themselves as homosexual; 38.6% (*n* = 1768) reported earning less than 10,000 RMB per year	22.40%	26.0% said “definitely yes,” 49.6% were “probably yes”	Participants who had heard of PrEP were more likely to say “definitely yes” (AOR = 1.7, 95% CI: 1.4–2.2) compared to those who had never heard of PrEP. Those who reported UAI in the last 12 months had a tendency to say “definitely yes” (AOR = 1.4, 95% CI: 1.1–1.5) compared to those who did not report UAI. Compared to those who had HIV testing in the past year, participants who had never tested for HIV were less likely to say “definitely yes” to PrEP uptake (AOR = 0.7, 95% CI: 0.5–0.8). Participants who had a low self‐perceived risk of HIV were less likely to say “definitely yes” (AOR = 0.3, 95% CI: 0.2–0.4). A similar trend was found among those who preferred condoms as their primary form of HIV prevention (AOR = 0.5, 95% CI: 0.4–0.5). Those who had concerns about financial burdens and access to PrEP were more likely to say “definitely yes” to the PrEP uptake compared to those who did not voice those concerns (AOR = 1.4, 95% CI: 1.1–1.6). In the adjusted model, being over 40 years old was associated with PrEP uptake (AOR = 2.0, 95% CI: 1.1–3.6)
70	An integrated examination of county‐ and individual‐level factors in relation to HIV pre‐exposure prophylaxis awareness, willingness to use, and uptake among men who have sex with men in the US	Jingjing Li et al., 2018	2014–2015	USA	Cross‐sectional study	8338 MSM	The sample was 75.2% white	63.3% (*n* = 5281)	2804/4372 (64.1%)	Younger age, receipt of HIV behavioural interventions, having an STI, having more than one male sex partner (vs. having one), having condomless anal sex with an HIV positive/unknown status male partner
71	A survey on HIV/AIDS‐related knowledge, attitudes, risk behaviors, and characteristics of men who have sex with men among university students in Guangxi, China	Jingzhen Lai et al., 2020	2016–2017	China	Cross‐sectional study	49 MSM	8 MSM age ≤20 years, 41 MSM age >20 years	26.53% (*n* = 13)	71.43% (35/49)	The Zhuang ethnic minority was found as the independent promoting factor of PrEP acceptance. Living in Nanning for more than 2 years, thinking MSM is infectious, alcohol consumption < 250 ml/day and the experience in PrEP implementation were found to be independent impeditive factors to the acceptance of PrEP
72	Knowledge and attitudes about preexposure prophylaxis (PrEP) among sexually active men who have sex with men and who participate in New York City Gay Pride Events	Joanne E. Mantell et al., 2014	2011	New York, USA	Cross‐sectional study	477 MSM^34^	438 participants were HIV negative (91.3%)	38.8% (*n* = 185)	79.40%	Perceived themselves at high risk for HIV, side effects
73	Congruence between hypothetical willingness to use pre‐exposure prophylaxis (PrEP) and eligibility: an online survey among Belgian men having sex with men	Johannes Bullinger et al., 2019	2016–2017	Belgian	Cross‐sectional study	1444 MSM	Median age was 36.5 years	91.8% (*n* = 1326)	69.5% (*n* = 1004)	Higher PrEP awareness (*p* <0.001), better PrEP knowledge (*p* < 0.001), more risky sexual behaviour (i.e. CAI) (*p* < 0.001)
74	Willingness to use pre‐exposure prophylaxis (PrEP) for HIV prevention and PrEP implementation preferences among transgender women in Malaysia	Jonathan M. Galka et al., 2020	2017	Malaysia	Cross‐sectional study	361 TGW	Mean age = 35.3 years (standard deviation [SD] = 9.8)	20.2% (73/361)	82.3% (297/361)	Cost (62.9%) was the most reported concern, followed by general side effects (41.8%), efficacy (31.0%), safety (28.5%), convenience of acquiring PrEP (15.5%) and fear that PrEP may interact with gender‐affirming hormones (13.6%)
75	Pre‐exposure prophylaxis (PrEP) awareness among black men who have sex with men with a history of criminal justice involvement in six U.S. cities: findings from the HPTN 061 study	Jonathan P. Feelemyer et al., 2021	2009–2010	America	Cross‐sectional study	914 BMSM^35^	Median age = 39 years	7.9% (15/914)	Not reported	Not reported
76	Interest in long‐acting injectable PrEP in a cohort of men who have sex with men in China	Kathrine Meyers et al., 2017	2007–2012	China	Cohort study	200 MSM^36^	Mean age was 31.6 years	33.5% (*n* = 67)	76% (152/200)	Higher education, having a female partner, having an HIV‐infected steady partner
77	Perspectives on and preferences for on‐demand and long‐acting PrEP among sexual and gender minority adolescents assigned male at birth	Kathryn Macapagal et al., 2020	November 2018–February 2019	USA	Mixed method study	59 MSM	Participants (*N* = 59) were between ages 14 and 18, with a mean age of 16.42 years (SD = 0.88). Most participants identified as gay (*n* = 43; 72.9%) and 94.9% of participants were cisgender male (*n* = 56)	49 participants had heard of PrEP before the study (83.1%)	Among post‐focus group participants (*N* = 53), 60.4% (*n* = 32) were likely/very likely to use daily PrEP pill and 52.8% (*n* = 28) were likely/very likely to use PrEP on demand	Perceived barriers to taking PrEP: not knowing where to get PrEP was ranked by most youth as a “very important” barrier (55.9%), followed by not being able to afford it (42.4%) and believing people who work at the clinic/doctor's office are not friendly to LGBTQ teens (35.6%)
78	PrEP awareness, uptake, barriers, and correlates among adolescents assigned male at birth who have sex with males in the U.S.	Kathryn Macapagal et al., 2019	2018.2–2018.4	USA	Cross‐sectional study	219 MSM	Participants ranged in age from 15 to 17 years (M age = 16.38, SD = 0.74)	54.8% (*n* = 120)	Not reported	Concerns about side effects, lower perceived risk of HIV, lower HIV knowledge, never having heard of PrEP
79	Risk perception and interest in HIV pre‐exposure prophylaxis among men who have sex with men with rectal gonorrhea and chlamydia infection	Katie B. Biello et al., 2018	2014–2016	America	Cross‐sectional study	401 MSM^37^	Mean age = 30.9 years (SD: 11.5 years); 89% (*n* = 356) reported having sex with other men only (MSM) and 11% (*n* = 45) reported having sex with both men and women (MSMW); 64% (*n* = 258) were white; 55% (*n* = 220) reported having health insurance; 87% (*n* = 349) reported having ever been tested for HIV	64% (*n* = 257)	96% (*n* = 376)	The most common reasons for reporting not being interested in using PrEP included concerns about medication side effects (*n* = 265, 66%), cost (*n* = 231, 58%), potential interaction with alcohol or drugs (*n* = 109, 27%), taking a medication every day (*n* = 101, 25%) and potential interaction with other medications (*n* = 67, 17%). In bivariate analyses (data not shown), patients who perceived themselves to be at high or medium risk for HIV were significantly more likely to express interest in using PrEP (odds ratio [OR] 1.88, 95% confidence interval [CI] 1.13–3.11; *p* = 0.014)
80	Pre‐exposure prophylaxis awareness, use, and intention to use in a regional sample of Latin American geosocial networking application users in 2018–2019	Kevin J. Blair et al., 2021	2018–2019	Latin America	Cross‐sectional study	718 MSM	18–25 years: 26.3% (*n* = 189), 26–30 years: 20.3% (*n* = 146), 31–40 years: 29.7% (*n* = 213), 41+ years: 23.7% (*n* = 170)	72.1% (518/718)	32.1% (157/718)	PrEP eligible respondents had increased odds of both current use of (aOR 13.84 [95% CI 5.65–33.91]) and intention to use PrEP in the next 6 months (aOR 2.26 [95% CI 1.26–4.07]). Recent STI testing was associated with greater intention to use PrEP (aOR 1.58 [95% CI 1.01–2.48])
81	Awareness of pre‐exposure prophylaxis for HIV, willingness to use it and potential barriers or facilitators to uptake among men who have sex with men in Spain	L. Ferrer et al., 2016	2015	Spain	Cross‐sectional study	866 MSM^38^	Mean age = 34.2 years (SD: 11.8 years) (*N* = 841)	28.7% (*n* = 247)	57.60%	74% of participants would be willing to use PrEP if it were a monthly injection, 70.2% if it was a pill taken before sex and 75% if PrEP was free or partly free of charge; 65% of participants reported that they would be willing to use PrEP at times of high risk and 57.7% if it was at least 80% effective
82	Knowledge and willingness to use pre‐exposure prophylaxis among men who have sex with men in Northeastern Brazil	Laio Magno et al., 2019	2016	Northeastern Brazil	Cross‐sectional study	32 MSM	Average age was 26	31.3% (*n* = 10)	15 (46.9%)	PrEP's efficacy and perceived side effects, low self‐perceived HIV acquisition risk, fear of disclosure of participants’ homosexual orientation, fear of being perceived as HIV positive, fear of discrimination and fear of being perceived as sexually promiscuous
83	Awareness and willingness to use HIV pre‐exposure prophylaxis (PrEP) among trans women in China: a community‐based survey	Li Yan et al., 2020	July 2018–May 2019	Nanjing and Suzhou, China	Cross‐sectional study	222 TW	Among 222 HIV negative or unknown serostatus trans women interviewed, 48.2% were youth age 18–24 years	33.3% (*n* = 74) were aware of PrEP	49.1% (*n* = 109) were willing to use PrEP	In multivariable analysis, PrEP awareness was positively associated with having a university degree or above (adjusted odds ratio [AOR] 2.77, 95% CI 1.31–5.89) and not using alcohol before or during sex (AOR 2.02, 95% CI 1.00–4.09). Those having one (AOR 3.56, 95% CI 1.68–7.54) or multiple sexual partners (AOR 2.53, 95% CI 1.24–5.15) were more likely to be willing to use PrEP compared to those with no partners
84	Willingness to use and adhere to HIV pre‐exposure prophylaxis (PrEP) among men who have sex with men (MSM) in China	Liping Peng et al., 2019	2018–2019	Chengdu, China	Cross‐sectional study	524 MSM	The mean age was 27.65 (±8.1). The majority of participants were of Han ethnicity (96.6%), were unmarried (91.0%), had sexual orientation as homosexual (77.11%), had obtained bachelor degree or above (61.5%) and had full‐time jobs (64.5%)	71.60%	84.90%	Adjusted for two background variables (sexual orientation and age), two variables were significantly and positively associated with the willingness to use PrEP: having more than five non‐regular partners in the past 6 months (AOR = 3.36; 95% CI: 1.10–10.26) and possessing a higher literacy in HIV prevention (AOR = 1.49; 95% CI: 1.03–2.16). Consistent condom use during sexual intercourse with regular partners (AOR = 0.47; 95% CI: 0.23–0.95) was significantly and negatively associated with the willingness to use PrEP. Drinking (AOR = 1.56; 95% CI: 0.95–2.56) and having heard of PrEP (AOR = 1.63; 95% CI: 0.99–2.68) were marginally and positively associated with the willingness to use PrEP, while having first homosexual intercourse at an older age (AOR = 0.47; 95% CI: 0.21–1.07, reference group: age under 18 years old) was marginally and negatively associated with willingness
85	Minimal awareness and stalled uptake of pre‐exposure prophylaxis (PrEP) among at risk, HIV‐negative, black men who have sex with men	Lisa A. Eaton et al., 2015	2012–2014	Atlanta, USA	Cross‐sectional study	436 MSM	Age M = 35.62, SD = 11.88	22% (*n* = 97)	81%	Not reported
86	A multi‐US city assessment of awareness and uptake of pre‐exposure prophylaxis (PrEP) for HIV prevention among black men and transgender women who have sex with men	Lisa A. Eaton et al., 2017	Not reported	USA	Cross‐sectional study	1274 MSM	Average age was 30.34 years (SD = 10.05)	38.6% (492)	Not reported	Testing for HIV in the past 6 months, having others being aware of sexuality and a greater number of female sex partners, education and condom use were negatively associated with PrEP uptake
87	Psychosocial factors related to willingness to use pre‐exposure prophylaxis for HIV prevention among Black men who have sex with men attending a community event	Lisa A. Eaton et al., 2014	2012	South‐eastern United States	Cross‐sectional study	398 MSM^39^	Mean age = 35 years; 96% were gay or bisexual	27.60%	60.30%	In the multivariate model, talk with provider about having sex with men in the past 6 months (OR: 1.85; 95% CI:1.17–2.94; *p*<0.01) and race‐based medical mistrust (OR: 0.72; 95% CI: 0.52–0.99, *p*<0.05) were significantly associated with PrEP acceptability
88	Stigma and conspiracy beliefs related to pre‐exposure prophylaxis (PrEP) and interest in using PrEP among Black and White men and transgender women who have sex with men	Lisa A. Eaton et al., 2017	2015	South‐eastern United States	Cross‐sectional study	85 BMTW, 179 WMTW	43.5% (*n* = 37) of BMTW and 60.3% (*n* = 108) of WMTW reported an annual income of more than 30,000$; 98.8% (*n* = 84) of BMTW and 97.2% (*n* = 174) of WMTW identified as male	BMTW: 54.1% (*n* = 46);WMTW: 68.2% (*n* = 122)	BMTW: 52.9% (*n* = 45); WMTW: 39.1% (*n* = 70)	For the multivariable model among BMTW, believing that PrEP was for people who were promiscuous remained the only variable associated with PrEP interest (OR = 0.51;95%CI: 0.30–0.87, *p*<0.01). In the multivariable model for WMTW, not being in a relationship (OR: 2.72;95% CI: 1.20–6.17, *p*<0.05) and being in a non‐monogamous relationship (OR: 4.03;95% CI: 1.11–14.66, *p*<0.05) versus being in a monogamous relationship, not currently having health insurance and having ever heard of PrEP (OR: 4.36; 95% CI: 1.75–10.34, *p*<0.01) were associated with interest in PrEP
89	Pre‐exposure prophylaxis among men who have sex with men in the Amsterdam Cohort Studies: use, eligibility, and intention to use	Liza Coyer et al., 2018	2015–2017	Amsterdam, the Netherlands	Cohort study	687 MSM	Median age was 40 (IQR 33–47) years in 2015	Not reported	Of 548 MSM with data on intention to use PrEP, 165 (30% [95% CI 26–34%]) reported a high intention, 277 (51% [95% CI 46–55%]) a medium intention	The proportion with a high intention was greater among eligible compared to non‐eligible MSM (51% [95% CI 43–59%] vs. 24% [95% CI 20–29%], *p*<0.001) and also greater among MSM who initiated PrEP between 2015 and 2017 compared to MSM who did not (80% vs. 26%, *p*<0.001). A high intention for daily use was reported by 96 (17%) MSM and for event‐driven use by 114 (21%) MSM
90	A qualitative assessment in acceptability and barriers to use pre‐exposure prophylaxis (PrEP) among men who have sex with men: implications for service delivery in Vietnam	Long Hoang Nguyen et al., 2021	2018	Vietnam	Qualitative study	30 MSM	Mean age = 23.9 years	23.3% (7/30)	Not reported	Regime, side effects, stigma, perceived low risk of HIV and cost hinder the acceptance of MSM to PrEP
91	Correlates associated with willingness to start pre‐exposure prophylaxis among young Black men who have sex with men (MSM) in Jackson, Mississippi	Lori M. Wardet, 2019	2018	Jackson, Mississippi	Cross‐sectional study	225 YBMSM	54.2% aged≤22 years; 64.0% had an education beyond high school; 21.8% had a history of incarceration; 30.2% attended religious service weekly	Not reported	45.30%^a^	For the total sample, participants who consistently used condoms for both insertive and receptive sex had a higher odds of being willing to start PrEP (odds ratio = 2.22; 95% confidence interval = 1.07–4.58) than participants who did not consistently use condoms for both insertive and receptive sex. Among the younger participants (aged 22 years or younger), those who had attained a level of education beyond high school had a higher odds of being willing to start PrEP (odds ratio = 2.42; 95% confidence interval = 1.06–5.55) relative to participants who had less than a high school education. For the older participants (aged 23 years or older), no significant associations were found between any of the demographic and sexual behaviour characteristics and willingness to start PrEP
92	PrEP interest and HIV‐1 incidence among MSM and transgender women in coastal Kenya	Makobu Kimani et al., 2017	2016–2017	Kenya	Cohort study	42 MSME, 112 MSMW, 14 TGW	Mean age = 26.7 years (IQR: 25.9–27.5 years); 68.5% had primary education only, 83.3% were single and 45.8% were Muslim	Not reported	MSM: 97.4% (149/153) TG: 7.1% (1/14)	Barriers to PrEP uptake considered specific to MSM subcategories included: HIV‐related stigma, daily dosing regimen, fear for side effects and concomitant drug use
93	Acceptability of pre‐exposure HIV prophylaxis clinical trial among MSM in Shenyang city	Mao et al., 2017	November 2015–May 2016	Shenyang, China	Cross‐sectional study	292 MSM	71.2% aged ≥25 years, median age = 29 years, 55.5% (162/292) had a secondary school degree or below, 27.7% (81/292) reported a monthly income of less than 2000 yuan, 55.5% (162/292) were single	34.20%	58.20%	Factors independently associated with those “on‐demand” would include: having more than two male sexual partners during the past 6 months (aOR = 1.7, 95% CI: 1.1–2.7), concerning on positive effect of PrEP (vs. side effects) (aOR = 6.4, 95% CI: 2.2–18.9), having HIV‐infected sexual partners (aOR = 8.1, 95%CI: 1.0–63.3) and self‐reported high risk for HIV (aOR = 2.6, 95% CI: 1.2–6.0)
94	Awareness and acceptability of pre‐exposure prophylaxis (PrEP) among men who have sex with men in Kazakhstan: a mixed methods study	Marieke Bak et al., 2018	2017	Kazakhstan	Mixed method study	108 MSM	18–25 years: 35.9% (*n* = 38), 26–35 years: 46.2% (*n* = 49), 36–45 years: 17% (*n* = 18), ≥46 years: 0.9% (*n* = 1); 13% (*n* = 14) of the participants had finished primary or secondary education and 87% (*n* = 94) had finished higher education	39.8% (*n* = 43)	85.2% (*n* = 92)	Those already aware of PrEP were more likely (OR = 5.63, 95% CI 1.21–26.19, *p* = 0.028) to be accepting of PrEP. Reasons for not being interested in PrEP included: current methods of preventing against STIs were effective enough (68.8%), worried about side effects (75.0%), would not want to take daily medication (75.0%), do not want to undergo regular medical check‐ups (25.0%), fear to be seen in a negative light (12.5%), did not want to pay for it (50.0%) and use condoms less often (12.5%)
95	PrEP interest among men who have sex with men in the Netherlands: covariates and differences across samples	Mart van Dijk et al., 2020	2016	Netherlands	Cross‐sectional study	426 MSM	Mean age was 42 years	89.9% (*n* = 383)	28.20%	Having correct prior PrEP knowledge, not having used a condom for last sex, having ever used drugs in a sexual context
96	HIV preexposure prophylaxis cascades to assess implementation in Australia: results from repeated, National Behavioral Surveillance of Gay and Bisexual Men, 2014–2018	Martin Holt et al., 2020	GCPS: 2014–2018 PrEPARE: 2017	Australia	Cross‐sectional study	GCPS: 39,670 MSM PrEPARE: 1038 MSM	GCPS: mean age = 35.2 years, 70.7% were born in Australia, 51.4% had a university degree and 64.3% were in full‐time employment PrEPARE: mean age = 36.3 years, 80.3% were born in Australia, 45.5% had a university degree and 64.5% were in full‐time employment	GCPS: 2014 8.3% (*n* = 563) 2015 11.5% (*n* = 845) 2016 15.6% (*n* = 1406) 2017 28.8% (*n* = 2491) 2018 32.4% (*n* = 2555) PrEPARE: 52.7% (*n* = 547)	GCPS: not reported PrEPARE: 35.6% (*n* = 370)	Focusing on the multivariate analysis, willingness to use PrEP was less likely among men aged 40 years and older and more likely among men who had CAI with casual partners and those who were recently tested for HIV. Willingness to use PrEP was not independently related to the other variables, although we note that being born in Australia and knowing other people using PrEP were associated with greater willingness to use PrEP at a bivariate level
97	Willingness to use and have sex with men taking HIV pre‐exposure prophylaxis (PrEP): results of online surveys of Australian gay and bisexual men, 2011–2015	Martin Holt et al., 2017	2011–2015	Australia	Cross‐sectional study	2011: 1161 MSM^40^ 2013: 1233 MSM 2015: 1145 MSM	The mean age of all respondents was 32.4 years (SD = 11.3) and 93.5% identified as gay. The majority were born in Australia (79.9%), lived in the capital city of their state or territory (73.6%), were in full‐time employment (58.1%) and almost half had a university degree (47.1%)	Not reported	28.2% in 2011, 23.3% in 2013 to 31.7% in 2015	Willingness to use PrEP was positively associated with having an HIV‐positive regular partner (AOR = 2.68, 95% CI 1.35–5.30, *p* = 0.005), CAIC in the previous 6 months (AOR = 1.72, 95% CI 1.07–2.76, *p* = 0.03), more than 10 male sex partners in the previous 6 months (AOR = 2.37, 95% CI 1.34–4.17, *p* = 0.003) and ever having taken PEP (1.46, 95% CI 1.01–2.11, *p* = 0.046). Men who had more concerns about using PrEP were less willing to use it (AOR = 0.40, 95% CI 0.30–0.53, *p*<0.001)
98	Intersecting barriers to PrEP awareness and uptake in black men who have sex with men in Atlanta, GA: a syndemic perspective	Matthew C. Sullivan et al., 2020	2017	USA	Cross‐sectional study	293 BMSM	Participants tended to be young (M = 31.1, SD = 9.6 SD) and identify as gay/homosexual or same gender loving (74.4%). Almost all participants (97.9%) were unmarried. Most (74.7%) reported some college education and the majority (50.9%) were not employed full time at the time of participation. Just under half (45.1%) of the sample reported an annual income below $20,000	260 (88.7%) indicated that they had heard of PrEP	77.5% (204/263) of those who had never used PrEP stated that they would use PrEP if given the option	BMSM who had been without healthcare coverage at any point during the past 2 years were less likely to have heard of PrEP (PR = 0.94, 95% CI 0.90, 0.98; *p* = 0.004), whereas BMSM who tested for HIV more frequently were significantly more likely to have heard of PrEP (PR = 1.04, 95% CI 1.01, 1.06; *p* <0.001). BMSM who identified as bisexual were less likely to have heard of PrEP than other BMSM (PR = 0.92, 95% CI 0.87, 0.98; *p* = 0.005), as were BMSM who were more “closeted” about their sexuality (X^2^ (2, 288) = 11.66, *p* = 0.003). BMSM who knew the HIV status of a greater proportion of their sexual partners were more likely to have heard of PrEP (PR = 1.07, 95% CI 1.01, 1.15; *p* = 0.016)
99	Willingness of community‐recruited men who have sex with men in Washington, DC to use long‐acting injectable HIV pre‐exposure prophylaxis	Matthew E. Levy et al., 2017	2014	Washington, DC, USA	Cross‐sectional study	314 MSM	Median 29.5 years; IQR 25–35; range 18–66. 50.0% (*n* = 157)were younger than 30 years old, 40.7% (*n* = 114) were non‐Hispanic black, 36.6% (*n* = 128) were non‐Hispanic white and 14% were Latino/Hispanic	Not reported	62.4% (*n* = 196/314)	In multivariable regression modelling, independent correlates of being very likely to use LAI PrEP included being <30 years old (aOR 1.64; 95% CI 1.00–2.68), having six or more (vs. one) sex partners in the last 12 months (aOR 2.60; 95% CI 1.22–5.53), having ever used oral PrEP (aOR 3.67; 95% CI 1.20–11.24) and being newly identified as HIV infected based on HIV testing for the study (aOR 4.83; 95% CI 1.03–22.67)
100	Preexposure antiretroviral prophylaxis attitudes in high‐risk Boston area men who report having sex with men: limited knowledge and experience but potential for increased utilization after education	Matthew J. Mimiaga et al., 2009	2007	Boston, USA	Cross‐sectional study	227 MSM	Age (mean = 40.8, SD = 9.1)	19% (*n* = 43)	74%	Less education, moderate income, no side effects from taking PrEP and not having to pay for PrEP
101	Acceptability of injectable and on‐demand pre‐exposure prophylaxis among an online sample of young men who have sex with men in California	Matthew R. Beymer et al., 2018	2015	California, America	Cross‐sectional study	265 MSM^41^	66.8% (*n* = 177) of the sample was 18–25 years of age and 76.6% (*n* = 203) identified as gay; 77.4% (*n* = 205) indicated they had sex with men only in the past 5 years; 44.2% (*n* = 117) was employed full time and 75.5% (*n* = 200) completed some college or more	Not reported	85.30%	Individuals who reported some college or more reported greater willingness to try injectable PrEP (adjusted odds ratio [aOR]: 2.92; 95% confidence interval [CI]: 1.32–6.46), on‐demand PrEP (aOR: 2.28; 95% CI: 1.06–4.90) or either method (aOR: 5.54; 95% CI: 1.78–17.22)
102	Perceived HIV risk, actual sexual HIV risk and willingness to take pre‐exposure prophylaxis among men who have sex with men in Toronto, Canada	Maya A. Kesler et al., 2016	2010–2012	Toronto, Canada	Cross‐sectional study	150 MSM	Median age was 44.5 years (IQR 37–50 years) and 82.88% (*n* = 121) of participants were white	Not reported	55% (67/122)	The remaining 45% of the HIV‐negative MSM were unwilling to take PrEP because they perceived their risk to be too low (64%), were concerned about the side effects (44%), did not like the burden of daily pill taking (16%) or had efficacy concerns (4%).
										Model 1: The odds of being willing to take PrEP were significantly higher for MSM with high actual sexual HIV risk (i.e. low condom use with regular HIV‐positive partners; component 2) compared to MSM with low actual HIV sexual risk (OR 27.11, 95% CI 1.33–554.43) after adjusting for the other actual HIV sexual risk component scores. Model 2: The odds of being willing to take PrEP were significantly higher for MSM who had high actual sexual HIV risk (low condom use with regular HIV‐positive partners; component 2) compared to MSM who had low actual sexual HIV risk (OR 29.85, 95% CI 1.39–640.53). Perceived HIV risk and actual general HIV risk were not significantly predictive of willingness to take PrEP. Model 3: When actual sexual HIV risk (PCA components) was removed from the model, the odds of being willing to take PrEP were significantly higher for MSM with high perceived HIV risk compared to MSM with low perceived HIV risk (OR 6.85, 95% CI 1.23–38.05). Again, actual general HIV risk was not significantly predictive of being willing to take PrEP (OR 1.87, 95% CI 0.54–6.54).
										
103	PrEP indicators, social capital and social group memberships among gay, bisexual and other men who have sex with men	Meagan Zarwell et al., 2019	2014	New Orleans, America	Cross‐sectional study	353 MSM	46% of the participants were black or other race (*n* = 162), 42% (*n* = 150) were under the age of 30 and 49% (*n* = 173) had completed college education	47% (*n* = 165)	62% (*n* = 219)	Black and other race men were less likely to be aware of PrEP than non‐Hispanic white men (*p* = 0.0016, χ^2^ = 9.93). Awareness of PrEP was also significantly related to age (*p*≤0.0001, χ^2^ = 37.83), recent STI testing (*p*≤0.0001, χ^2^ = 15.36), LGBTQ social group membership (*p*≤0.0001, χ^2^ = 35.04) and community group participation (*p*≤0.0001, χ^2^ = 19.41). Willingness to take PrEP was associated with age (*p* = 0.0036, χ^2^ = 11.30), recent STI testing (*p* = 0.0012, χ^2^ = 10.51) and social group membership (*p* = 0.0003, χ^2^ = 18.84)
104	Willingness to self‐pay for pre‐exposure prophylaxis in men who have sex with men: a national online survey in Taiwan	Nai‐Ying Ko et al., 2016	2014	Taiwan, China	Cross‐sectional study	1151 MSM^42^	The average age of the participants was 25.9 years (SD = 6.4, range, 18–53). 12.6% (*n* = 145) participants have university/postgraduate degree. 57.0% (*n* = 656) was employed	Not reported	56% (*n* = 645)	Multivariable analysis was conducted to identify factors correlated with willingness to self‐pay for PrEP in MSM and we found that willingness to self‐pay $340 (U.S.) for PrEP was significantly associated with the previous receipt of HIV nPEP (adjusted odds ratio [AOR], 3.02, 95% CI [1.49, 6.12], *p* = 0.002) and positive attitudes towards PrEP (AOR, 3.02, 95% CI [2.19, 4.17], *p* < 0.001)
105	Acceptability of an “on‐demand” pre‐exposure HIV prophylaxis trial among men who have sex with men living in France	Nicolas Lorente et al., 2011	2009	France	Cross‐sectional study	443 MSM^43^	Median age = 37 years (IQR = 30–44 years); 84% had completed secondary school and 46% had at least 5 years of tertiary education	Not reported	40% (*n* = 177)	Univariate analysis showed that men who were interested in participating in a PrEP trial, unlike their not‐interested counterparts, were significantly more likely to report both receptive anal intercourse with a casual partner (70% vs. 54%) and ICU during anal intercourse with casual partners (32% vs. 20%). Respondents interested in participating were also significantly more likely to report that they felt they could comply with the protocol's constraints, including the 2‐year follow‐up (80% vs. 38%), clinical visits every 3 months (89% vs. 63%) and possible adverse reactions to treatment (57% vs. 21%). They were significantly less stressed about not knowing whether they would receive a placebo or not (54% vs. 34%)
106	Transactional sex and preferences for pre‐exposure prophylaxis (PrEP) administration modalities among men who have sex with men (MSM)	Ofole Mgbako et al., 2018	2016	Paris, France	Cross‐sectional study	444 MSM^44^	The average age in the sample was 35.2 years (SD = 10.0 years); 79.3% of respondents (*n* = 352) were born in France; 84.7% (*n* = 376) identified as gay and 12.6% (*n* = 56) identified as bisexual; 13.5% (*n* = 60) reported having engaged in transactional sex	87.2% (*n* = 387)	45.10%	In the multivariate analysis, MSM who had engaged in transactional sex were more likely willing to use daily oral PrEP (aRR = 1.48; 95% CI = 1.11–1.98) or long‐acting injectable PrEP (aRR = 1.40; 95% CI = 1.09–1.81) than MSM who had not engaged in transactional sex
107	National trends in HIV pre‐exposure prophylaxis awareness, willingness and use among United States men who have sex with men recruited online, 2013 through 2017	Patrick S. Sullivan et al., 2020	2013–2017	USA	Cross‐sectional study	2013: 1907 MSM;^45^ 2014: 4082 MSM; 2015: 4513 MSM; 2016: 4267 MSM; 2017: 4475 MSM	2013: 411 (21.6%) age between 18 and 24 years, 2014: 658 (16.1%) age between 18 and 24 years, 2015: 1280 (28.4%) age between 18 and 24 years, 2016: 1172 (27.5%) age between 18 and 24 years, 2017: 1194 (26.7%) age between 18 and 24 years	2013: 47.4% (903/1907); 2014: 68.2% (2784/4082); 2015: 70.5% (3180/4513); 2016: 80.2% (3421/4267); 2017: 80.6% (3605/4475)	2013: 43.9% (822/1874); 2014: 55.4% (1520/2746); 2015: 56.8% (2367/4167); 2016: 59.0% (2180/3695); 2017: 59.5% (2132/3584)	Age and having health insurance
108	Community belonging and attitudes towards HIV pre‐exposure prophylaxis (PrEP) among transgender women	Paul A. D'Avanzo et al., 2021	2018	America	Cross‐sectional study	125 TGW	Mean age = 39 years (SD = 14.9)	87.00%	65% (81/125)	Few differences were observed between clusters on statements related to perceived benefits of PrEP. However, community unengaged members indicated less support for the statements that “PrEP would make me feel more in charge of my life,” that PrEP would only require taking one pill per day relative to the community active and community established clusters. Differences in perceived PrEP barriers were observed in comparisons of the community unengaged cluster with the other two. Those in the community unengaged cluster
										expressed higher levels of agreement with the statements that “PrEP is only for gay men” and that “using PrEP would make me feel less feminine” than both the community active and community established clusters, respectively. To a lesser extent, the community unengaged cluster also expressed greater agreement than the community active cluster with the statements “my doctor has never discussed PrEP with me so I must not need it” and “the treatments for HIV are so effective that I don't really need to take PrEP to be protected”.
109	Interest in HIV pre‐exposure prophylaxis in men who have sex with men in West Africa (CohMSM ANRS 12324 – Expertise France)	Pierre‐julien Coulaud et. 2018	2017	West Africa (Mali, Burkina Faso, Togo and Côte d'Ivoire)	Cross‐sectional study	564 MSM	Median age was 24 years (interquartile range, IQR = 21–27), 67% were single, 38% were students and 58% reported financial difficulties. Fifty‐four percent defined themselves as bisexual, while 52% declared they were sexually attracted to men only	15% (*n* = 84)	87% (488)	Interest in PrEP was associated with inconsistent condom use for anal sex (adjusted odds ratio [aOR]: 2.11; 95% confidence interval [CI] [1.21–3.67]), transactional sex (aOR: 2.02; 95% CI [1.11–3.71]), searching for male sexual partners on the internet in the previous month (aOR: 1.86; 95% CI [1.01–3.43]), having a high level of self‐esteem (aOR: 1.20; 95% CI [1.06–1.36]), having at least one STI at enrolment (aOR: 5.08; 95% CI [1.40–18.4]) and not being aware
										of PrEP (aOR: 2.03; 95% CI [1.04–3.96]). Participants having sex with HIV‐positive male partners on antiretroviral therapy (aOR: 0.28; 95%CI [0.11–0.74]), those being more sexually attracted to women than to men (aOR: 0.20; 95% CI [0.07–0.89]) and those reporting psychological and material support from close friends (aOR: 0.33; 95% CI [0.15–0.73]) were less interested in taking PrEP
110	Knowledge of and interest in using preexposure prophylaxis for HIV prevention among men who have sex with men in Thailand	R. Craig Sineath et al., 2013	2012	Thailand	Cross‐sectional study	404 MSM^46^	Mean age 25 (SD = 5.4)	7% (*n* = 28)	35% (*n* = 144)	The majority of respondents (65%; *n* = 191) indicated they would be willing to pay for PrEP
111	“That's kind of like the big struggle right now is can we get PrEP?”: facilitators and barriers to PrEP uptake among active duty gay and bisexual men	Raiza M. Beltran et al., 2021	2017–2018	America	Cross‐sectional study	93 gay and bisexual men (GBM)^47^	Median age = 29 years	Not reported	71% (67/93)	The only statistically significant demographic difference found between respondents who were and were not interested in PrEP was the respondents’ living arrangement (see Table 2)—interest in PrEP was reported by all GBM living on a military base, compared to approximately 2/3 of GBM living off‐base (*p* = 0.016)
112	Cost and anonymity as factors for the effective implementation of pre‐exposure prophylaxis: an observational study among gay, bisexual and other men who have sex with men in Singapore	Rayner Kay Jin Tan et al., 2018	2018	Singapore	Cross‐sectional study	1098 MSM^48^	6.6% (*n* = 73) of respondents identified as HIV positive, 74.8% (*n* = 821) as HIV negative and 18.6% (*n* = 204) reported being unaware of their HIV status. 16.6% (*n* = 182) of respondents reported that they had never disclosed their sexual orientation to any other person	81.20%	73.30%	Among those who would consider using PrEP (*n* = 486), 176 (36.2%) provided reasons as to why they would do so. Of these, 69.3% (*n* = 122) regarded PrEP as an effective means of preventing HIV acquisition, 12.5% (*n* = 22) regarded PrEP as a means of providing extra protection over current HIV prevention methods, 6.8% (*n* = 12) would use PrEP to engage in sex without condoms, 5.7% (*n* = 10) perceived themselves to be at high risk for HIV acquisition, 0.6% (*n* = 1) was in a serodiscordant relationship and 5.1% (*n* = 9) gave other reasons, such as the convenience of PrEP compared with condoms. Among those who would not consider using PrEP (*n* = 177), 129 (72.9%) provided reasons as to why they would not consider using PrEP. Of these, 28.7% (*n* = 37) perceived themselves to be at low risk of HIV acquisition, 17.8% (*n* = 23) cited barriers relating to cost and accessibility, 12.4% (*n* = 16) had doubts about the efficacy of PrEP, 12.4% (*n* = 16) had concerns over how PrEP would lead to risk compensation in the community,
										11.6% (n = 15) had concerns over the side effects of PrEP, 5.4% (n = 7) did not have enough information on PrEP, 5.4% (n = 7) regarded PrEP as inconvenient and 6.2% (n = 8) gave other reasons, such as preferring sex with condoms
113	Evaluation of a pre‐exposure prophylaxis programme for men who have sex with men and transgender women in Thailand learning through the HIV prevention cascade lens	Reshmie A. Ramautarsing et al., 2020	2019	Thailand	Cross‐sectional study	3863 MSM 528 TG	MSM: Median age = 26 years (IQR 22, 32); TGW: Median age = 25 years (IQR 21, 30)	Not reported	MSM: 1856/3863 (48.0%); TGW: 232/528 (43.9%)	Among 2007 MSM not accepting PrEP, 938 (46.7%) perceived no or low risk, 385 (19.2%) did not want to take pills, 147 (7.3%) wanted to start at a later visit, 142 (7.1%) felt condom use was enough to prevent HIV, 102 (5.1%) could not come back for follow‐up visit, 55 (2.7%) were not interested and 53 (2.6%) were afraid of side effects. Among 296 TGW not accepting PrEP, 124 (41.9%) perceived no or low risk, 68 (23.0%) did not want to take pills, 15 (5.1%) wanted to start at a later visit, 24 (8.1%) felt condom use was enough for HIV prevention, 17 (5.7%) could not come back for follow‐up visit, nine (3.0%) were afraid of side effects and two (0.7%) were not interested
114	Knowledge and use of HIV pre‐exposure prophylaxis among men who have sex with men in Berlin – a multicentre, cross‐sectional survey	Ricardo Niklas Werner et al., 2018	2017–2018	Berlin, Germany	Cross‐sectional study	470 MSM^49^	The mean age of the participants was 37.4 years (SD: 11.9; range: 18–79 years) and 94.0% indicated that they lived in Berlin. Around two‐thirds (65.3%) of the participants had a university degree	90% (*n* = 423)	42.40%	“Higher risk (CAI)”
115	Acceptability of condoms, circumcision and PrEP among young black men who have sex with men: a descriptive study based on effectiveness and cost	Richard A. Crosby et al., 2014	2013	Mississippi, Louisiana, Alabama and Georgia, America	Cross‐sectional study	95 YBMSM^50^	Mean age = 26.8 years (SD = 5.66 years), age range = 18–39 years	Not reported	70.5% (*n* = 67)	Willingness to accept PrEP decreased with a lower level of effectiveness: 75% effectiveness (43%) and 50% effectiveness (21%). Cost had an influence on men's willingness to accept PrEP: 19% were willing to accept the medication with a personal cost of $100
116	PrEP use awareness and interest cascade among MSM and transgender women living in Bali, Indonesia	Rissa Cempaka et al., 2020	2017–2018	Indonesia	Cross‐sectional study	220 MSM	Median age = 28 (IQR = 24–32), under one‐third (30.0%) was university educated	16.4% (36/220)	74.5% (*n* = 164/220)	Minimal risk for HIV infection, unwanted side effects and condoms used
117	HIV testing, knowledge and willingness to use PrEP among partnered men who have sex with men in South Africa and Namibia	Rob Stephenson et al., 2021	November 2016–March 2017	South Africa and Namibia	Cross‐sectional study	254 MSM^51^	Participants ranged in age from 18 to 55 years, with 40.9% aged 18–24 years and 11.4% aged over 35 years. Participants predominantly identified as black African (55.9%) and gay (70.1%). Approximately one‐quarter had education at university/college level or higher (27.6%) and 52.4% reported current fulltime employment	63.6% (*n* = 161) of participants reporting having heard of PrEP	Men reported low levels of willingness to use pre‐exposure prophylaxis (PrEP) (16%, *n* = 41)	Few factors were significantly associated with knowledge of PrEP. Men in South Africa (aOR 1.88, 95% CI 1.12, 2.87) and those with higher levels of education (aOR 1.89, 95% CI 1.17, 3.12) were more likely to have heard of PrEP. Relative to men aged 18–24, men of all other ages ( 25–34 years: aOR 1.54, 95% CI 1.10, 1.65; 35+ years: aOR 2.23, 95% CI 1.45, 3.64) were significantly more likely to report being willing to use PrEP in their current relationship. Again, men in South Africa (aOR 3.32 95% CI 1.85, 4.65) and men with higher levels of education (aOR 2.12, 95% CI 1.50, 3.48) were more likely to report being willing to use PrEP
118	Dyadic influences on pre‐exposure prophylaxis (PrEP) use and attitudes among male couples	Rob Stephenson et al., 2021	October 2017–January 2018	USA	Cross‐sectional study	764 MSM^52^	The sample was largely non‐Hispanic white (74.0%) and between the ages of 25 and 34 (59.9%). The majority of individuals were college graduates (35.9%) or had graduate degrees (36.9%) and employed fulltime (81.4%). The sample was predominantly gay identifying (92.5%)	Not reported	42.3% (236/556) were willing to use PrEP in future	African American men reported an increased likelihood of taking PrEP in the future (beta 0.4448, *p*‐value 0.014) than non‐Hispanic white men. Men who reported substance use in the previous 3 months reported a lower likelihood of using PrEP in the future (beta− 0.463, *p*‐value 0.016).Participants and their partners who reported an increased risk of HIV acquisition also reported an increased likelihood of future PrEP use (men beta 0.144, *p*‐value 0.000: partner beta 0.219, *p*‐value 0.034). Men who reported a higher perceived prevalence of HIV among GBMSM nationally reported higher likelihood of future use (beta 0.296, *p*‐value 0.023). Men who reported having more CAS with casual partners were more likely to use PrEP in the future (beta 0.638, *p*‐value 0.004). Men who reported being more comfortable talking to their partners about PrEP were more likely to take PrEP in the future (beta 0.205, *p*‐value 0.007)
119	“How I wish this thing was initiated 100 years ago!” Willingness to take daily oral pre‐exposure prophylaxis among men who have sex with men in Kenya	Robinson Njoroge Karuga et al., 2016	2013	Kisumu and Nairobi, Kenya	Cross‐sectional study	80 MSM	68.8% reported being HIV negative (*n* = 55). The analyses in the rest of this paper will focus on the HIV‐negative MSM. The participants’ median age was 24.9 years (IQR = 5 years), with the majority (89%) being in the age group 18–29 years	Not reported	83%	Sexual orientation
120	Perceptions of and intentions to adopt HIV pre‐exposure prophylaxis among black men who have sex with men in Los Angeles	Ronald A. Brooks et al., 2015	2012–2013	Los Angeles, USA	Cross‐sectional study	224 BMSM^53^	Participants ranged in ages from 18 to 65 years (M = 33.5, SD = 11.8) and were equally divided between younger (18–29 years) and older (30+ years) participants. The overwhelming majority (96%) of men identified as gay or bisexual. Participants were primarily lower socioeconomic status (SES), with 67% having very low incomes, 51% not working and 46% with only a high school education or less	33.0% (*n* = 74)	59.8% (*n* = 134)	Participants agreeing with the statements: “I would be very uncomfortable taking HIV medicine when I don't have HIV” (AOR = 0.39, 95% CI = 0.16–0.91) and “Not knowing if there are long‐term side effects of taking a daily HIV medicine makes me very uncomfortable” (AOR: 0.36, 95% CI = 0.14–0.88) were less likely to indicate a high‐PrEP adoption intention compared with participants who disagreed with these statements. In contrast, positive views were independent predictors of a high‐PrEP adoption intention. Participants agreeing with the statements: “I would be one of the first people to use PrEP if it were available” (AOR = 4.13, 95% CI = 1.74–9.81) and “Taking a daily HIV medicine would be a good way to protect myself from getting HIV”
										(AOR = 2.26, 95% CI = 1.6–3.17) were more likely to indicate a high PrEP adoption intention compared with participants who disagreed with these statements. Age was the only demographic predictor of future PrEP use. Younger participants (18–29) were two times more likely than older participants (30+) to indicate a high intention to adopt PrEP (AOR = 2.29, 95% CI = 1.06–4.93)
121	Predictors of awareness, accessibility and acceptability of pre‐exposure prophylaxis (PrEP) among English‐ and Spanish‐speaking Latino men who have sex with men in Los Angeles, California	Ronald A. Brooks et al., 2019	2015–2017	Los Angeles and California, USA	Cross‐sectional study	260 MSM^54^	More than two‐thirds were 18–35 years old	85%	71% (among non‐PrEP users)	Age, education and risk behaviour
122	Awareness, willingness, and use of pre‐exposure prophylaxis among men who have sex with men in Washington, DC and Miami‐Dade County, FL: National HIV Behavioral Surveillance, 2011 and 2014	Rudy Patrick et al., 2017	2016	Washington, DC and Miami, America	Cross‐sectional study	Washington, DC: 323 MSM;313 MSM (2011;2014) Miami: 279 MSM;431 MSM (2011;2014)	Washington, DC: MSM in 2011 versus 2014 reported at least some college education (83.9% vs. 91.4%), having received an HIV test in the last 12 months (69.7% vs. 77.3%). Miami: MSM in 2011 versus 2014 reported at least some college education (69.2% vs. 62.4%), having received an HIV test in the last 12 months (58.8% vs. 71.2%)	Between 2011 and 2014: DC (39.1% [*n* = 126]–73.8% [*n* = 231]) and Miami (19.4% [*n* = 54]–41.2% [*n* = 261])	(61.0% [*n* = 197]; 48.2% [*n* = 151], respectively, in 2011 and 2014) (48.0% [*n* = 134]; 60.6% [*n* = 261], respectively, in 2011 and 2014)	Washington, DC 2011: younger MSM (18–24 years old) were more likely (OR = 2.28; 9% CI: 1.08–4.84) to report being very likely to use PrEP compared with MSM 35 years or older. Hispanic MSM also had higher odds of being very likely to use PrEP compared with white MSM (OR = 3.85; 95% CI: 1.56–9.51). MSM reporting 2–5 male sexual partners in the last 12 months had reduced odds of being very likely to use PrEP compared with those with six or more partners (OR = 0.55; 95% CI: 0.31–0.95). Washington, DC 2014: the independent correlates of being very likely to use PrEP included being
										black (OR = 1.80; 95% CI: 1.04–3.13) and having 1 (OR = 0.38; 95% CI: 0.18–0.80) or 2–5 (OR = 0.45; 95% CI: 0.27–0.75) compared with 6 or more male sexual partners in the last 12 months. Miami 2011: independent correlates in 2011 included being Hispanic (OR = 0.39; 95% CI: 0.16–0.93), reporting non‐injection drug use in the last 12 months (OR = 0.54; 95% CI: 0.32–0.92) and an annual income $20,000–$39,999 versus $$40,000 (OR = 1.86; 95% CI: 1.01–3.41). Miami 2014: being Hispanic (OR = 2.19; 95% CI: 1.09–4.41) and newly identified as HIV positive (OR = 2.87; 95% CI: 1.27–6.50) were associated with increased odds of being very likely to use PrEP among MSM
123	Links between sexual orientation and disclosure among black MSM: sexual orientation and disclosure matter for PrEP awareness	Ryan J. Watson et al., 2019	2017–2019	Atlanta, USA	Cross‐sectional study	345 MSM	Average age was 31.3 years old	90.7% (*n* = 313)	Not reported	Sexual orientation
124	Young transgender women's attitudes toward HIV pre‐exposure prophylaxis	Sarah M. Wood et al., 2016	2015	Philadelphia, USA	Cross‐sectional study	25 MSM	Mean age was 21.2 years	64%	28%	Cost and economic conditions, worry about the adverse reaction of the drug and shame
125	Mental health, social influences, and HIV pre‐exposure prophylaxis (PrEP) utilization among men and transgender individuals screening for HIV prevention trials	Sarah M. Wood et al., 2020	July 2016–May 2018	Philadelphia, USA	Cross‐sectional study	247 MSM	83 (34%) participants aged 18–24 years; 115 (47%) aged 25–34 years; 49 (20%) aged 35+ years	93% (*n* = 229) were aware of PrEP	92% (*n* = 225) were willing to take PrEP	For those unwilling to take PrEP (*n* = 19), the most common reasons were not wanting to take daily medication (6%), concern for side effects (2%) and concern about the expense of PrEP (2%)
126	The pre‐exposure prophylaxis cascade in at‐risk transgender men who have sex with men in the United States	Sari L. Reisner et al., 2021	2017	USA	Cross‐sectional study	843 TG	The mean age was 28.1 years (standard deviation = 7.1 years) and 4.8% were black, 21.7% Latinx and 25.6% another race/ethnicity	PrEP awareness was high, with 84.1% (709/843) having heard of PrEP	67.3% (567/843) reported interest in PrEP as a daily oral pill	The most common reasons why trans MSM were not interested in PrEP were feeling they are not at risk (68.5%), being concerned about cost (24.2%), concerned about side effects (20.1%) and concerned about interference with hormones (14.6%)
127	PrEP willingness and adherence self‐efficacy among men who have sex with men with recent condomless anal sex in urban China	Shufang Sun et al., 2021	2018	China	Cross‐sectional study	622 MSM	Participants’ age ranged from 18 to 62 (mean = 29.75, median = 28, standard deviation [SD] = 8.32). Participants’ education level varied: 53.54% (*n* = 333) had college degree or higher, 25.24% had associate degree (*n* = 157), 16.7% completed high school (*n* = 104) and 4.50% had junior high school or below (*n* = 28)	A total of 56.4% (*n* = 351) of participants were aware of PrEP	A total of 64.6% (*n* = 402) indicated a willingness to take oral PrEP if provided for free	In multivariate analysis, willingness to take oral PrEP was positively associated with being a migrant, aOR (adjusted odds ratio) = 2.01, 95% CI (confidence interval) = (1.38, 2.92), prior PrEP use (aOR = 6.17 [1.98, 27.40]), sex under the influence of substance in the past 6 months, (aOR = 2.57 [1.67, 4.03]) and having an HIV + partner in the past 6 months (aOR = 4.19 [1.82, 11.43]). Willingness to take oral PrEP was negatively associated with sexual orientation concealment (aOR = 0.83 [0.70, 0.96]), having tested for HIV in the past 6 months (aOR = 0.50 [0.34, 0.74]) and WeChat use for HIV prevention (aOR = 0.84 [0.72, 0.98])
128	The intention to use HIV‐pre‐exposure prophylaxis (PrEP) among men who have sex with men in Switzerland: testing an extended explanatory model drawing on the unified theory of acceptance and use of technology (UTAUT)	Sibylle Nideröst et al., 2018	2015	Switzerland	Cross‐sectional study	556 MSM^55^	Mean age = 40.5 years (SD = 11.9 years), age range = 15–81 years	Not reported	26.4% (*n* = 147)	The analysis revealed that participants’ intention to use PrEP was predicted by the four PrEP‐related aspects: performance expectancy (β = 0.25), effort expectancy (β = −0.19), social influence (β = 0.31) and concerns (β = −0.15)
129	Willingness to use pre‐exposure prophylaxis for HIV prevention among men who have sex with men in Malaysia: findings from an online survey	Sin How Lim et al., 2017	2016	Kuala Lumpur, Malaysia	Cross‐sectional study	990 MSM^56^	The mean age was 30.60 (range 18–68) years. 60.1% (*n* = 595) participants were single, 80.4 (*n* = 796) identified themselves as gay, 87.2% (*n* = 863) highly educated and 67.8 (*n* = 671) working full time	43.6% (*n* = 432)	39% (*n* = 387)	In the multiple logistic regression model, Malay ethnicity (AOR: 1.73, 95%CI: 1.12–2.70), gay sexual identity, having two or more male sex partners in the past 6 months (AOR: 1.98, 95% CI: 1.29–3.05), having heard of PrEP (AOR: 1.40, 95% CI: 1.06–1.86), having a lack of confidence in practising safer sex (AOR: 1.36, 95%CI: 1.02–1.81) and having ever paid for sex with a male partner were independently (AOR: 1.39, 95% CI: 1.01–1.91) associated with willingness to use PrEP. Men who are identified as heterosexual are less willing to use PrEP (AOR: 0.36, 95% CI: 0.13–0.97)
130	Seasons of risk: anticipated behavior on vacation and interest in episodic antiretroviral pre‐exposure prophylaxis (PrEP) among a large national sample of U.S. men who have sex with men (MSM)	Steven A. Elsesser et al., 2015	2013	America	Cross‐sectional study	7305 MSM^57^	Mean age = 43.2 years (SD = 12.7 years); 82% identified as gay, 85.7% were white, 68.4% of the sample were college graduates and 86.3% had health insurance	Not reported	74.3%	MSM who reported increased CAS while on vacation in the past year were more likely to indicate that they would take PrEP if it were helpful when used for short periods than respondents who did not (aOR = 2.02, 95% CI 1.59–2.56, *p*<0.001)
131	Awareness and acceptability of pre‐exposure prophylaxis among men who have sex with men in Baltimore	Susan Fallon 2015	2014	Baltimore, America	Cross‐sectional study	399 MSM^58^	Median age = 30 years (age range = 18‐71 years); 84% were of a minority race and 44% reported more postsecondary education; 53% reported full or part time employment, 29% were unemployed and 57% reported an annual household income of less than $20,000; 60% identified as being homosexual/ gay	11% (*n* = 44)	48% (*n* = 191)	In bivariate analyses, lower educational attainment (OR 0.63 CI 0.42–0.94) was associated with decreased PrEP acceptability and perceiving discrimination against people with HIV (OR 1.62, CI 1.08–2.43) and having a positive HIV test result (OR 1.59, CI 1.01–2.59) were significantly associated with an increased willingness to use PrEP. In multivariable analysis, after adjusting for other covariates, perceiving discrimination was also significantly associated with increased willingness to take PrEP every day to prevent from getting HIV (AOR 1.5 CI 1.01–2.51)
132	Changes in HIV preexposure prophylaxis awareness and use among men who have sex with men — 20 urban areas, 2014 and 2017	Teresa Finlayson et al., 2019	2014–2017	America	Cross‐sectional study	2014: 3821 MSM 2017: 4052 MSM^59^	In 2014, 50.7% (*n* = 1939) of the participants were 18–29 years and 49.3% (*n* = 1882) were ≥30 years; in 2017, 46.4% (*n* = 1882) of the participants were 18–29 years and 53.6% (*n* = 2170) were ≥30 years	2014: 59.8% (*n* = 2286) 2017: 90.4% (*n* = 3664)	2014: 5.7% (*n* = 216) 2017: 35.1% (*n* = 1425)	The difference in reported PrEP use between black (26%) and white (42%) MSM remained significant after controlling for income, health insurance and region (aPR = 0.78; 95% CI = 0.66–0.92). During 2017, PrEP use increased with education and income, and 39% of the MSM who saw a healthcare provider in the past 12 months reported PrEP use
133	Awareness of prevention strategies and willingness to use preexposure prophylaxis in Brazilian men who have sex with men using apps for sexual encounters: online cross‐sectional study	Thiago Silva Torres et al., 2018	2016	Brazil	Cross‐sectional study	5065 MSM	Median age was 30 years	57.89%	52.38%	High number of male sexual partners, condomless receptive anal intercourse, sex with HIV‐positive partner, high and unknown perceived likelihood of getting HIV in the next year, STI diagnosis and PrEP awareness
134	Interest in taking HIV pre‐exposure prophylaxis is associated with behavioral risk indicators and self‐perceived HIV risk among men who have sex with men attending HIV testing venues in Sweden	Tobias Herder et al., 2020	2018	Sweden	Cross‐sectional study	658 MSM	Median age = 32 years (IQR 27–41)	Not reported	68.8% (453/658)	The number of male sexual partners was higher among those interested in PrEP as compared to those not interested. The descriptive analysis showed (χ2 *p* < 0.05) that among the respondents who were interested in PrEP, there was a higher proportion of respondents with higher knowledge about PrEP, higher self‐perceived HIV risk, higher number of male sexual partners and receptive condomless anal intercourse (rCLAI) partners and more often reported drug use during sex, hard drug use, poppers use and sex abroad in the last 12 months
135	PrEP awareness and engagement among transgender women in South Africa: a cross‐sectional, mixed methods study	Tonia Poteat et al., 2020	2018	South Africa	Mixed method study	213 TG	The median age was 26 years (range 18–59)	57 (45%) of 127 HIV‐negative participants were PrEP‐aware	Only 56 of 102 (55%) HIV‐negative survey participants who were not currently taking PrEP reported willingness to take it	On bivariate analysis of survey data, lifetime sexual violence (OR: 1.92 95% CI 1.10–3.35) was associated with higher likelihood of PrEP willingness. Current employment (OR: 0.52 95% CI 0.28–0.97) and greater community‐connectedness (OR: 0.90 95% CI 0.81–1.00) were associated with a lower likelihood of PrEP willingness on bivariate analyses. However, only community‐connectedness (aOR: 0.87 95% CI 0.77–0.99) remained significantly (and negatively) associated with PrEP willingness in multivariable models.
136	Bridging awareness and acceptance of pre‐exposure prophylaxis among men who have sex with men and the need for targeting chemsex and HIV testing: cross‐sectional survey	Tsz Ho Kwan et al., 2019	2014	Hong Kong, China	Cross‐sectional study	453 MSM^60^	249 (55.0%) participants were aged 25 years or younger; 270 (59.6%) had a monthly income of less than HK $15,000; 249 (55.0%) and 131 (28.9%) were full‐time workers and students, respectively; 79 (17.4%) attained secondary‐level education or below; and 359 (79.2%) were gay	49.7% (*n* = 225)	78.40%	In the univariate analysis of the factors associated with the acceptance of PrEP, acceptance was associated with working or studying fulltime (OR 2.19, 95% CI 1.27–3.78; *p* = 0.004) but not with other demographic variables. In the multivariate regression model, working or studying fulltime (aOR 2.36, 95% CI 1.33–4.22; *p* = 0.004), having been tested for HIV (aOR 1.58, 95% CI 0.98–2.56; *p* = 0.06), having an emotionally attached partner as the only sex partner in the previous year (aOR 0.27, 95% CI 0.13–0.56; *p*<0.001) and considering partner's condom use habit important (aOR 4.08, 95% CI 2.15–7.75; *p*<0.001) were predictors of PrEP acceptance
137	Using PrEP to #STOPHIVATL: findings from a cross‐sectional survey among gay men and transgender women participating in Gay Pride Events in Atlanta, Georgia, 2018	Udodirim Onwubiko et al., 2020	2018	Georgia, USA	Cross‐sectional study	277 MSM	The median age was 31 years and 54% were black/African American. The majority reported having college or advanced education (77%), being employed (90%), possessing health insurance (81%)	87% (*n* = 240) reported being aware of PrEP	Among respondents who reported never using PrEP (*N* = 196), 69 (37%) were interested in taking PrEP	In unadjusted analysis, being aware of PrEP was independently associated with higher level of education (OR: 0.24 [95% CI: 0.11, 0.53]), being employed (OR: 0.35 [95% CI: 0.13, 0.89]), having an income over $60,000 (OR: 0.07 [95% CI: 0.01, 0.55]), possession of health insurance (OR: 0.22 [95% CI: 0.10, 0.50]), having a history of stable housing (OR: 5.24 [95%CI: 2.17, 12.65]), reporting an encounter with a clinician in the previous year (OR: 0.28 [95% CI: 0.12, 0.65]) and recent use of illicit drugs (OR: 0.25 [95% CI: 0.10, 0.63]). Only encounter with a clinician within the past year remained significantly associated with PrEP awareness in the multivariate model, with MSM/TWSM who reported having no encounter with a clinician in the preceding 12 months showing significantly lower odds (74% lower) of being aware of PrEP compared to those who reported having a clinician encounter in the past year (aOR: 0.26 [95% CI: 0.08, 0.78]). The top five reasons for never using PrEP were low perception of personal risk (37%), poor knowledge of PrEP (37%), concern about potential side effects (15%), financial costs of PrEP (13%) and no knowledge of where to get PrEP (7%)
138	Pre‐exposure prophylaxis awareness and use among cisgender men who have sex with men and use methamphetamine in 3 western US cities	Vanessa M. McMahan et al., 2020	2017	Seattle, Portland and Denver, USA	Cross‐sectional study	881 MSM^61^	Of the total participants, 88 reported methamphetamine use and 793 did not report methamphetamine use in the past year	95% (833/881) participants had heard of PrEP (833/881)	Not reported	Pre‐exposure prophylaxis awareness was lower among MSM who used methamphetamine (*p* = 0.01)
139	Acceptability of HIV pre‐exposure prophylaxis (PrEP) and implementation challenges among men who have sex with men in India: a qualitative investigation	Venkatesan Chakrapani et al., 2015	2014	Chennai and Mumbai, India	Qualitative study	61 MSM	Mean age = 26 years (SD = 4.8 years); 54.1% (*n* = 33) completed high school, 32.8% (*n* = 20) completed college education; 77.0% (*n* = 47) were employed	None had heard of PrEP	55.7% (*n* = 34)	Facilitators to PrEP acceptability among MSM: potential for covert use, sex without condoms and anxiety‐less sex. Barriers to PrEP acceptability among MSM: stigma associated with PrEP use, fear of disclosures to one's family, wife or male steady partner and being labelled as HIV positive or promiscuous by peers
140	Acceptability of HIV pre‐exposure prophylaxis among transgender women in India: a qualitative investigation	Venkatesan Chakrapani, 2020	2017	India	Qualitative study	36 TGW	Mean age = 26.1 years (SD = 4.8)	2.8% (*n* = 1)	Not reported	Affective attitudes: high efficacy and the ability to use it covertly Burden: cost, adherence to a daily regimen and hiding PrEP from family members
141	Factors influencing willingness to use human immunodeficiency virus preexposure prophylaxis among transgender women in India	Venkatesan Chakrapani, 2021	2017	India	Cross‐sectional study	355 TGW	Median age = 26 years; 50.7% had not completed high school	17.1% (61/355)	94.6% (*n* = 336)	In sex work, study in high school or above, discrimination, healthcare and anal sex
142	Acceptability and influencing factors of pre exposure prophylaxis among men who have sex with men in Guangxi	Wei et al., 2011	2011	Guangxi, China	Cross‐sectional study	650 MSM	Median age = 27 years; 62.2% were Han nationality and 33.1% were Zhuang nationality; 61.3% had a high school degree or below, 21.7% had a college degree and 17.0% had a bachelor degree or above; 48.6% reported a monthly income of less than 1000 yuan, 42.6% reported a monthly income of 1000–3000 yuan, 8.8% reported a monthly income of more than 3000 yuan	19.7% had heard of PrEP	91.90%	Data from logistic regression analysis showed that those who had found partners through friends (OR=6.21, *p*=0.020) and those who would advise his friend to use PrEP (OR=39.32, 95 CI%:17.77–86.97, *p*=0.000) were more likely to accept PrEP. Those who thought they could protect themselves from HIV infection (OR=0.32, *p*=0.010) or not having sex with the ones who refused to use a condom (OR=0.34, *p*=0.010) were less likely to accept PrEP
143	Awareness of and preferences for preexposure prophylaxis (PrEP) among MSM at high risk of HIV infection in Southern China: findings from the T2T study	Weiying Chen et al., 2021	2017–2018	China	Cross‐sectional study	550 HIV‐negative MSM were enrolled in the study	Median age = 26 (IQR 23–31) years. The majority of participants had a university degree or above (67.3%, 370/550)	43.1% (237/550)	The overall willingness to use PrEP was 65.8% (362/550)	Alcohol consumption, using gay dating apps in the past 6 months, ever participated in HIV or STD‐related studies and had heard of PrEP
144	Prepared for PrEP: preferences for HIV pre‐exposure prophylaxis among Chinese men who have sex with men in an online national survey	Wenting Huang et al., 2019	2017	China	Cross‐sectional study	979 MSM	The median age was 26	18.1% (*n* = 177)	90% (*n* = 882)	Ever heard about PrEP, multiple male sexual partners were more likely to be interested in oral PrEP
145	Correlates of awareness of and willingness to use pre‐exposure prophylaxis (PrEP) in gay, bisexual, and other men who have sex with men who use geosocial‐networking smartphone applications in New York City	William C. Goedel et al., 2016	2015	New York, America	Cross‐sectional study	152 MSM	Mean age = 29.59 years (SD = 8.99 years); 83.6% identified as gay; 56.6% were non‐white, 11.2% identified as black or African American and 26.3% identified as Hispanic or Latino; 98.6% completed at least high school or some equivalent, 56.4% completed a bachelor's degree or higher. 69.1% reported earning less than $50,000 per year	85.50%	57.60%	In the multivariable models, among those not currently taking the medication, being unwilling to take PrEP in the future was associated with being 26–30 years old (3.472; 95% CI 1.130, 10.638; *p* = 0.030) and reporting current non‐use due to concerns about side effects (3.300; 95% CI 1.412; 7.692; *p* = 0.006)
146	Understanding willingness to use oral pre‐exposure prophylaxis for HIV prevention among men who have sex with men in China	Xia Wang et al., 2018	2015	Wuhan and Shanghai, China	Cross‐sectional study	487 MSM	Age range = 18–61 years, mean age = 27.68 years (±7.15 years); 22.4% (*n* = 109) were married to a woman and 73.1% (*n* = 356) had a college or higher level of education	19.1% (*n* = 93)	71.3% (*n* = 347)	The main reasons for being unwilling to use PrEP were being worried about side effects (72.9%), the necessity of taking PrEP for long periods of time (54.3%) and cost (40.4%). Compared with respondents whose marital status was single, divorced or widowed, men who were married to a woman were more likely to explain their willingness to use PrEP (88.0% vs. 77.6%; χ^2^ = 4.638; *p*<0.05). Men who reported never using condoms with their regular sexual partners were more likely to explain their willingness to use PrEP compared to men who said they always used condoms (39.4% vs. 6.3%; χ^2^ = 22.93; *p*<0.01)
147	Willingness and influencing factors of using pre‐exposure prophylaxis among 301 men have sex with men in Wuhan city, 2015	Xie et al., 2017	2015	Wuhan, China	Cross‐sectional study	301 MSM	Mean age = 27.51±8.31 years; age range = 18–61 years; 42.52% of the respondents reported a monthly income of 3001–5000 yuan and 20.93% reported a monthly income of more than 5000 yuan; over 50% of the respondents did not had a regular sexual partner	17.28% (*n* = 52)	74.42% (224/301)	Among those who had regular homosexual partners, results suggested that those who were married/cohabiting were more likely to report a willingness to use PrEP compared to unmarried/divorced or widowed (OR = 5.60, 95%CI: 1.81–17.29), compared with homosexual, heterosexuality was associated with decreased odds of willingness to use PrEP (OR = 0.22, 95% CI: 0.06–0.82), compared with HIV status of sexual partner was negative or uncertain, positive infection status was associated with increased odds of willingness to use (OR = 7.52). Compared with MSM who have not regular homosexual partners, those who were married/cohabiting were more likely to report a willingness to use PrEP compared to unmarried/divorced or widowed (OR = 9.09, 95% CI: 1.04–79.65), compared with those who think they have risk of infection, those who do not think they have risk of infection was associated with decreased odds of willingness to use PrEP (OR = 0.30), compared with those with a high frequency to seek sexual partners, those not often to seek was associated with decreased odds of willingness to use PrEP (OR = 0.27, 95% CI: 0.11–0.67)
148	Analysis of willingness and influencing factors for usage of pre‐exposure prophylaxis among men who have sex with men	Xue et al., 2015	2013	China	Cross‐sectional study	760 MSM^62^	The ages of surveyed MSM were between 18 and 60, 58.2% (442) were single, 78.9% (600) of them have received college education, 60.4% (459) received annual income less than 60,000 yuan, 73.3% (557) only had sex with men and 77.2% (587) self‐reported being gay	72.8% indicated that they “completely understand” and “basically understand” the content of pre‐exposure prophylaxis medication	32.10%	In multivariate logistic regression analysis, it suggested that annual income under 60,000 yuan (OR = 0.64, 95% CI: 0.45–0.92) and understanding PrEP (OR = 1.98, 95% CI: 1.36–2.88) were influencing factors for usage of PrEP among MSM
149	Attitudes toward HIV pre‐exposure prophylaxis among men who have sex with men in Western China	Yan Zhang et al., 2013	2009–2010	Western China	Cross‐sectional study	1402 MSM	The median age was 26 years	22% (*n* = 310)	77%	Lower education, income, STI history, having previously heard of PrEP and believing PrEP was effective
150	Low willingness and actual uptake of pre‐exposure prophylaxis for HIV‐1 prevention among men who have sex with men in Shanghai, China	Yingying Ding et al., 2016	2012–2013	Shanghai, China	Cross‐sectional study	1033 MSM	The majority were younger than 35 years (76.7%), non‐local residents (59.3%), with at least college education (62.5%), never married (74.2%) and self‐identified as a gay (76.0%). Overa half of the participants had HIV/AIDS knowledge score of 5–6, only a few participants (5%) had ever used drugs	Not reported	197 (19.1%)	Univariate analysis indicated that significant variables associated with willingness to use PrEP included age, permanent legal residency, occupation, education, number of male sex partners in the past 6 months and condom use at last anal sex. In multivariate analysis, those who were aged ≥ 45 years (OR = 2.18; 95% CI: 1.13–4.23), non‐local residents (OR = 1.69; 95% CI: 1.16–2.45), had two or more male sex partners in the past 6 months (OR = 1.53; 95% CI: 1.07–2.17 for 2–5 and OR = 1.82; 95% CI: 1.05–3.17 for ≥ 6, respectively) were significantly more willing to use PrEP, whereas those reporting condom use at last anal sex with man were significantly less willing to use PrEP (OR = 0.68; 95% CI: 0.47–0.97)
151	Willingness to use HIV pre‐exposure prophylaxis and associated factors among men who have sex with men in Liuzhou, China	Yuansheng Fu et al., 2021	2017–2019	Liuzhou, China	Cross‐sectional study	829 MSM	15–24 years: 41.3% (*n* = 342), 25–34 years: 39.0% (*n* = 323), over 35: 19.8% (*n* = 164)	Not reported	30.3% (251/829)	In the univariate analysis, ethnicity, education level, monthly income, sexual orientation, history of STDs, recruitment source, ever had oral sex with a man, had casual sex other than commercial sex in the past 6 months and history of HIV test were significantly associated with willingness to use PrEP
152	Preexposure prophylaxis comprehension and the certainty of willingness to use preexposure prophylaxis among men who have sex with men in China	Zhi‐Wei Zheng et al., 2018	Not reported	China	Cross‐sectional study	541 MSM^63^	50.8% were younger than 30 years of age	34.8% (188/541)	36.2% (*n* = 196)	Concerns about side effects, concerns about effectiveness, low perception of personal risk, low perception of partner risk and poor medication adherence
153	Acceptability and influencing factors of pre‐exposure prophylaxis among men who have sex with men in Chongqing	Zhong et al., 2013	July 2009–March 2010	Chongqing, China	Cross‐sectional study	448 MSM	Median age was 25 years; 96.9% were Han nationality; 45.1% had a bachelor degree or above, 26.2% had a college degree, 24.2% had a high school degree, 4.5% had a junior high school degree or below; 84.1% were from city, 15.9% were from country; 45.9% had a monthly income of 1000–3000 yuan, 19.5% had a monthly income of less than 1000 yuan, 19.2% had a monthly income of more than 3000 yuan and 15.4% did not have an income	50.5% had heard of PrEP	76.20%	Multivariate logistic regression analysis indicated that the factors significantly associated with acceptability of PrEP among MSM were: monthly income (≤1000 rmb OR: 4.775, 95% CI 1.274–17.895, *p* = 0.0204; 1001–3000 rmb OR: 1.945, 95% CI 0.594–6.376, *p* = 0.2718; 3001–5000 rmb OR: 1.427, 95% CI 0.357–5.698, *p* = 0.6150), attitudes towards HIV patients (hard to say OR: 3.487, 95% CI 0.759–16.021, *p* = 0.1084; did not discriminate OR: 4.646, 95% CI 1.208–17.860, *p* = 0.0254), detection of HIV (OR: 2.361, 95% CI 1.118–4.986, *p* = 0.0243), PrEP promotion among MSM (definitely OR: 51.365, 95% CI 5.184–508.960, *p* = 0.0008; hard to say OR: 8.985, 95% CI 0.836–96.509, *p* = 0.0699), advising friends to use PrEP (definitely OR: 30.882, 95% CI 2.121–449.700, *p* = 0.0121; not clear OR: 2.529, 95% CI 0.167–38.315, *p* = 0.5034)
154	Low awareness of and willingness to use PrEP in the Chinese YMSM: an alert in YMSM HIV prevention	Zhuang Cui et al., 2020	2018	China	Cross‐sectional study	495 YMSM	Median age = 22 years, IQR: 20–23	26.1% (129/495)	In those with previous knowledge of PrEP, 27.9% (36/129) conveyed their willingness to use it	YMSM having a higher education level (OR = 2.992, 95% CI: 1.189–7.526) or involved in internet‐based partner‐seeking (OR = 13.993, 95% CI: 3.175–61.662) were more likely to report willingness to use PrEP. YMSM having high HIV/AIDS knowledge (OR = 0.235, 95% CI: 0.069–0.799), frequent condom use (OR = 0.357, 95% CI: 0.161–0.793) and condom promotion and provision/AIDS counselling experience (OR = 0.329, 95% CI: 0.113–0.959) were less willing to use PrEP
155	Prevalence of actual uptake and willingness to use pre‐exposure prophylaxis to prevent HIV acquisition among men who have sex with men in Hong Kong, China	Zixin Wang et al., 2018	Not reported	Hong Kong, China	Cross‐sectional study	403 MSM^64^	Majority of the participants were 18–30 years old (66.0%), were currently single (77.9%), had attained college education or above (80.6%), had a full‐time job (77.7%) and identified themselves as homosexuals (87.6%)	Not reported	45.20%	In the univariate analysis, men who self‐identified as bisexual had higher odds of being willing to use daily oral PrEP at 8000 HKD/month compared to those who self‐identified as homosexual (OR: 2.73, 95% CI: 1.15–6.50; *p*<0.05); men who had anal intercourse with regular male sex partner(s) in the last 6 months had reduced odds of being willing to use daily oral PrEP at 8000 HKD/month
										(OR: 0.39; 95% CI: 0.18–0.84;p<0.05); men who aged 31–35 years old had reduced odds of being willing to use free daily oral PrEP compared to those who aged 15–25 years old (OR: 0.45; 95%CI 0.23–0.87;p<0.05); men who had conducted CAS in the last 6 months with men (OR: 1.64; 95%CI 1.09–2.45;p<0.05) and used sexual potency drugs before/during sexual intercourse (OR: 3.07; 95% CI 1.37–6.89;p<0.001) had higher odds of being willing to use free daily oral PrEP
156	Uptake and willingness to use PrEP among Chinese gay, bisexual and other men who have sex with men with experience of sexualized drug use in the past year	Zixin Wang et al., 2020	2018	Hong Kong, China	Cross‐sectional study	580 MSM^65^	Most participants were aged 18–30 years (56.6%)	Not reported	67.10%	Perceived support from significant others to use PrEP, perceived behavioural control of taking PrEP every day in the next 6 months

^a^ The values of 1 and 2 in a 5‐point Likert scale (1. yes, definitely, 2. yes, probably, 3. not sure, 4. no, probably not and 5. no, definitely not) or 4‐point Likert scale (1. yes, definitely, 2. yes, probably, 3. no, probably not and 4. no, definitely not) were classified into “Yes” for willingness to use PrEP if the article did not provide the dichotomous variable of willingness. The exception is that response categories were dichotomized into 1 category indicating that participants were “highly likely” and another category for all other responses in Article 91 (Lori M. Wardet, 2019).

1. We excluded 26 questionnaires from men who were heterosexual, three from men who had completed the questionnaire previously, 62 from men who had never had sex with a man and 199 from men who did not provide a saliva specimen. As PrEP would only be applicable to men who are HIV negative, we restricted the analyses to 842 men who tested HIV negative, examining attitudes and factors associated with the likelihood of PrEP use with bivariate and multivariate analyses using logistic regression.

2. Between March and June 2019, 413 GBMSM enrolled in the study and completed a quantitative assessment. Inclusion criteria were: (1) 18 years of age or older; (2) currently residing in one of four Nigerian states(Abuja, Lagos, Delta or Plateau); (3) cis‐gender male; and (4) having sex with another male.

3. In all, 246,620 emails were opened, 56,584 individuals clicked the survey link and 36,063 initiated the survey. For these analyses, we limited our sample to respondents who reported: residence in a Spanish‐ or Portuguese‐speaking country in Latin America (including the Caribbean), male gender at birth and current male gender identity, sex with a man in the past year and being HIV uninfected or of unknown HIV status (*n* = 22,698).

4. Of the 5779 men who accessed the survey, 3748 participants met the criteria for inclusion in this analysis.

5. A total of 2455 men took part in the survey, of whom 1911 answered at least one question about PrEP, while the remaining 544 participants stopped immediately after they had chosen a language. Eighteen participants said in a comment that they were already HIV positive and were excluded from the analysis. Ultimately, 1893 participants were included in the analysis.

6. Of 9640 sexually active MSM who consented to an HIV test as part of their participation in NHBS, 6847 (71%) tested negative and were eligible for inclusion in analyses. After excluding those with missing data (231 MSM missing data on PrEP willingness, 131 additional MSM missing data on risks used to assess indications for PrEP and 2 additional MSM missing data on PrEP use), 6483 MSM were left for analysis.

7. 192 HIV‐negative men enrolled in the EleMENt study, 4% (8/192) of men were already taking PrEP upon study entry leaving 184 men eligible for EleMENt's PrEP program.

8. 562 HIV‐negative black and white MSM aged ≥18 years were recruited from the Atlanta community and Facebook. They received a one‐time cross‐sectional survey. A total of 482 MSM completed the PrEP willingness questions querying.

9. The project enrolled both HIV‐negative and HIV‐positive men, though the analyses for this manuscript were limited to HIV‐negative men. Of the 377 men who enrolled in the project, 208 (55.2%) were confirmed to be HIV negative with a rapid HIV antibody test during their assessment. Two of these men were missing responses on key variables for this study; thus, the present analysis focused on the remaining 206 HIV‐negative gay and bisexual men.

10. Of the 376 men who enrolled in the project, 207 (55.2%) were confirmed to be HIV negative with a rapid HIV antibody test during their baseline assessment—two of these participants tested HIV positive at their 12‐month assessment and were excluded. One of these men was missing necessary data at baseline, 42 individuals did not return for their 12‐month assessment and four were missing necessary data at the 12‐month assessment. Thus, analyses focused on a sample of 158 participants.

11. In total, 1208 subjects participated, 342 subjects were excluded for being HIV‐infected or non‐MSM, leaving 866 subjects to be evaluated in this analysis.

12. In the 2‐month recruitment period, 2767 unique individuals consented to the survey, 44% of whom (1225) met the inclusion criteria. Seventy‐nine percent (973) of eligible participants completed the survey. For this analysis, we excluded respondents who reported a gender other than male (*n* = 32), resulting in 1080 responses from cisgender males who completed the survey through initial questions about PrEP, of whom 924 completed the entire survey.

13. We surveyed 491 adolescent men who have sex with men (AMSM) ages 13–18, using forced choice and open‐ended response questions.

14. Of the 221 MSM invited to participate, 21 MSM either declined (*n* = 8), withdrew (*n* = 5) or were ineligible (under 18 years old or not MSM) (*n* = 8), resulting in 200 participants, for a response rate of 90.5%. Given that PrEP is indicated only for persons who are HIV negative, MSM who indicated being HIV positive (*n* = 24) were excluded from analysis.

15. There were 584 participants in the cohort. Of these, 145 participants did not attend at least one follow‐up visit and eight participants did not meet the age criteria. In total, 431 volunteers qualified and were selected. One hundred participants had missing data. Thus, a total of 331 participants were analysed.

16. Excluded men who were HIV positive (*n* = 71), as well as those who had not engaged in sex with another man in the past 6 months (*n* = 43), were currently using PrEP (*n* = 49), or who reported a race/ethnicity other than black (*n* = 15). The final sample consisted of 123 sexually active, self‐reported HIV‐negative BMSM not currently using PrEP.

17. This was an exclusive sample of MSM who were also sex workers.

18. A total of eight FGDs (focus group discussions) with 12 participants per group were conducted making a total of 96 MSM participating in the FGDs and 13 IDIs (in‐depth interviews) were conducted.

19. Among the 282 participants, 215 (76%) self‐reported that they were HIV uninfected and were asked questions about PrEP.

20. A sample of 226 YMSM enrolled in the study, of whom eight (3.5% of whole sample) reported being HIV positive, leaving 218 included in this analysis.

21. A total of 159 participants were enrolled in the study. Seven were deleted because of not having sex with men in the past 6 months, by self‐reports. Finally, 152 were used for the analyses.

22. Overall, 927 MSM enrolled in the study; 65% were HIV uninfected and 35% were HIV infected.

23. Of 3868 participants who expressed interest in the survey, 3842 were consented and screened for eligibility. Only 1777 met inclusion criteria and 762 went on to complete the entire survey. After removing one duplicate survey (determined by IP address), 761 participants remained, of whom 687 had never taken PrEP and were included in our sample.

24. A total of 2325 eligible men were approached and 1515 participated in the survey (65.2% response rate [RR]). Ultimately, a sample of 1393 men was analysed.

25. A total of 66 men were screened and 44 participants were enrolled into the study. Of these, four did not complete all their study visits. As a result, these participants were excluded from our analysis and this brought us to a total analytical sample of 40.

26. Men who were HIV positive (*n* = 75) were excluded from this analysis as were men with missing data on any of the regression variables (*n* = 561) leaving a sample size of 690 participants.

27. Men with missing data on any of the variables in the final regression models were excluded from each analysis, leaving a total of *n* = 356 men for PrEP awareness and *n* = 386 for PrEP acceptability.

28. Four time periods included: April–June 2013 (*n* = 436); May–August 2014 (*n* = 400); November 2014–April 2015 (*n* = 420); and May–August 2016 (*n* = 400).

29. Our sample consisted of GBMSM who were HIV negative (*n* = 5919) or had an unknown HIV status (*n* = 1257), for a total of 7176 respondents living in Canada.

30. In total, 1071 participants joined the study. Approximately 6 months after their baseline assessment, participants were sent an email with a link to verify their contact information had not changed. They were also invited to complete a brief (*10 min) survey about PrEP. As an incentive, participants were offered entry into a raffle for one of 50 Amazon gift cards for $20. In total, 950 (88.7%) participants completed this survey; however, we excluded data from two men who indicated they had been diagnosed with HIV since baseline. Thus, the analytic sample for the current study is 948.

31. Of the 1071 men who enrolled, five men (0.5%) reported an HIV diagnosis in the year since baseline and were excluded. Of the remaining 1066, 1013 (95.0%) completed the 12‐month survey. Men who had formerly been but were no longer prescribed PrEP (*n* = 18) were asked different questions and were not included within the present analyses, resulting in a final analytic sample of 995 HIV‐negative GBM.

32. *N* = 209 study participants out of a total sample of *N* = 549 (38%) tested positive for one or more STIs during at least one of their study appointments. For the present study, only the participants who tested positive for at least one STI at their study appointment were included in our analyses.

33. 1407 MSM were approached, but only 1323 questionnaires completed and analysed.

34. Seven hundred men participated in the survey, but this analysis is limited to the 480 men who reported having sex with a man in the preceding 6 months. Information about mode of survey administration is missing for 23 men; however, these men were included in analyses if they had valid data on other variables. None of the cases out of the 480 were systematically excluded.

35. The analysis for this study was restricted to participants who had reported any history of incarceration at baseline.

36. Of the 200 men enrolled in this study, 129 were recruited from the ongoing prospective cohort study, while 71 were recruited from the community through peer networks.

37. A total of 555 HIV‐seronegative MSM presented to the STI clinic and received PrEP education between August 2014 and December 2016. MSM who were identified as HIV positive at the time of clinical visit were excluded from PrEP education sessions and from this analysis. Of these HIV‐negative MSM, a total of 401 MSM (72%) were tested for rectal STIs and were included in this analysis.

38. Of the 969 men interviewed, 27 minors and 76 HIV‐positive men were excluded leaving a total sample of 866 men included in the final analysis.

39. Participants were 699 men surveyed at the Atlanta Black Gay Pride Festival that occurred in August 2012. Given our focus on factors associated with willingness to use PrEP among BMSM, we removed men reporting: (1) heterosexuality and not reporting male sex partners (*n* = 129), (2) reporting race other than African‐American (*n* = 15) or (3) reporting being HIV positive (*n* = 157). All remaining analysis included 398 HIV‐negative BMSM.

40. In 2011, the survey was completed by 1283 men, of whom 919 self‐reported that they were HIV negative, 122 HIV positive and 242 untested. In 2013, the survey was completed by 1316 men, of whom 966 were HIV negative, 93 HIV positive and 257 untested. In 2015, the first page of the survey was viewed 2451 times and 1795 eligible people commenced the survey (73% participation rate). The survey was completed by 1251 men (70% completion rate), of whom 990 were HIV negative, 106 HIV positive and 155 untested.

41. A total of 3842 individuals took the screener to assess survey eligibility. Of those who were screened, 1777 (46%) met the inclusion criteria to complete the survey. The removal of incomplete and duplicate surveys left a total of 761 unique participants. Of the original sample of 761 participants, 687 (90.3%) had never taken PrEP, and 265 (38.6%) of those who had complete responses were either ambivalent about trying or unwilling to try daily oral PrEP (subsequently referred to as ‘‘unwilling’’). A sub‐sample who stated that they were either ambivalent about trying or unwilling to try daily oral PrEP (*n* = 265) was analysed in this study.

42. During the survey period, 1422 participants entered the survey, and 1231 (86.6%) completed the online questionnaires. Participants who were female (*n* = 19), younger than 18 years of age (*n* = 19), residents of other countries (*n* = 6) or HIV positive (*n* = 55) were excluded. A total of 1151 participants provided data for further analysis.

43. Of the 527 MSM who completed the questionnaire, a total of 443 respondents (i.e. study population) reported being either ‘‘HIV negative without any doubt’’ (66%) or ‘‘probably HIV negative’’ (34%).

44. The analytical sample was restricted to 444 participants (76.6%) who answered negative to HIV status.

45. Analysing data on trends in elements of the PrEP continuum (awareness, willingness and use of PrEP) in a sample of 37,476 HIV‐negative/unknown status MSM from December 2013 through November 2017. Overall, 51.4% (19,244/37,476) of MSM participants in AMIS were also PrEP eligible.

46. Of the 470 respondents who completed the survey, 404 (86%) answered all covariates of interest and were included in the analyses.

47. Data for this analysis are from the Military Acceptance Project (MAP), a two‐phase mixed‐methods study conducted to better understand the integration, acceptance and wellbeing of lesbian, gay, bisexual and transgender (LGBT) service members in the U.S. military.

48. Of the 1339 respondents who commenced the survey, 10 responses were excluded as they did not meet the age requirements for the survey. Another 167 respondents ceased participation immediately after choosing the language they preferred to take the survey in. Another 64 respondents ceased participation midway through the first few questions on demographic attributes. Of the remaining 1162 respondents, 1098 of those who indicated their knowledge and use of PrEP were included in the analytic sample.

49. The participating centres handed out 875 questionnaires, of which 473 were returned, yielding a response rate of 54.1%. We excluded three participants because they had indicated in the questionnaire that they were living with HIV. This left 470 questionnaires for further analysis.

50. Young men were eligible if they had sex with a man in the past 6 months, were 18–39 years of age and identified as being African American or black.

51. Of the 440 men surveyed, 114 (25.9%) self‐reported being HIV positive. Of those who reported being HIV negative or of unknown sero‐status (326, 74%), 72 (22%) tested HIV positive during the rapid test. Thus, in total, 186 men (42.0%) self‐reported or tested HIV positive. Current analysis was restricted to those who self‐reported/tested HIV sero‐negative (*n* = 254).

52. The analysis of perceived likelihood of future PrEP use and perceived ability to adhere to PrEP was further restricted to those who had never used PrEP: 556 participants (72.7%) reported never using PrEP, resulting in 278 couples (556 individuals).

53. Between March 2012 and February 2013, 428 individuals were screened for the study. They had learned about the study from a variety of referral sources: friends (*n* = 133); weekly internet postings on Craigslist.org (*n* = 100); study flyer (*n* = 92); text messages from a community‐based organization serving BMSM (*n* = 46); referred by a house father from the house and ball community (*n* = 27); community presentations (*n* = 16) and other sources (*n* = 14). From those persons screened, 289 individuals were eligible and 224 completed the in‐person study interview.

54. Of the 276 Latino MSM included in the sample, less than 6% (*n* = 16) reported using PrEP.

55. From May 2015 to December 2015, we gathered a convenience sample of 659 HIV‐negative MSM living in Switzerland. Participants who reported being HIV positive or of unknown HIV status, those who were not living in Switzerland, did not answer the question about the intention to use PrEP or did not give their consent were excluded from the sample. In total, 556 participants were included into the final analyses.

56. 2664 participants entered the survey and 1187 (44.6%) consented and completed the questionnaire. Of 1187 men, 1084 identified themselves as Malaysian citizens. The sample was further limited to 992 men who reported to be HIV negative or of unknown status. Of the 992 men, two were excluded because they were under the age of 18.

57. Of the 99,694 emails that were opened, 15,405 individuals clicked through to the survey (15.5%), and 9179 (59.6%) started the survey. Of those who started the survey, a total of 7305 (79.6%) respondents completed all questions without missing data, representing the analytic sample.

58. Of the 1487 attendees approached by interviewers, 592 were screened for eligibility and 498 (33%) were eligible and agreed to participate. For this analysis, we restricted our sample to self‐reported HIV‐negative men and excluded 97 (19%) respondents with a previous positive or indeterminate HIV test resulting in a sample size of 399 participants.

59. In 2014 and 2017, 18,610 sexually active MSM were interviewed (9640 in 2014; 8970 in 2017) in the 20 urban areas. Of those, this analysis is limited to 7873 MSM (42%) who had a negative HIV test result but were at risk for HIV infection and likely met the clinical indications for PrEP (3821 [40%] in 2014; 4052 [45%] in 2017).

60. Of the 459 complete responses collected (completion rate = 71%, of 647 nonduplicate responses collected), six self‐reporting HIV‐positive MSM were excluded. Data from the remaining 453 participants were available for analysis.

61. Of the 1602 MSM who participated in the 2017 NHBS survey in Seattle, WA; Portland, OR; and Denver, CO, 1135 (71%) were HIV‐negative men who reported not being in a monogamous relationship with an HIV‐negative man and oral or anal sex with a male partner in the past 12 months. Among these 1135 participants, we excluded 254 participants who did not have a bacterial STI or CAS in the past 12 months, remaining 881 participants.

62. 887 men who were older than 18 years old and used to have sex with men were recruited through internet between 27 November and 17 December 2013. Totally 760 qualified questionnaires were collected.

63. Overall, 70.2% (541 out of 771) of MSM reported having a basic or a high level of PrEP comprehension, and 541 MSM were included in this analysis.

64. A total of 567 eligible MSM were approached through outreach in gay venues (*n* = 323), online recruitment (*n* = 60) and peer referral (*n* = 184), and 403 of them (71.1%) provided verbal consent and completed the interview (venue: 232, online: 60 and referral: 111).

65. Out of 1131 prospective participants being approached through outreach in gay venues (*n* = 211), online recruitment (*n* = 607) and peer referral (*n* = 313), 906 showed interest to join the study and left their contact information. All these 906 participants were successfully contacted, 711 were screened to be eligible. Of eligible participants, 600 provided verbal informed consent and completed the telephone interview. The main reason for not providing informed consent was lack of time to complete the survey (*n* = 71), while the other 40 refusals did not specify their reason. This study was based on 580 GBMSM self‐reported to be HIV‐negative/unknown HIV sero‐status.

**Table A2:** Factors associated with willingness to use PrEP among MSM

	Factors	References	OR 95% CI	Author
PrEP awareness	Has heard of PrEP	Not heard of PrEP	5.63 (1.21–26.19)	Marieke Bak et al.
	Heard of PrEP	Not heard of PrEP	1.38 (1.07–1.76)	Ingrid Young et al.
	Understand PrEP medication	Not understand PrEP medication	1.98 (1.36–2.88)	Xue et al.
	Heard of PrEP	Not heard of PrEP	1.7 (1.4–2.2)	Jing Han et al., 2019
	Heard of PrEP	Not heard of PrEP	2.81 (1.64–4.82)	Lisa A. Eaton et al., 2017
	Heard of PrEP	Not heard of PrEP	1.40 (1.06–1.86)	Sin How Lim et al., 2017
	Having previously heard of PrEP	Not having previously heard of PrEP	1.33 (1.01–1.75)	Yan Zhang et al., 2013
	PrEP awareness	No PrEP awareness	1.76 (1.31–2.38)	Hoagland et al., 2016
	PrEP awareness	No PrEP awareness	1.48 (1.3–1.7)	Thiago Silva Torres et al., 2018
	PrEP knowledge	No PrEP knowledge	1.96 (1.05–3.69)	Mart van Dijk et al., 2020
	Heard of PrEP	Not heard of PrEP	1.03 (0.74–1.42)	Jamie Frankis et al., 2016
	Aware of PrEP	No PrEP awareness	1.35 (0.72–2.53)	Susan Fallon, 2015
	Heard of PrEP	Not heard of PrEP	1.63 (0.99–2.68)	Liping Peng et al., 2019
	Aware of PrEP	No PrEP awareness	2.71 (1.27–5.77)	Janneke P. Bil et al., 2015
	Aware of PrEP	Unaware of PrEP	0.65 (0.35–1.21)	Adedotun Ogunbajo et al., 2019
	Heard of PrEP	No	3.07 (2.09–4.51)	Weiying Chen et al., 2021
Pooled OR			1.62 (1.39–1.88)	
*Condomless sexual behaviours*	Had any higher risk UAI	No high‐risk UAI	2.14 (1.46–3.15)	Ingrid Young et al., 2013
	Had unprotected anal sex in the last 12 months	No unprotected anal sex in the last 12 months	1.4 (1.1–1.6)	Jing Han et al., 2019
	Condomless receptive anal intercourse	No condomless receptive anal intercourse	1.27 (1.12–1.44)	Thiago Silva Torres et al., 2018
	Not used a condom the last time	Used a condom the last time	2.00 (1.17–3.42)	Mart van Dijk et al., 2020
	Risk behaviour	No risk behaviour	2.9 (2.14–3.90)	Christoph D. Spinner et al., 2018
	Condomless anal sex, past 12 months	No condomless anal sex, past 12 months	1.47 (1.28–1.70)	Jingjing Li et al., 2018
	Any CAS reported	No CAS reported	2.21 (1.25–3.91)	Christian Grov et al., 2016
	Had any higher risk UAI	No high‐risk UAI	2.27 (1.37–3.78)	Jamie Frankis et al., 2016
	UAI with casual partners in the last year	Not had UAI with casual partners in the last year	1.70 (1.13–2.56)	Adamma Aghaizu et al., 2012
	Any UAI (12 months)	No UAI (12 months)	1.73 (1.14–2.61)	Charlotte‐Paige Rolle et al., 2018
	High‐risk sex – prior 3 months	No high‐risk sex prior 3 months	1.72 (1.45–2.03)	Douglas S. Krakower et al., 2012
	Any UAI in the past 12 months	No UAI in the past 12 months	1.09 (0.73–1.64)	Susan Fallon, 2015
	Unprotected receptive anal sex at the last sex	No unprotected receptive anal sex at the last sex	1.20 (0.71–2.03)	Adams J. W. et al., 2016
	Had receptive condomless anal sex with a man in last 6 months	Not had receptive condomless anal sex with a man in the last 6 months	1.32 (0.91–1.93)	Ian W. Holloway et al., 2017
	UAI with an HIV+ or unknown status sex partner	No UAI with an HIV+ or unknown status sex partner	1.76 (0.84–3.68)	Elizabeth Anne Barash et al., 2010
	Condomless receptive anal sex in the past 12 months	No condomless receptive anal sex or no sex in the past 12 months	1.14 (0.67–1.95)	Tonia Poteat et al., 2020
	Condomless anal sex, last 6 months	No condomless anal sex, last 6 months	1.16 (0.57–2.38)	Li Yan et al., 2020
	Condom use in the last 6 months	No condom use in the last 6 months	4.80 (1.43–16.09)	Rissa Cempaka et al., 2020
Pooled OR			1.62 (1.42–1.84)	
*STIs/HIV positive*	Had STI in the previous 12 months	No STI in the previous 12 months	1.12 (0.77–1.63)	Ingrid Young et al., 2013
	Had STI history	No STI history	1.61 (1.09–2.39)	Yan Zhang et al., 2013
	STD diagnosis in the last 12 months	No STD diagnosis in the last 12 months	1.92 (1.14–3.26)	Hoagland et al., 2016
	Sexually transmitted infection diagnosis	No sexually transmitted infection diagnosis	1.25 (1.03–1.51)	Thiago Silva Torres et al., 2018
	STI in in the past 12 months	No STI in the past 12 months	1.53 (0.76–3.08)	Mart van Dijk et al., 2020
	Had history of STDs	No STDs history	1.85 (1.17–2.91)	Christoph D. Spinner et al., 2018
	Any bacterial STIs, past 12 months	No bacterial STIs, past 12 months	4.15 (2.93–5.88)	Jingjing Li et al., 2018
	Had an STI in the previous 12 months	No STI in the previous 12 months	1.4 (0.84–2.34)	Jamie Frankis et al., 2016
	STI in the last year	No STI	1.00 (0.68–1.47)	Adamma Aghaizu et al., 2012
	HIV positive	HIV negative	3.27 (1.89–5.63)	Martin Holt et al., 2017
	HIV/STI incidence	No HIV/STI incidence	1.46 (0.99–2.15)	Charlotte‐Paige Rolle et al., 2018
	STI history (ever)	No STI history	4.63 (1.70–12.60)	Daniel Yang et al., 2013
	Diagnosis with STD in the past 12 months	No STD diagnosis in the last 12 months	1.23 (0.64–2.36)	Susan Fallon, 2015
	Newly identified as HIV infected during NHBS study visit	Not identified as HIV infected during NHBS study visit	4.83 (1.03–22.67)	Matthew E. Levy et al., 2017
	Had STI history	No STI history	1.52 (0.76–3.07)	Tonia Poteat et al., 2020
Pooled OR			1.74 (1.36–2.23)	
*STIs/HIV test history*	Had an HIV or STI test in the previous 12 months	No HIV/STIs test in the previous 12 months	1.38 (1.09–1.76)	Ingrid Young et al., 2013
	Prior HIV test in the last 12 months	No prior HIV test in the last 12 months	2.07 (1.53–2.79)	Hoagland et al., 2016
	Had an STI test in the previous 12 months	No STI test in the previous 12 months	1.18 (0.88–1.6)	Jamie Frankis et al., 2016
	Last HIV test in the last year	Not had HIV test in the last year	0.98 (0.64–1.53)	Adamma Aghaizu et al., 2012
	HIV test (12 months)	No HIV test (12 months)	0.84 (0.53–1.32)	Charlotte‐Paige Rolle et al., 2018
	HIV testing (ever)	No HIV testing	2.39 (1.07–5.32)	Daniel Yang et al., 2013
	HIV test	No HIV test history	0.65 (0.4–1.06)	Zhong et al., 2013
	Tested for HIV in the past 12 months	No HIV test in the past 12 months	0.81 (0.55–1.22)	Susan Fallon, 2015
	HIV test in prior 12 months	No HIV test in prior 12 months	1.25 (1.13–1.38)	Ashwin Belludi et al., 2021
	Have history of HIV test	No history of HIV test	2.341 (1.552–3.532)	Yuansheng Fu et al., 2021
	Recent STI testing	No recent STI testing	1.58 (1.01–2.48)	Kevin J. Blair et al., 2021
Pooled OR			1.28 (1.05–1.55)	
*Perceived risk of HIV infection*	Perceived high risk of HIV	Perceived risk of HIV	3.33 (2.5–3.33)	Jing Han et al., 2019
	Perceived likelihood of contracting HIV in the next 12 months	Not perceived likelihood of contracting HIV in the next 12 months	0.80 (0.55–1.16)	Sin How Lim et al., 2017
	Perceived likelihood of getting HIV in the next 12 months (high)	Perceived likelihood of getting HIV in the next 12 months (low)	1.42 (1.00–2.02)	Hoagland et al., 2016
	High and unknown perceived likelihood of getting HIV in the next year	Low perceived likelihood of getting HIV in the next year	1.72 (1.47–2.02)	Thiago Silva Torres et al., 2018
	Perceived risk of HIV infection in the next 12 months	Not perceived risk of HIV infection in the next 12 months	3.33 (1.20–9.10)	Xie et al., 2017
	Self‐perceived risk of HIV acquisition	Self‐perceived no risk of HIV acquisition	1.20 (1.13–1.27)	Douglas S. Krakower et al., 2012
	High risk of HIV infection	Low risk of HIV infection	0.70 (0.34–1.54)	Zhong et al., 2013
	High concern for getting HIV	Low concern for getting HIV	1.84 (1.13–3.01)	Ian W. Holloway et al., 2017
	Perceives moderate‐to‐high HIV risk	Perceives low HIV risk	3.4 (2.0–5.8)	James Wilton et al., 2015
	Low risk Moderate to high risk	No risk No risk	3.97 (2.39–6.60) 7.52 (3.52–16.08)	Tobias Herder et al., 2020
	HIV risk perception	No perceived HIV risk	0.91 (0.39–2.12)	Tonia Poteat et al., 2020
Pooled OR			1.74 (1.23–2.45)	
*PrEP efficacy*	Doubts on PrEP's efficacy	No doubt on PrEP efficacy	0.8 (0.7–1.0)	Jing Han et al., 2019
	Not believing that PrEP was effective in preventing HIV	Believing that PrEP was effective in preventing HIV	0.68 (0.55–0.86)	Yan Zhang et al., 2013
	High perception of worry about PrEP efficacy	No worries about PrEP's efficacy	0.70 (0.23–2.09)	Janneke P. Bil et al., 2015
Pooled OR			0.74 (0.65–0.85)	
*Side effects*	Worry about PrEP's side effects	No worries about PrEP's side effects	0.9 (0.7–1.1)	Jing Han et al., 2019
	High perception of worry about PrEP side effects	No worries about PrEP's side effects	0.22 (0.07–0.74)	Janneke P. Bil et al., 2015
	Heard of the side effects of antiretroviral drugs	Do not heard of the side effects of antiretroviral drugs	0.30 (0.14–0.67)	Feng Zhou et al., 2012
	Concerned about PrEP side effects	Not concerned about PrEP side effects	0.35 (0.21–0.59)	Bridget L. Draper et al., 2017
Pooled OR			0.703 (0.577–0.856)	
*Daily medication*	Inconvenience in taking PrEP everyday	Convenience in taking PrEP everyday	1.0 (0.8–1.3)	Jing Han et al., 2019
	Take PrEP every day has been a barrier to PrEP use	Do not think PrEP every day has been a barrier to PrEP use	1.49 (0.19–12.0)	Steven A. Elsesser et al., 2015
Pooled OR			1.005 (0.790–1.280)	
*Condom preference*	Prefer using condom as protection for HIV	Do not want to use condom	0.5 (0.4–0.5)	Jing Han et al., 2019
	Willingness to use condoms	Unwillingness to use condoms	0.86 (0.75–1)	Thiago Silva Torres et al., 2018
Pooled OR			0.65 (0.39–1.11)	
*Sexual orientation*	Bisexual	Gay	0.52 (0.09–3.16)	Lisa A. Eaton et al., 2017
	Bisexual	Gay	1.06 (0.73–1.52)	Sin How Lim et al., 2017
	Bisexual	Gay	1.25 (0.85–1.85)	Jamie Frankis et al., 2016
	Bisexual	Gay	1.76 (0.96–2.23)	Susan Fallon, 2015
	Bisexual	Homosexual	0.9 (0.7–1.1)	Jing Han et al., 2019
	Bisexual	Homosexual	0.99 (0.98–1.00)	B. Lebouché et al., 2015
	Bisexual	Homosexual	0.22 (0.06–0.82)	Xie et al., 2017
	Bisexual	Homosexual	2.85 (1.74–4.64)	Carin Ahouada et al., 2020
	Bisexual	Homosexual	2.22 (1.45–7.69)	Rob Stephenson et al., 2021
Pooled OR			1.22 (0.95–1.57)	
	Heterosexual	Gay	0.36 (0.13–0.97)	Sin How Lim et al., 2017
	Heterosexual	Gay	0.61 (0.21–1.79)	Susan Fallon, 2015
Pooled OR			0.461 (0.221–0.959)	
*Health insurance*	Currently have health insurance	Have no health insurance	0.37 (0.20–0.68)	Lisa A. Eaton et al., 2017
	Current health insurance	No current health insurance	1.08 (0.85–1.36)	Jingjing Li et al., 2018
	Public insurance	No insurance coverage	1.19 (0.75–1.89)	Susan Fallon, 2015
	Private insurance	No insurance coverage	1.64 (0.99–2.78)	Susan Fallon, 2015
	Health insurance status	No health insurance	0.80 (0.43–1.49)	Elizabeth Anne Barash et al., 2010
Pooled OR			1.026 (0.860–1.226)	
*PrEP use*	Ever taken PrEP	No PrEP use	3.71 (1.93–7.14)	Steven A. Elsesser et al., 2015
	Ever used oral PrEP	No oral PrEP use	3.67 (1.20–11.24)	Matthew E. Levy et al., 2017
	History of PrEP use	No history of PrEP use	1.97 (0.74–5.24)	Adedotun Ogunbajo et al., 2019
	Uptake of PrEP before	No uptake of PrEP before	13.31 (5.23–33.90)	Jingzhen Lai et al., 2020
	Ever taken PrEP	PrEP naive	1.67 (1.43–1.96)	Drew A. Westmoreland et al., 2021
	Prior use of PrEP	No prior use of PrEP	6.17 (1.98–27.40)	Shufang Sun et al., 2021
Pooled OR			3.67 (1.84–7.32)	
*PEP awareness*	Heard of PEP	Not heard of PEP	0.656 (0.409–1.053)	Zhong et al., 2013
	Aware of PEP	No aware of PEP	0.49 (0.25–0.96)	Pierre‐julien Coulaud et al., 2018
	Aware of PEP	No aware of PEP	0.51 (0.44–0.61)	Douglas S. Krakower et al., 2012
Pooled OR			0.596 (0.405–0.877)	
				
*PEP use*	Ever taken PEP	No PEP use	2.31 (1.72–3.11)	Martin Holt et al., 2017
	Ever taken PEP	No PEP use	1.62 (1.03–2.56)	Steven A. Elsesser et al., 2015
	Previous PEP use	No previous PEP use	1.94 (1.17–3.24)	Adamma Aghaizu et al., 2012
	Prior use of PEP	No prior PEP use	1.50 (0.998–2.250)	Jayoti Rana et al., 2018
	Prior use of PEP	No prior PEP use	3.7 (1.4–9.6)	James Wilton et al., 2015
Pooled OR			1.959 (1.617–2.372)	
*Whether advise friends to use PrEP*	Willing to introduce PrEP to friends	Not willing to introduce PrEP to friends	94.792 (12.188–737.266)	Zhong et al., 2013
	Advise friends to use PrEP	Do not advise friends to use PrEP	27.006 (13.060–55.843)	Wei et al., 2011
Pooled OR			31.063 (15.661–61.610)	
*Age*				
	≥25 years	<25 years	MSM 2.30 (1.10–4.79) TG 0.81 (0.28–2.36)	Daniel Yang et al., 2013
	≥50 years ≥50 years ≥50 years ≥50 years	<20 years 20–29 years 30–39 years 40–49 years	0.83 0.87 0.86 0.93	B. Hampel et al., 2017
	25–34 years 35–44 years 45+ years	18–24 years 18–24 years 18–24 years	0.82 (0.49–1.38) 0.75 (0.43–1.33) 1.01 (0.59–1.71)	Susan Fallon, 2015
	Age≥40	Age<40	0.51 (0.27–0.94)	Elizabeth Anne Barash et al., 2010
	26–35 years 36–45 years 46+ years	18–25 years 18–25 years 18–25 years	0.58 (0.44–0.76) 0.54 (0.40–0.72) 0.53 (0.38–0.74)	Ingrid Young et al., 2013
	31–49 years ≥50 years	≤30 years ≤30 years	0.98 (0.86–1.12) 0.80 (0.70–0.92)	Jeffrey Morgan et al., 2018
	35+years 35+years	18–24 years 25–34 years	2011 Washington, DC 0.44 (0.21–0.93); Miami 0.53 (0.27–1.05) 2011 Washington, DC 0.6 (0.34–1.06); Miami 0.7 (0.36–1.35)	Rudy Patrick et al., 2017
	≤25 years	>25 years	1.23 (0.78–1.92)	Tsz Ho Kwan et al., 2019
	25–34 ≥35 25–34 ≥35	<25 <25 <25 <25	0.8 (0.4–1.3)^c^ 0.8 (0.4–2.4)^c^ 0.8 (0.4–0.13)^d^ 0.7 (0.4–1.3)^d^	Mao et al., 2017
	>30 >30	< 20 20–30	0.17 (0.047–0.58) 0.49 (0.30–0.81)	Zhong et al., 2013
	18–29 30–39 18–29 30–39 18–29 30–39	≥40 ≥40 ≥40 ≥40 ≥40 ≥40	2.33 (1.28, 4.23)^e^ 2.56 (1.38, 4.75)^e^ 2.15 (1.16, 3.98)^f^ 2.28 (1.21, 4.29)^f^ 2.17 (1.17, 4.03)^g^ 2.30 (1.22, 4.33)^g^	Meagan Zarwell et al., 2019
	18–25 26–30 31–40 >40	<18 <18 <18 <18	0.9 (0.6–1.1) 1.0 (0.7–1.4) 1.2 (0.8–1.7) 2.0 (1.1–3.6)	Jing Han et al., 2019
	26–30 31–35 >35 26–30 31–35 >35	18–25 18–25 18–25 18–25 18–25 18–25	0.97 (0.42–2.24)^a^ 0.83 (0.25–2.69)^a^ 0.36 (0.10–1.33)^a^ 0.64 (0.39–1.03)^b^ 0.45 (0.23–0.87)^b^ 0.59 (0.34–1.03)^b^	Zixin Wang et al., 2018
	26–35 36–45 >45	18–25 18–25 18–25	1.33 (0.71–2.51) 1.25 (0.65–2.41) 1 (0.54–1.85)	Jamie S. Frankis et al., 2015
	25–35 36–45 ≥46	18–25 18–25 18–25	0.61 (0.4–0.95) 0.47 (0.30–0.75) 0.54 (0.35–0.82)	Jamie Frankis et al., 2016
	≥35	<35	0.44 (0.32–0.59)	Adamma Aghaizu et al., 2012
	≥35	<30	2.37 (1.03–5.46)	Feng Zhou et al., 2012
	>25	≤25	0.61 (0.38–1.00)	Liping Peng et al., 2019
	26–35 >35	18–25 18–25	0.85 (0.57, 1.26) 0.79 (0.47, 1.33)	Charlotte‐Paige Rolle et al., 2018
	≥30	18–29	0.44 (0.2–0.94)	Ronald A. Brooks et al., 2015
	25–34 35–44 ≥45	18–24 18–24 18–24	0.70 (0.46–1.06) 0.96 (0.56–1.63) 2.18 (1.13–4.23)	Yingying Ding et al., 2016
	25–29	18–24	0.7 (0.44–1.57)	Ian W. Holloway et al., 2017
	25–34 35–44 45–54 >55	16–24 16–24 16–24 16–24	3.97 (2.19–7.18) 5.23 (2.66–10.27) 4.17 (2.03–8.57) 2.06 (0.87–4.88)	Darcy White Rao et al., 2019
	25–34 ≥35	18–24 18–24	1.08 (0.85–1.36) 1.15 (0.83–1.59)	Yan Zhang et al., 2013
	46–65 46–65	18–25 26–35	0.78 (0.23–3.63) 0.39 (0.12–1.25)	Ronald A. Brooks et al., 2019
	25–35 ≥36	18–24 18–24	1.03 (0.73–1.46) 1.23 (0.80–1.87)	Hoagland et al., 2016
	25–35 36–45 46+	16–24 16–24 16–24	0.85 (0.73–0.98) 0.79 (0.66–0.94) 0.65 (0.49–0.85)	Drew A. Westmoreland et al., 2021
	25–34 35 +	18–24 18–24	1.54 (1.10–1.65) 2.23 (1.45–3.64)	Rob Stephenson et al., 2021
	>23 to ≤26 >26 to ≤29 >29	≤23 ≤23 ≤23	1.68 (0.54–5.26) 0.58 (0.23–1.48) 0.77 (0.29–2.08)	Athanase Munyaneza et al., 2021
*Sexual partnership*				
	Number of male sexual partners >20 in the previous 12 months	Number of male sexual partners ≤20 in the previous 12 months	1.71 (1.09–2.69)	Nicolas Lorente et al., 2011
	One regular partner in the preceding 6 months ≥2 regular partners in the preceding months	Zero regular partners in the preceding 6 months; Zero regular partners in the preceding 6 months	MSM 0.40 (0.15–1.08); TG 1.27 (0.43–3.89); MSM 0.49 (0.19–1.26); TG 1.26 (0.38–4.28)	Daniel Yang et al., 2013
	1–2 sex partners	≥3 sex partners	1.18 (0.64–2.21)	Elizabeth Anne Barash et al., 2010
	Only one male sexual partner in the last 12 months 2–5 male sexual partners in the last 12 months	6+ male sexual partners in the last 12 months; 6+ male sexual partners in the last 12 months;	2011 Washington, DC 0.75 (0.36–1.55) Miami 0.87 (0.42–1.79); 2014 Washington, DC 0.38 (0.18–0.80) Miami 0.70 (0.39–1.24) 2011 Washington, DC 0.55 (0.31–0.95) Miami 0.79 (0.45–1.40); 2014 Washington, DC 0.45 (0.27–0.75) Miami 0.69 (0.44–1.08);	Rudy Patrick et al., 2017
	>2 male sex partner in the past 6 months >2 male sex partner in the past 6 months	≤2 male sex partners in the past 6 months ≤2 male sex partners in the past 6 months	1.7 (1.1–2.7) 1.8 (1.1–2.9)	Mao et al., 2017
	≥10 sex partners ≥10 anal sex partners	<10 sex partners <10 anal sex partners	1.13 (0.71–1.79) 1.3 (0.84–2.01)	Jamie S. Frankis et al., 2015
	≥10 AI partners in the past year	<10 AI partners in the last year	2.40 (1.77–3.50)	Adamma Aghaizu et al., 2012
	Total number of MSM friends ≥10	Total number of MSM friends <10	1.43 (0.69–2.96)	Feng Zhou et al., 2012
	2–4 male sex partners in the past 12 months 5–7 male sex partners in the past 12 months 8 or more male sex partners in the past 12 months	1 male sex partner in the past 12 months 1 male sex partner in the past 12 months 1 male sex partner in the past 12 months	1.2 (1.1–1.3) 1.3 (1.2–1.4) 1.4 (1.3–1.5)	Brooke E. Hoots et al., 2016
	1 regular sexual partner in the past 6 months >1 regular sexual partner in the past 6 months 1–2 non‐regular sexual partners in the past 6 months 3–5 non‐regular sexual partners in the past 6 months >5 non‐regular sexual partners in the past 6 months	0 regular sexual partner in the past 6 months 0 regular sexual partner in the past 6 months 0 non‐regular sexual partner in the past 6 months 0 non‐regular sexual partner in the past 6 months 0 non‐regular sexual partner in the past 6 months	0.87 (0.49–1.55) 1.43 (0.68–3.00) 2.55 (1.45–4.48) 2.85 (1.37–5.93) 3.36 (1.10–10.26)	Liping Peng et al., 2019
	2–10 male sex partners in the last 6 months >10 male sex partners in the last 6 months	0–1 male sex partner in the last 6 months 0–1 male sex partner in the last 6 months	2.00 (1.28–3.14) 2.73 (1.59–4.69)	Martin Holt et al., 2017
	1–2 UAI partners (12 months) ≥3 UAI partners (12 months)	0 UAI partner (12 months) 0 UAI partner (12 months)	1.45 (0.94, 2.25) 2.34 (1.40, 3.91)	Charlotte‐Paige Rolle et al., 2018
	1 male anal sex partner ≥2 male anal sex partners	0 male anal sex partner 0 male anal sex partner	1.18 (0.71, 1.97) 1.98 (1.29, 3.05)	Sin How Lim et al., 2017
	5–10 partners in the past 3 months >10 partners in the past 3 months	0–4 partners in the past 3 months 0–4 partners in the past 3 months	1.26 (0.89–1.79) 1.73 (1.17–2.55)	B. Lebouché et al., 2015
	2–10 male sex partners in lifetime ≥11 male sex partners in lifetime 2–5 male anal sex partners in the past 6 months ≥6 male anal sex partners in the past 6 months	1 male sex partner in lifetime 1 male sex partner in lifetime 0–1 male anal sex partner in the past 6 months 0–1 male anal sex partner in the past 6 months	0.79 (0.39–1.59) 1.30 (0.93–1.81) 1.53 (1.07–2.17) 1.82 (1.05–3.17)	Yingying Ding et al., 2016
	2–5 6–10 More than 10	0–1 0–1 0–1	3.40 (0.68–16.99) 7.90 (1.54–55.69) 10.24 (1.88–55.78)	Ronald A. Brooks et al., 2019
	2 or more male condomless anal sexual partners in the 12 months	Less than 2 male condomless anal sexual partners in the 12 months	2.07 (1.47–2.91)	Hoagland et al., 2016
	≥2 sex partners in the past 6 months	<2 sex partners in the past 6 months	0.95 (0.76–1.19)	Yan Zhang et al., 2013
	5 or more of male partners in prior 6 months	One of male partners in prior 6 months	1.33 (1.17–1.51)	Ashwin Belludi et al., 2021
	Main male partner	No main male partner	1.78 (1.55–2.04)	Ashwin Belludi et al., 2021
	>1 MSM/waria sex partner in the last 6 months	≤1 MSM/waria sex partner in the last 6 months	4.26 (1.26–14.45)	Rissa Cempaka et al., 2020
	1 rCLAI 2–4 rCLAI male partners ≥5 rCLAI male partners	0 rCLAI male partner 0 rCLAI male partner 0 rCLAI male partner	1.41 (0.86–2.32) 3.26 (1.83–5.81) 4.63 (2.04–10.52)	Tobias Herder et al., 2020
	1 ≥ 2	0 0	3.56 (1.68–7.54) 2.53 (1.24–5.15)	Li Yan et al., 2020
	1 2–4 ≥5	0 0 0	0.58 (0.21–1.60) 1.11 (0.40–3.10) 1.15 (0.33–3.97)	Athanase Munyaneza et al., 2021
*Education degree*				
	≥5 years of university education or the equivalent	<5 years of university education or the equivalent;	0.57 (0.39–0.84)	Nicolas Lorente et al., 2011
	Some college and above	Less than college	Injectable PrEP 2.92 (1.32–6.46); on‐demand PrEP 2.28 (1.06–4.90); either method 5.54 (1.78–17.22)	Matthew R. Beymer et al., 2018
	Some college or more	High school or less	0.83 (0.50–1.35)	Susan Fallon, 2015
	Degree/postgraduate Degree/postgraduate	Secondary Further/vocational	0.61 (0.44–0.85) 0.57 (0.45–0.72)	Ingrid Young et al., 2013
	High school College/some university Undergraduate degree Graduate degree	Some high school Some high school Some high school Some high school	1.25 (0.92–1.69) 1.03 (0.78–1.36) 0.83 (0.62–1.11) 0.70 (0.52–0.95)	Jeffrey Morgan et al., 2018
	University or more	High school or less	0.52 (0.27–0.99)	Carlos Iniesta et al., 2018
	Attained postsecondary or above education level	Below postsecondary degree	1.64 (0.95–2.83)	Tsz Ho Kwan et al., 2019
	At least a college degree	Less than college degree	0.40 (0.21–0.77)	Christian Grov et al., 2016
	Education beyond high school	Less than a high school education	Age 22 years or younger 2.42 (1.06–5.55); age 23 years or older 0.66 (0.25–1.77)	Lori M. Wardet, 2019
	High school College or more High school College or more	Middle school or less Middle school or less Middle school or less Middle school or less	0.8 (0.4–1.6) 0.9 (0.5–1.5) 1.1 (0.6–1.2) 0.8 (0.5–1.3)	Mao et al., 2017
	Some college College graduate	High school or less High school or less	2.38 (1.19–4.76) 2.01 (1.06–3.82)	Meagan Zarwell et al., 2019
	College or above College or above	Secondary or below Secondary or below	1.68 (0.57–4.94)^a^ 1.15 (0.70–1.90)^b^	Zixin Wang et al., 2018
	Further/vocational Degree/postgraduate	Secondary/none Secondary/none	1.42 (0.6–3.37) 1.67 (0.78–3.59)	Jamie S. Frankis et al., 2015
	College/university	High school	0.96 (0.45–1.09)	Jayoti Rana et al., 2018
	Further Degree	Secondary Secondary	0.93 (0.54–1.60) 0.93 (0.58–1.49)	Jamie Frankis et al., 2016
	Years of education ≥12	Years of education <12	0.53 (0.26–1.12)	Feng Zhou et al., 2012
	More than high school	High school or less	1.0 (0.9–1.0)	Brooke E. Hoots et al., 2016
	Trade certificate University degree	Up to year 12 Up to year 12	0.95 (0.66–1.38) 1.71 (1.23–2.37)	Martin Holt et al., 2017
	<College College+	≤HS ≤HS	1.01 (0.59–1.74) 0.74 (0.44–1.25)	Charlotte‐Paige Rolle et al., 2018
	Higher than secondary school Missing data	Secondary school or less Secondary school or less	0.58 (0.34–0.994) 0.86 (0.43–1.71)	Pierre‐julien Coulaud et al., 2018
	High school or equal College or above	Middle school or below Middle school or below	0.83 (0.48–1.43) 0.76 (0.43–1.31)	Yingying Ding et al., 2016
	Completed part time Some college and above	Less than high school Less than high school	1.35 (0.55–3.30) 1.15 (0.51–2.64)	Ian W. Holloway et al., 2017
	4‐year college or higher Some college/vocational school	High school or less High school or less	3.66 (1.55–8.66) 2.00 (0.82–4.89)	Darcy White Rao et al., 2019
	University degree	No university degree	2.44 (1.22–4.91)	Ricardo Niklas Werner et al., 2018
	College or higher	Senior middle school or lower	0.68 (0.54–0.85)	Yan Zhang et al., 2013
	Bachelor's degree or higher Bachelor's degree or higher	High school/GED or less “Associate/technical degree or some college”	0.3 (0.11–0.81) 0.52 (0.24–1.12)	Ronald A. Brooks et al., 2019
	≥12 years	<12 years	1.52 (1.13–2.06)	Hoagland et al., 2016
	≥ High school	< High school	2.00 (1.17–3.42)	Venkatesan Chakrapani, 2021
	College or above	Middle school or below	1.881 (1.082–3.268)	Yuansheng Fu et al., 2021
	Some college or technical school training College graduate +	High school diploma or GED	0.83 (0.70–0.98) 0.72 (0.60–0.86)	Drew A. Westmoreland et al., 2021
	More than high school	High school or less	0.78 (0.48–1.23)	Shufang Sun et al., 2021
	University and above	Secondary and below	2.12 (1.50–3.48)	Rob Stephenson et al., 2021
	Technical + secondary University	No education + primary	1.06 (0.47–2.41) 1.35 (0.33–5.44)	Athanase Munyaneza et al., 2021
*Income*				
	$20,000–$39,999 $40,000–$59,999 $60,000–$89,999 ≥$90,000	Annual personal income ≤$19,999 Annual personal income ≤$19,999 Annual personal income ≤$19,999 Annual personal income ≤$19,999	1.05 (0.90–1.23) 1.07 (0.91–1.26) 1.09 (0.93–1.29) 0.99 (0.83–1.18)	Jeffrey Morgan et al., 2018
	Annual household income $40,000+ Annual household income $40,000+	$10,000–$19,999 $20,000–$39,999	2011 Washington, DC 0.54 (0.25–1.18); Miami 0.57 (0.31–1.08) 2011 Washington, DC 1.08 (0.53–2.17); Miami 0.54 (0.29–0.99)	Rudy Patrick et al., 2017
	Monthly income ≥HK $15,000	Monthly income <HK $15,000	0.82 (0.52–1.30)	Tsz Ho Kwan et al., 2019
	Annual income>60 000 yuan	Annual income ≤60 000 yuan	1.56 (1.09–2.22)	Xue et al., 2015
	>2000 Yuan >2000 Yuan	≤2000 Yuan ≤2000 Yuan	1.6 (1.0–2.7) 1.9 (1.1–3.2)	Mao et al., 2017
	>5000 Yuan >5000 Yuan >5000 Yuan	≤1000 Yuan 1001–3000 Yuan 3001–5000 Yuan	0.43 (0.17–1.1) 0.6 (0.24–1.47) 1.63 (0.61–4.35)	Zhong et al., 2013
	Annual income 10,001–30,000 Annual income 30,001–150,000 Annual income 150,001 and above	Annual income <10,000 Annual income <10,000 Annual income <10,000	0.9 (0.7–1.2) 1.0 (0.8–1.3) 1.3 (0.8–1.9)	Jing Han et al., 2019
	>2000 Yuan	≤2000 Yuan	0.53 (0.26–1.12)	Feng Zhou et al., 2012
	Annual income $20,000–$39,999 Annual income $40,000–$74,999 Annual income ≥$75,000	Annual income <$20,000 Annual income <$20,000 Annual income <$20,000	1.0 (0.9–1.1) 1.0 (0.9–1.0) 1.0 (0.9–1.0)	Brooke E. Hoots et al., 2016
	>30K per anum	<30K per anum	0.87 (0.63, 1.22)	Bridget L. Draper et al., 2017
	Annual income 20,000–54,999 CAD Annual income ≥55,000 CAD	Annual income <20,000 CAD Annual income <20,000 CAD	1.21 (0.90–1.61) 1.14 (0.83–1.56)	B. Lebouché et al., 2015
	$10,000–$29,999 >$30,000	<$9999 <$9999	0.88 (0.54–1.44) 1.08 (0.61–1.93)	Ian W. Holloway et al., 2017
	$15,001–30,000 More than $30,000	$0–15,000 $0–15,000	0.39 (0.17–0.92) 0.39 (0.14–1.10)	Ronald A. Brooks et al., 2019
	$15,000–$29,999 $30,000–$49,999 $50,000–$99,999 ≥$100,000	<$15,000 <$15,000 <$15,000 <$15,000	2.07 (0.73–5.86) 2.80 (1.09–7.14) 2.37 (0.96–5.86) 2.90 (1.13–7.46)	Darcy White Rao et al., 2019
	1000–3000 yuan/month >3000	<1000 yuan/month <1000 yuan/month	1.26 (1.00–1.60) 0.87 (0.62–1.21)	Yan Zhang et al., 2013
	Less than $20,000 $20,000–$49.999	50,000+	1.08 (0.91–1.27) 1.19 (1.03–1.37)	Drew A. Westmoreland et al., 2021
	500–999 rand/month >1000 rand/month	0–499 rand/month	0.58 (0.28–1.21) 0.82 (0.43–1.58)	Tonia Poteat et al., 2020

^a^ Willing to take once‐daily oral pill as PrEP if it could be purchased at private hospitals/clinics at HK $8000/month.

^b^ Willing to take once‐daily oral pill as PrEP if free PrEP could be provided by public hospitals/clinics in Hong Kong.

^c^ On‐demand use of PrEP.

^d^ Daily use of PrEP.

^e^ Model 1.

^f^ Model 2.

^g^ Model 3.

**Table A3:** Quality assessment for quantitative studies

Study	Selection bias	Design	Confounders	Blinding	Data collection methods	Withdrawals and drop‐outs	Global rating
Adamma Aghaizu et al., 2012	Moderate	Weak	Strong	Weak	Moderate	Not applicable	Weak
Adams, J. W. et al., 2016	Moderate	Weak	Weak	Weak	Moderate	Not applicable	Weak
Adedotun Ogunbajo et al., 2019	Moderate	Weak	Strong	Weak	Moderate	Not applicable	Weak
Adedotun Ogunbajo et al., 2019	Moderate	Weak	Moderate	Weak	Moderate	Not applicable	Weak
Alberto Edeza et al., 2019	Moderate	Weak	Weak	Weak	Moderate	Not applicable	Weak
Alia A. et al., 2014	Moderate	Weak	Moderate	Weak	Moderate	Not applicable	Weak
Alvin Gordián‐Arroyo et al., 2020	Moderate	Weak	Weak	Weak	Moderate	Not applicable	Weak
Ana Wheelock et al., 2013	Moderate	Weak	Moderate	Weak	Moderate	Not applicable	Weak
Ashley Schuyler et al., 2021	Strong	Weak	Weak	Weak	Strong	Not applicable	Weak
Ashwin Belludi et al., 2021	Moderate	Weak	Moderate	Weak	Moderate	Not applicable	Weak
Athanase Munyaneza et al., 2021	Moderate	Weak	Weak	Weak	Strong	Not applicable	Weak
Ava Lorenc et al., 2021	Weak	Weak	Weak	Weak	Moderate	Not applicable	Weak
Ayala et al., 2013	Moderate	Weak	Moderate	Weak	Strong	Not applicable	Weak
B. Hampel et al., 2017	Moderate	Weak	Weak	Weak	Moderate	Not applicable	Weak
B. Lebouché et al., 2015	Strong	Weak	Strong	Weak	Moderate	Not applicable	Weak
Benjamin B. Strauss et al., 2017	Moderate	Weak	Weak	Weak	Moderate	Not applicable	Weak
Bridget L. Draper et al., 2017	Moderate	Weak	Strong	Weak	Moderate	Not applicable	Weak
Brooke E. Hoots et al., 2016	Moderate	Weak	Strong	Weak	Weak	Not applicable	Weak
Carin Ahouada et al., 2020	Moderate	Weak	Weak	Weak	Moderate	Not applicable	Weak
Carlos Iniesta et al., 2018	Moderate	Weak	Strong	Weak	Moderate	Not applicable	Weak
Catherine E. Oldenburg et al., 2014	Moderate	Weak	Moderate	Weak	Weak	Not applicable	Weak
Charlotte‐Paige Rolle et al., 2017	Moderate	Moderate	Strong	Weak	Moderate	Strong	Moderate
Charlotte‐Paige Rolle et al., 2018	Moderate	Weak	Strong	Weak	Weak	Not applicable	Weak
Christian Grov et al., 2015	Moderate	Moderate	Moderate	Weak	Moderate	Not applicable	Moderate
Christian Grov et al., 2016	Moderate	Moderate	Moderate	Weak	Moderate	Not applicable	Moderate
Christoph D. Spinner et al., 2018	Moderate	Weak	Weak	Weak	Moderate	Not applicable	Weak
CK Uthappa et al., 2017	Moderate	Weak	Moderate	Weak	Moderate	Not applicable	Weak
Cristian J. Chandler et al., 2021	Moderate	Weak	Weak	Weak	Moderate	Not applicable	Weak
Cristian J. Chandler et al., 2021	Moderate	Weak	Weak	Weak	Moderate	Not applicable	Weak
Daniel Yang et al., 2013	Strong	Weak	Strong	Weak	Moderate	Not applicable	Weak
Darcy White Rao et al., 2019	Moderate	Weak	Strong	Weak	Moderate	Not applicable	Weak
David A. Moskowitz et al., 2020	Strong	Weak	Weak	Weak	Strong	Not applicable	Weak
Deng‐Min Chuang et al., 2018	Moderate	Weak	Strong	Weak	Moderate	Not applicable	Weak
Dou Qu et al., 2018	Moderate	Moderate	Moderate	Weak	Strong	Not applicable	Moderate
Douglas S. Krakower et al., 2012	Moderate	Weak	Strong	Weak	Weak	Not applicable	Weak
Drew A. Westmoreland	Strong	Moderate	Strong	Weak	Moderate	Weak	Weak
Driver R et al., 2020	Moderate	Weak	Weak	Weak	Moderate	Not applicable	Weak
Eisingerich et al., 2012	Strong	Weak	Strong	Weak	Moderate	Not applicable	Weak
Elizabeth Anne Barash et al., 2010	Moderate	Weak	Moderate	Weak	Moderate	Not applicable	Weak
Elske Marra et al., 2015	Strong	Weak	Moderate	Weak	Moderate	Not applicable	Weak
Erik D. Storholm et al., 2019	Moderate	Moderate	Moderate	Weak	Moderate	Not applicable	Moderate
Erin C. Wilson et al., 2020	Strong	Weak	Weak	Weak	Weak	Not applicable	Weak
Erin C. Wilson et al., 2021	Moderate	Weak	Moderate	Weak	Moderate	Not applicable	Weak
Evelyn Olansky et al., 2019	Strong	Weak	Weak	Weak	Strong	Not applicable	Weak
Fabiane Soares et al., 2019	Moderate	Weak	Strong	Weak	Moderate	Not applicable	Weak
Feng Zhou et al., 2012	Strong	Moderate	Moderate	Weak	Moderate	Not applicable	Moderate
Gianluca Voglino et al., 2021	Strong	Weak	Moderate	Weak	Strong	Not applicable	Weak
Gordon Mansergh et al., 2018	Moderate	Weak	Moderate	Weak	Weak	Not applicable	Weak
Hoagland et al., 2016	Moderate	Weak	Strong	Weak	Moderate	Not applicable	Weak
Hyun‐Ha Chang et al., 2018	Moderate	Weak	Weak	Weak	Weak	Not applicable	Weak
Ian W. Holloway et al., 2017	Moderate	Weak	Strong	Weak	Moderate	Not applicable	Weak
Ingrid Young et al., 2013	Moderate	Weak	Strong	Weak	Moderate	Not applicable	Weak
J.M. Hugo et al., 2016	Moderate	Weak	Weak	Weak	Moderate	Not applicable	Weak
James A. Griffin et al., 2020	Moderate	Weak	Weak	Weak	Moderate	Not applicable	Weak
James Wilton et al., 2015	Moderate	Weak	Strong	Weak	Moderate	Not applicable	Weak
Jamie Frankis et al., 2016	Strong	Weak	Strong	Weak	Moderate	Not applicable	Weak
Jamie S. Frankis et al., 2015	Moderate	Weak	Moderate	Weak	Moderate	Not applicable	Weak
Janneke P. Bil et al., 2015	Strong	Weak	Strong	Weak	Moderate	Not applicable	Weak
Jayoti Rana et al., 2018	Moderate	Weak	Moderate	Weak	Weak	Not applicable	Weak
Jeffrey Morgan et al., 2018	Moderate	Weak	Moderate	Weak	Moderate	Not applicable	Weak
Jeffrey T. Parsons et al., 2016	Moderate	Weak	Moderate	Weak	Moderate	Not applicable	Weak
Jeffrey T. Parsons et al., 2017	Strong	Moderate	Weak	Weak	Moderate	Strong	Weak
Jessica L. Maksut et al., 2020	Strong	Weak	Moderate	Weak	Strong	Not applicable	Weak
Jessica Londeree Saleska et al., 2020	Moderate	Weak	Weak	Weak	Moderate	Not applicable	Weak
Jesus Peinado et al., 2013	Moderate	Weak	Weak	Weak	Moderate	Not applicable	Weak
Jiatong He et al., 2014	Strong	Weak	Moderate	Weak	Weak	Not applicable	Weak
Jing Han et al., 2019	Moderate	Weak	Strong	Weak	Moderate	Not applicable	Weak
Jingjing Li et al., 2018	Moderate	Weak	Strong	Weak	Moderate	Not applicable	Weak
Jingzhen Lai et al., 2020	Moderate	Weak	Strong	Weak	Moderate	Not applicable	Weak
Joanne E. Mantell et al., 2014	Moderate	Weak	Weak	Weak	Weak	Not applicable	Weak
Johannes Bullinger et al., 2019	Moderate	Weak	Moderate	Weak	Moderate	Not applicable	Weak
Jonathan M. Galka et al., 2020	Moderate	Weak	Weak	Weak	Moderate	Not applicable	Weak
Jonathan P. Feelemyer et al., 2021	Moderate	Weak	Weak	Weak	Moderate	Not applicable	Weak
Kathrine Meyers et al., 2017	Moderate	Moderate	Strong	Weak	Moderate	Not applicable	Moderate
Kathryn Macapagal et al., 2020	Weak	Weak	Weak	Weak	Weak	Not applicable	Weak
Kathryn Macapagal et al., 2019	Strong	Weak	Strong	Weak	Moderate	Not applicable	Weak
Katie B. Biello et al., 2018	Moderate	Weak	Weak	Weak	Moderate	Not applicable	Weak
L. Ferrer et al., 2016	Strong	Weak	Strong	Weak	Moderate	Not applicable	Weak
Laio Magno et al., 2019	Moderate	Weak	Weak	Weak	Weak	Not applicable	Weak
Li Yan et al., 2020	Strong	Weak	Strong	Weak	Moderate	Not applicable	Weak
Liping Peng et al., 2019	Strong	Weak	Strong	Weak	Moderate	Not applicable	Weak
Lisa A. Eaton et al., 2015	Moderate	Weak	Moderate	Weak	Weak	Not applicable	Weak
Lisa A. Eaton et al., 2017	Moderate	Weak	Strong	Weak	Moderate	Not applicable	Weak
Lisa A. Eaton et al., 2014	Moderate	Weak	Moderate	Weak	Moderate	Not applicable	Weak
Lisa A. Eaton et al., 2017	Moderate	Weak	Moderate	Weak	Moderate	Not applicable	Weak
Liza Coyer et al., 2018	Strong	Moderate	Weak	Weak	Moderate	Weak	Weak
Long Hoang Nguyen et al., 2021	Moderate	Weak	Moderate	Weak	Moderate	Not applicable	Weak
Lori M. Wardet, 2019	Moderate	Weak	Moderate	Weak	Moderate	Not applicable	Weak
Makobu Kimani et al., 2017	Moderate	Moderate	Moderate	Weak	Moderate	Not applicable	Moderate
Mao et al., 2017	Moderate	Weak	Strong	Weak	Weak	Not applicable	Weak
Marieke Bak et al., 2018	Moderate	Weak	Moderate	Weak	Moderate	Not applicable	Weak
Mart van Dijk et al., 2020	Moderate	Weak	Moderate	Weak	Moderate	Not applicable	Weak
Martin Holt et al., 2020	Moderate	Weak	Weak	Weak	Moderate	Not applicable	Weak
Martin Holt et al., 2017	Moderate	Weak	Strong	Weak	Moderate	Not applicable	Weak
Matthew C. Sullivan et al., 2020	Weak	Weak	Moderate	Weak	Weak	Not applicable	Weak
Matthew E. Levy et al., 2017	Moderate	Weak	Strong	Weak	Moderate	Not applicable	Weak
Matthew J. Mimiaga et al., 2009	Moderate	Weak	Strong	Weak	Strong	Not applicable	Weak
Matthew R. Beymer et al., 2018	Moderate	Weak	Strong	Weak	Moderate	Not applicable	Weak
Maya A. Kesler et al., 2016	Moderate	Weak	Strong	Weak	Moderate	Not applicable	Weak
Meagan Zarwell et al., 2019	Moderate	Weak	Moderate	Weak	Moderate	Not applicable	Weak
Nai‐Ying Ko et al., 2016	Strong	Weak	Moderate	Weak	Moderate	Not applicable	Weak
Nicolas Lorente et al., 2011	Moderate	Weak	Strong	Weak	Moderate	Not applicable	Weak
Ofole Mgbako et al., 2018	Moderate	Weak	Weak	Weak	Moderate	Not applicable	Weak
Patrick S. Sullivan et al., 2020	Weak	Weak	Moderate	Weak	Moderate	Not applicable	Weak
Pierre‐julien Coulaud et al., 2018	Moderate	Weak	Strong	Weak	Weak	Not applicable	Weak
R. Craig Sineath et al., 2013	Moderate	Weak	Weak	Weak	Moderate	Not applicable	Weak
Raiza M. Beltran et al., 2021	Moderate	Weak	Weak	Weak	Moderate	Not applicable	Weak
Rayner Kay Jin Tan et al., 2018	Moderate	Weak	Weak	Weak	Moderate	Not applicable	Weak
Reshmie A. Ramautarsing et al., 2020	Moderate	Weak	Strong	Weak	Strong	Not applicable	Weak
Ricardo Niklas Werner et al., 2018	Moderate	Weak	Strong	Weak	Moderate	Not applicable	Weak
Richard A. Crosby et al., 2014	Moderate	Weak	Weak	Weak	Moderate	Not applicable	Weak
Rissa Cempaka et al., 2020	Moderate	Weak	Moderate	Weak	Moderate	Not applicable	Weak
Rob Stephenson et al., 2021	Strong	Weak	Moderate	Weak	Moderate	Not applicable	Weak
Rob Stephenson et al., 2021	Weak	Weak	Strong	Weak	Moderate	Not applicable	Weak
Robinson Njoroge Karuga et al., 2016	Moderate	Weak	Moderate	Weak	Moderate	Not applicable	Weak
Ronald A. Brooks et al., 2015	Moderate	Weak	Strong	Weak	Moderate	Not applicable	Weak
Ronald A. Brooks et al., 2019	Moderate	Weak	Strong	Weak	Moderate	Not applicable	Weak
Rudy Patrick et al., 2017	Moderate	Weak	Strong	Weak	Moderate	Not applicable	Weak
Ryan J. Watson et al., 2019	Moderate	Weak	Weak	Weak	Moderate	Not applicable	Weak
Sarah M. Wood et al., 2016	Moderate	Weak	Weak	Weak	Moderate	Not applicable	Weak
Sarah M. Wood et al., 2020	Strong	Weak	Weak	Weak	Strong	Not applicable	Weak
Sari L. Reisner et al., 2021	Strong	Weak	Weak	Weak	Moderate	Not applicable	Weak
Shufang Sun et al., 2021	Moderate	Weak	Strong	Weak	Strong	Not applicable	Weak
Sibylle Nideröst et al., 2018	Moderate	Weak	Weak	Weak	Moderate	Not applicable	Weak
Sin How Lim et al., 2017	Weak	Weak	Strong	Weak	Moderate	Not applicable	Weak
Steven A. Elsesser et al., 2015	Moderate	Weak	Moderate	Weak	Moderate	Not applicable	Weak
Susan Fallon, 2015	Moderate	Weak	Moderate	Weak	Moderate	Not applicable	Weak
Teresa Finlayson et al., 2019	Moderate	Weak	Strong	Weak	Weak	Not applicable	Weak
Thiago Silva Torres et al., 2018	Moderate	Weak	Moderate	Weak	Moderate	Not applicable	Weak
Tobias Herder et al., 2020	Moderate	Weak	Strong	Weak	Moderate	Not applicable	Weak
Tonia Poteat et al., 2020	Moderate	Weak	Moderate	Weak	Moderate	Not applicable	Weak
Tsz Ho Kwan et al., 2019	Moderate	Weak	Strong	Weak	Moderate	Not applicable	Weak
Udodirim Onwubiko et al., 2020	Strong	Weak	Strong	Weak	Moderate	Not applicable	Weak
Vanessa M. McMahan et al., 2020	Strong	Weak	Weak	Weak	Weak	Not applicable	Weak
Venkatesan Chakrapani, 2021	Moderate	Weak	Strong	Weak	Moderate	Not applicable	Weak
Wei et al., 2011	Strong	Weak	Weak	Weak	Moderate	Not applicable	Weak
Weiying Chen et al., 2021	Moderate	Weak	Moderate	Weak	Moderate	Not applicable	Weak
Wenting Huang et al., 2019	Moderate	Weak	Moderate	Weak	Moderate	Not applicable	Weak
William C. Goedel et al., 2016	Moderate	Weak	Moderate	Weak	Moderate	Not applicable	Weak
Xia Wang et al., 2018	Weak	Weak	Weak	Weak	Moderate	Not applicable	Weak
Xie et al., 2017	Strong	Weak	Moderate	Weak	Moderate	Not applicable	Weak
Xue et al., 2015	Strong	Weak	Moderate	Weak	Moderate	Not applicable	Weak
Yan Zhang et al., 2013	Moderate	Weak	Strong	Weak	Moderate	Not applicable	Weak
Yingying Ding et al., 2016	Moderate	Weak	Moderate	Weak	Moderate	Not applicable	Weak
Yuansheng Fu et al., 2021	Moderate	Weak	Moderate	Weak	Moderate	Not applicable	Weak
Zhi‐Wei Zheng et al., 2018	Moderate	Weak	Moderate	Weak	Strong	Not applicable	Weak
Zhong et al., 2013	Strong	Weak	Moderate	Weak	Moderate	Not applicable	Weak
Zhuang Cui et al., 2020	Moderate	Weak	Moderate	Weak	Moderate	Not applicable	Weak
Zixin Wang et al., 2018	Moderate	Weak	Moderate	Weak	Moderate	Not applicable	Weak
Zixin Wang et al., 2020	Strong	Weak	Moderate	Weak	Moderate	Not applicable	Weak

**Table A4:** Quality assessment for qualitative studies

Study	Clear statement of research aims	Qualitative methodology appropriate	Research design appropriate to address research aims	Recruitment strategy appropriate to address research aims?	Data collected in a way that addressed research issue	Relationship between researcher and participants adequately considered	Ethical issues taken into consideration	Data analysis sufficiently rigorous	Clear statement of findings	Research valuable	Score	Quality rating
Carin Ahouada et al., 2019	Yes	Yes	Can't tell	Yes	Yes	Can't tell	Yes	Yes	Yes	Yes	8	Strong
Elizabeth Mpunga et al., 2020	Yes	Yes	Can't tell	Yes	Yes	Can't tell	Yes	Yes	Yes	Yes	8	Strong
Hillis A. et al., 2021	Yes	Yes	Can't tell	Yes	Yes	Can't tell	Yes	Yes	Yes	Yes	8	Strong
Long Hoang Nguyen et al., 2021	Yes	Yes	Can't tell	Yes	Yes	Can't tell	Yes	Yes	Yes	Yes	8	Strong
Venkatesan Chakrapani et al., 2015	Yes	Can't tell	Can't tell	Yes	Yes	Can't tell	No	Yes	Yes	Yes	6	Moderate
Venkatesan Chakrapani et al., 2020	Yes	Can't tell	Can't tell	Yes	Yes	Can't tell	Yes	Yes	Yes	Yes	7	Moderate
