# Supplementary material for: Increasing awareness of HIV pre‐exposure prophylaxis (PrEP) and willingness to use HIV PrEP among men who have sex with men: a systematic review and meta‐analysis of global data
Source: J Int AIDS Soc. 2022 Mar 7;25(3):e25883. doi: 10.1002/jia2.25883 (PMC8901150; doi:10.1002/jia2.25883)
Supplement: Supplementary file 2 — File S2. Detailed search strings [file JIA2-25-e25883-s002.docx]

**Search terms for PubMed**

((intention OR intentional OR intent OR intend OR willingness OR will OR utilization OR utilisation OR acceptance OR acceptability OR adoption OR uptake OR usage OR use OR awareness OR adherence OR knowledge OR attitude OR perception OR feasibility)) AND (pre-exposure prophylaxis OR preexposure prophylaxis OR "PrEP" OR HIV pre-exposure prophylaxis OR HIV preexposure prophylaxis OR pre-exposure antiretroviral prophylaxis OR pre exposure antiretroviral prophylaxis OR pre-exposure chemoprophylaxis OR preexposure chemoprophylaxis OR anti-HIV prophylaxis) AND((men who have sex with men OR MSM OR gay OR bisexual OR transgender OR transgender women OR transwomen OR high risk behavio* OR high risk group* OR key population*[Mesh])) AND (human immunodeficiency virus[Mesh] OR HIV[Mesh] OR HIV-positive[Mesh] OR acquired immunodeficiency syndrome OR AIDS OR HIV/AIDS)

**Search terms for Embase**

('intention'/exp OR 'intention' OR 'intentional' OR 'intent' OR 'intend' OR 'will'/exp OR 'will' OR 'willingness'/exp OR 'willingness' OR 'utilization'/exp OR 'utilization' OR utilisation OR 'acceptance'/exp OR 'acceptance' OR 'acceptability'/exp OR 'acceptability' OR 'adoption'/exp OR 'adoption' OR uptake OR usage OR use OR 'awareness'/exp OR 'awareness' OR 'adherence'/exp OR 'adherence' OR 'knowledge'/exp OR 'knowledge' OR 'attitude'/exp OR 'attitude' OR 'perception'/exp OR 'perception' OR 'feasibility study'/exp OR 'feasibility study') AND ('pre-exposure prophylaxis'/exp OR 'pre-exposure prophylaxis' OR prep OR 'hiv'/exp OR hiv) AND ('pre-exposure prophylaxis'/exp OR 'pre-exposure prophylaxis' OR 'preexposure antiretroviral prophylaxis' OR 'pre-exposure chemoprophylaxis' OR 'preexposure chemoprophylaxis' OR 'anti-hiv prophylaxis') AND ('men who have sex with men'/exp OR 'men who have sex with men' OR 'homosexual male'/exp OR 'homosexual male' OR 'bisexuality'/exp OR 'bisexuality' OR 'transgender'/exp OR 'transgender' OR 'transgender women' OR 'male to female transgender'/exp OR 'male to female transgender' OR 'high risk behavior'/exp OR 'high risk behavior' OR 'high risk population'/exp OR 'high risk population' OR 'key population') AND ('human immunodeficiency virus'/exp OR 'human immunodeficiency virus' OR 'hiv'/exp OR hiv OR 'human immunodeficiency virus infected patient'/exp OR 'human immunodeficiency virus infected patient' OR 'acquired immune deficiency syndrome'/exp OR 'acquired immune deficiency syndrome' OR 'aids'/exp OR aids)

**Search terms for Web of science**

((TS=(intention OR intentional OR intent OR intend OR willingness OR will OR utilization OR utilisation OR acceptance OR acceptability OR adoption OR uptake OR usage OR use OR awareness OR adherence OR knowledge OR attitude OR perception OR feasibility)) AND (TS=(pre-exposure prophylaxis OR preexposure prophylaxis OR "PrEP" OR HIV pre-exposure prophylaxis OR HIV preexposure prophylaxis OR pre-exposure antiretroviral prophylaxis OR preexposure antiretroviral prophylaxis OR pre-exposure chemoprophylaxis OR preexposure chemoprophylaxis OR anti-HIV prophylaxis))AND (TS=(men who have sex with men OR MSM OR gay OR bisexual OR transgender OR transgender women OR transwomen OR high risk behavior OR high risk group OR key population)) AND (TS=(human immunodeficiency virus OR HIV OR HIV-positive OR acquired immunodeficiency syndrome OR AIDS OR HIV/AIDS)))

**Search terms for Cochrane Library**

#1 MeSH descriptor: [HIV] this term only

#2 human immunodeficiency virus OR HIV-positive OR acquired immunodeficiency syndrome OR AIDS: ti,ab,kw

#3 #1 OR #2

#4 MeSH descriptor: [Pre-Exposure Prophylaxis] this term only

#5 pre-exposure prophylaxis OR preexposure prophylaxis OR PrEP OR HIV pre-exposure prophylaxis OR HIV preexposure prophylaxis OR pre-exposure antiretroviral prophylaxis OR preexposure antiretroviral prophylaxis OR pre-exposure chemoprophylaxis OR preexposure chemoprophylaxis OR anti-HIV prophylaxis: ti,ab,kw

#6 #4 OR #5

#7 MeSH descriptor: [Homosexuality,Male] this term only

#8 men who have sex with men OR MSM OR gay OR bisexual OR transgender OR transgender women OR transwomen OR high risk behavio* OR high risk group* OR key population*: ti,ab,kw

#9 #7 OR #8

#10 intention OR intentional OR intent OR intend OR willingness to use OR willingness to take OR utilization OR utilisation OR acceptance OR acceptability OR adoption OR uptake OR usage OR use: ti,ab,kw

#11 awareness OR adherence OR knowledge OR attitude OR perception OR feasibility: ti,ab,kw

#12 #10 OR #11

#13 #3 AND #6 AND #9 AND #12
